# SwiftOrtho: A fast, memory-efficient, multiple genome orthology classifier

**DOI:** 10.1093/gigascience/giz118

**Published:** 2019-10-24

**Authors:** Xiao Hu, Iddo Friedberg

**Affiliations:** Department of Veterinary Microbiology and Preventive Medicine, 2118 Veterinary Medicine, College of Veterinary Medicine, Iowa State University, Ames, IA, 50011, USA

**Keywords:** orthology analysis, homology search, orthology inference, clustering, orthologs, paralogs

## Abstract

**Background:**

Gene homology type classification is required for many types of genome analyses, including comparative genomics, phylogenetics, and protein function annotation. Consequently, a large variety of tools have been developed to perform homology classification across genomes of different species. However, when applied to large genomic data sets, these tools require high memory and CPU usage, typically available only in computational clusters.

**Findings:**

Here we present a new graph-based orthology analysis tool, SwiftOrtho, which is optimized for speed and memory usage when applied to large-scale data. SwiftOrtho uses long *k*-mers to speed up homology search, while using a reduced amino acid alphabet and spaced seeds to compensate for the loss of sensitivity due to long *k*-mers. In addition, it uses an affinity propagation algorithm to reduce the memory usage when clustering large-scale orthology relationships into orthologous groups. In our tests, SwiftOrtho was the only tool that completed orthology analysis of proteins from 1,760 bacterial genomes on a computer with only 4 GB RAM. Using various standard orthology data sets, we also show that SwiftOrtho has a high accuracy.

**Conclusions:**

SwiftOrtho enables the accurate comparative genomic analyses of thousands of genomes using low-memory computers. SwiftOrtho is available at https://github.com/Rinoahu/SwiftOrtho

## Background

Gene homology type classification consists of identifying paralogs and orthologs across species. Orthologs are genes that evolved from a common ancestral gene following speciation, while paralogs are genes that are homologous owing to duplication. Paralogs can be further classified into in-paralogs, which evolved via gene duplication before the speciation event, and out-paralogs, which evolved via gene duplication after the speciation event [1]. Classifying orthologs and paralogs across species is an important problem because the evolutionary history of genes has implications for our understanding of gene function and evolution.

While the proper inference of homology type involves tracing gene history using phylogenetic trees [2], several proxy methods have been developed over the years. The most common method to infer orthologs by proxy is reciprocal best hits (RBH) [3, 4]. Briefly, RBH states the following: when 2 proteins that are encoded by 2 genes, each in a different genome, find each other as the best-scoring match among all homologs, they are considered to be orthologs [3, 4].

**Figure 1:**
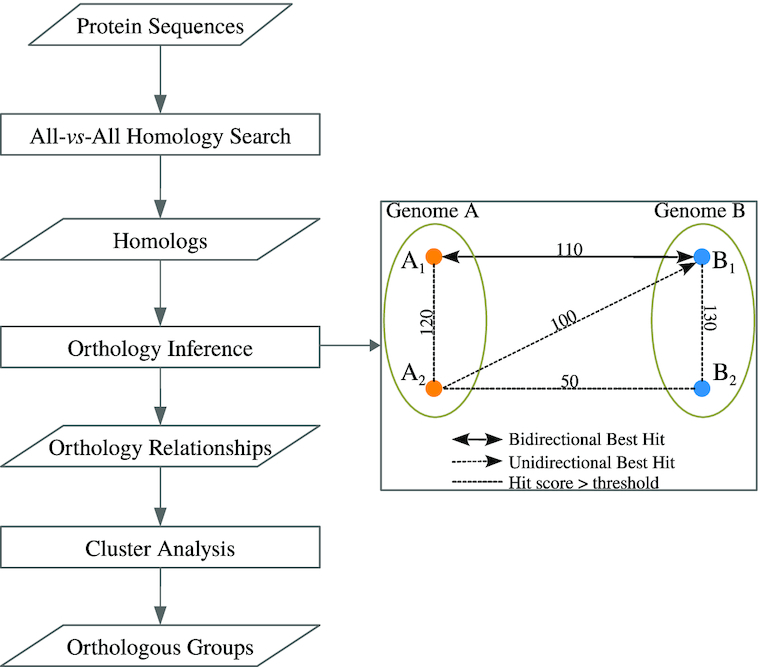
Flow chart of SwiftOrtho. SwiftOrtho is a graph-based method that consists of 3 major steps. (i) All-vs-all homology search: a seed-and-extension method is used to perform a homology search. (ii) Orthology inference: nodes are gene names; edges are similarity score of pairwise genes. 1. A_1_-B_1_ are putative orthologs identified by RBH; 2. A_1_-A_2_ and B_1_-B_2_ are putative in-paralogs because the bit scores of these pairs are greater than A_1_-B_1_; 3. A_2_-B_1_ and A_2_-B_2_ are putative co-orthologs because these pairs are not orthologs but A_1_-B_1_ are orthologs and A_1_-A_2_, B_1_-B_2_ are in-paralogs. (iii) Cluster analysis: Markov clustering or affinity propagation algorithm is used to cluster orthology relationships.

InParanoid extends the RBH orthology relationship to include both orthologs and in-paralogs. Specifically, InParanoid uses RBH to identify orthologs between 2 species. The genes in the 2 species are classified as in-paralogs if they are more similar to the corresponding ortholog than to any gene in the other species [5–7]. The concept of orthologous pairs between 2 species can be extended to an "ortholog group," which is a set of genes that are hypothesized to have descended from a common ancestor [7]. Several methods have been developed to identify ortholog groups across multiple species, typically classified as either tree-based or graph-based methods. Tree-based methods construct a gene tree from an alignment of homologous sequences in different species and infer orthology relationships by reconciling the gene tree with its corresponding species tree [2, 8, 9] and can infer a correct orthology relationship if the correct gene tree and species tree are provided [10]. The chief limiting factor of tree-based methods is the accuracy of the given gene tree and species tree. Erroneous trees lead to incorrect ortholog and in-paralog assignments [9–11]. Tree-based methods are also computationally expensive, which limits the ability to apply them to a large number of species [10, 12–14]. Graph-based methods infer orthologs and in-paralogs from homologs and then use different strategies to cluster them into orthologous groups [9, 12, 13] (Fig. 1). The Clusters of Orthologous Groups (COG) database detects triangles of RBHs in 3 different species and merges the triangles with a common side [15]. Orthologous Matrix (OMA) clusters RBHs in orthologous groups by finding maximum weight cliques from the similarity graph [16, 17]. MultiParanoid is an extension of InParanoid, which uses InParanoid to detect triangle orthologs and in-paralogs in 3 different species as seeds and then merges the seeds into larger groups [18]. OrthoMCL also uses InParanoid to detect orthologs, co-orthologs, and in-paralogs between 2 species [19, 20] and then uses Markov clustering (MCL) [21] to cluster these relationships into orthologous groups, where the co-orthologs are ≥2 genes in 1 species that are orthologous to ≥1 genes in another species due to a gene duplication event [1, 22].

Finally, there are hybrid methods that combine both graph-based and tree-based methods [12, 23–26]. Typically, hybrid methods first perform all-vs-all sequence alignment, then construct gene families by sequence similarity or conserved gene neighborhood. EnsEMBL first uses RBH to find the gene families, then constructs a phylogenetic gene tree for each gene family [24]. Finally, each gene tree is reconciled with the species tree to infer paralogs and orthologs.

In theory, graph-based methods are less accurate than tree-based methods because the former identify orthologs and in-paralogs using proxy methods rather than directly inferring homology type from gene and species evolutionary history. However, graph-based methods have been found to be comparably accurate to tree-based methods [10, 11, 27]. Moreover, a comparison of several methods found that tree-based methods had an even worse performance than graph-based methods on large data sets [11].

One study compared several common methods, including simple RBH, graph-based, tree-based, and hybrid methods, and found that the tree-based methods of InParanoid and OrthoMCL exhibit the best balance of sensitivity and specificity [28]. Several studies have also shown that graph-based methods find a better trade-off between specificity and sensitivity than tree-based methods [11, 28, 29]. For these reasons, graph-based methods are generally preferred for analyzing large-scale data sets. OrthoMCL and InParanoid have been applied to analyze hundreds of genomes; at the same time, they require considerable computational resources that may not be readily available [20, 30]. More recently, several graph-based tools, such as SonicParanoid, OMA, and ProteinOrtho [17, 31, 32], have been developed to speed up orthology analysis on large-scale data sets. These tools also tend to require high-performance computers with large memory to analyze large-scale data.

Here we present SwiftOrtho, a fast method for orthology classification that makes minimal use of computational resources, especially memory. SwiftOrtho uses a seed-and-extension method to speed up homology search, a binary search method and RBH rule to infer orthologs and in-paralogs, and the affinity propagation algorithm to reduce memory usage in cluster analysis. We compare SwiftOrtho with several existing graph-based tools using the gold standard data set Orthobench [13], and the Quest for Orthologs service [33]. Using both benchmarks, we show that SwiftOrtho provides a high accuracy with lower CPU and memory usage than other graph-based methods. SwiftOrtho is the only tool that completed an orthology analysis of 1,760 bacterial genomes on a very low-memory computer. With the growing number of genomes, especially microbial genomes, we see SwiftOrtho to be a tool of choice for a fast and accurate ortholog classification, while requiring low computational resources, as are found in conventional desktop or laptop computers.

## Application of SwiftOrtho

### Data sets

We applied SwiftOrtho to 3 data sets to evaluate its predictive quality and performance:

1. The Euk set was used to evaluate the quality of predicted orthologous groups. This set contains 420,415 protein sequences from 12 eukaryotic species, including *Caenorhabditis elegans, Drosophila melanogaster, Ciona intestinalis, Danio rerio, Tetraodon nigroviridis, Gallus gallus, Monodelphis domestica, Mus musculus, Rattus norvegicus, Canis familiaris, Pan troglodytes*, and *Homo sapiens*. The protein sequences for these genes were downloaded from EMBL v65 [34].
2. The QfO 2011 set was used to evaluate the quality of predicted orthology relationships. This set was the reference proteome data set (2011) of The Quest for Orthologs [33], which contains 754,149 protein sequences of 66 species.
3. The large Bac set was used to evaluate performance, including CPU time, real time, and RAM usage. This set includes 5,950,817 protein sequences from 1,760 bacterial species. The protein sequences were downloaded from GenBank [35]. For a full list see [64], file: readme.txt. .

We also compared SwiftOrtho with several existing orthology analysis tools for predictive quality and performance. The methods compared were OrthoMCL (v2.0), FastOrtho, OrthAgogue, and OrthoFinder.

### Orthology analysis pipeline

The pipeline for all the tools follows the standard steps of graph-based orthology prediction, (i) all-vs-all homology search, (ii) orthology inference, and (iii) cluster analysis.

#### Homology search

SwiftOrtho used its built-in module to perform all-vs-all homology search. For all 3 sets, the E-value was set 10^−5^. The amino acid alphabet was set to the regular 20 amino acids for the 3 sets. The spaced seed parameter was set to "1011111,11111" for the Euk, "11111111" for the QfO 2011, and "111111" for Bac.

OrthoMCL, FastOrtho, OrthAgogue, and OrthoFinder use BLASTP (v2.2.27+) [36] to perform all-vs-all homology search. The first 3 tools require the user to do this manually. To compare the methods, the -e (e-value), -v (number of database sequences to show one-line descriptions), and -b (number of database sequence to show alignments) parameters of BLASTP were set to 10^−5^, 1,000,000, and 1,000,000 for OrthoMCL, FastOrtho, and OrthAgogue. The OrthoFinder calls BLASTP, and the e-value of BLASTP has been set to 10^−3^.

#### Orthology inference

SwiftOrtho, OrthoMCL, FastOrtho, OrthAgogue, and OrthoFinder were applied to perform orthology inference on the homologs. The first 4 tools are able to identify (co-)orthologs and in-paralogs, and the coverage (fraction of aligned regions) was set to 50%, while other parameters were set to their default values (see Supplementary Materials section 4.2. for full details).

FastOrtho does not report (co-)orthologs and in-paralogs directly. However, the relevant information is stored in an intermediate file, from which we have extracted that information. Orthofinder does not report orthology relationships.

#### Cluster analysis

All the tools in this study use MCL [21] for clustering. To control the granularity of the clustering, MCL performs an inflation operation set by the *-I*option [21, 37]. In this study, *-I* was set to 1.5. To take advantage of multiprocessor capabilities, we set the thread number of MCL to 12. SwiftOrtho has an alternative clustering algorithm (Affinity Propagation Cluster [APC]), which we have also applied to Euk and Bac.

### Evaluation of prediction quality

#### Evaluation of predicted orthologous groups

The OrthoBench set was used to evaluate the quality of predicted orthologous groups in Bac. This set contains 70 manually curated orthologous groups of the 12 species from Bac and has been used as a high-quality gold standard benchmark set for orthologous group prediction [13]. We used OrthoBench v2 (Supplementary Table S1). Each manually curated group of the OrthoBench v2 set finds the best match in the predicted orthologous groups, where the best match means that the number of genes shared between manually curated and predicted orthologs is maximized, and the method to calculate precision and recall is shown in Supplementary Figure S1.

#### Evaluation of predicted orthology relationships

The Quest of Orthologs web-based service (QfO) was used to evaluate the quality of the orthology relationships predicted from the QfO 2011 set [33]. The QfO service evaluates the predictive quality by performing 4 phylogeny-based tests, Species Tree Discordance Benchmark, Generalized Species Tree Discordance Benchmark, Agreement with Reference Gene Phylogenies: SwissTree, and Agreement with Reference Gene Phylogenies: TreeFam-A, and 2 function-based tests, Gene Ontology conservation test and Enzyme Classification conservation test [33].

We also applied two more orthology prediction tools, SonicParanoid [31] and InParanoid (v4.1) [5], on the QfO 2011 set and used their results as control because InParanoid has the best performance among the results from the QfO service website and SonicParanoid is a fast implementation of InParanoid. The pairwise orthology relationships were extracted from the predicted orthologous groups of all the tools, including SonicParanoid and InParanoid, and then submitted to the QfO web service for further evaluation.

### Hardware

Unless specified otherwise, all tests were run on the Condo cluster of Iowa State University with Intel Xeon E5-2640 v3 at 2.60 GHz, 128 GB RAM, 28 TB free disk. The Linux command "time -v" was used to track CPU and peak memory usage.

## Findings

We compared the orthology analysis performance of SwiftOrtho, OrthoMCL, FastOrtho, OrthAgogue, and OrthFinder using Euk, QfO 2011, and Bac. The orthology analysis consisted of homology search, orthology inference, and cluster analysis.

### Orthology analysis on Euk

The results of orthology analysis on Euk are summarized in Table 1 and are elaborated upon below.

**Table 1.** Comparative orthology analysis on the Euk set

		SwiftOrtho		OrthoMCL	FastOrtho	OrthAgogue	OrthoFinder
Homology search	Method	SwiftOrtho built-in		BLASTP			
	Hits	162,695,330		947,203,546			654,792,861
	Unique Hits	162,695,330		297,107,872			266,104,611
Orthology	(Co-)orthologs	1,422,920		8,279,424	3,297,613	1,265,553	N/A
inference	In-paralogs	631,033		2,517,166	2,546,296	759,989	N/A
Clustering	Algorithm	MCL	APC	MCL			
	Orthologous Groups	44,551	38,748	36,901	40,943	51,297	19,904

APC: Affinity Propagation Cluster; MCL: Markov clustering; N/A: not available.

#### Homology search

The homology search results show that BLASTP detected the largest number of homologs, 947,203,546. SwiftOrtho found 57.50% of the homologs detected by BLASTP but was 38.7 times faster than BLASTP. SwiftOrtho used longer *k*-mers, which reduced both specific and non-specific seed extension. The longer *k*-mers cause seed-and-extension methods to ignore sequences with low similarity. According to the RBH rule, orthologs should have higher similarity than non-orthologs, so the decrease in homolgs of SwiftOrtho does not significantly affect the next orthology inference.

We compared RBHs inferred from homologs detected by BLASTP and SwiftOrtho, and the numbers of RBHs for BLASTP and SwiftOrtho were 899,473 and 957,387, respectively. Identical RBHs were 767,884 (85.37% of BLASTP). These results show that although SwiftOrtho found fewer homologs than BLASTP, it did not significantly reduce the number of RBHs. The following results in Fig. 3 also show that there is no significant difference between SwiftOrtho and BLASTP in predicting orthologous groups. Homology searches against a large number of protein sequences are a major bottleneck in bioinformatics pipelines. For that reason, many tools have been developed to speed up this process including, among others, BLAT, Usearch, LAST, DIAMOND, and Topaz [38–42]. All these tools use longer *k*-mers than BLASTP to speed up performance. We also compared SwiftOrtho with them in speed and sensitivity (Supplementary Table S9). Because BLASTP is widely considered the gold standard for comparing protein sequences, we use its results as the benchmark to evaluate the sensitivity of other homology search tools. We found Usearch and LAST to be the fastest; however, they only found 0.88% and 2.97% of BLASTP's hits, respectively. Topaz and BLAT used the most CPU time but found only 33.48% and 28.34% of the BLASTP hits, respectively. SwiftOrtho and DIAMOND (more sensitive mode) had the highest sensitivity and found 52.72% and 58.30% of the BLASTP hits in a moderate amount of time, respectively. These results show that SwiftOrtho delivers a good trade-off between speed and sensitivity.

#### Orthology inference

OrthoMCL and FastOrtho found more orthology relationships than SwiftOrtho and OrthAgogue. This is because OrthoMCL and FastOrtho use the negative log ratio of the e-value as the edge-weighting metric. The BLASTP program rounds e-value <10^−180^ to 0. Consequently, for homolgs with an e-value <10^−180^, OrthoMCL and FastOrtho treat them as the RBHs, overestimating the number of orthologs. An example showing the OrthoMCL and FastOrtho overestimation can be found in Table S4.

#### Use of computational resources

OrthoMCL v2.0 used the most CPU time and real time because of the required input/output (I/O) operations. The RAM usage of OrthoMCL was 3.45 GB, while the generated intermediate file occupied >19 TB of disk space. OrthAgogue was the most efficient in real time, because of its ability to exploit a multi-core processor. However, the RAM usage of OrthAgogue was >100 GB, which exceeds that of common workstations and many servers. The orthology inference module of FastOrtho was the most memory efficient among all the tools and was also fast. SwiftOrtho was the most CPU time efficient, although its real time was twice as that of OrthAgogue. Because the orthology inference module of SwiftOrtho was written in pure Python, we retested it by using the PyPy interpreter, an alternate implementation of Python [43]. When running with PyPy the real run time of SwiftOrtho was close to that of OrthAgogue (Table S5).

#### Cluster analysis

OrthoFinder identified the smallest number of orthologous groups. Other tools identified many more orthologous groups than OrthoFinder, ranging from 36,901 to 51,297. The APC algorithm found fewer clusters than the MCL algorithm.

#### Evaluation of predicted orthologous groups

**Figure 2:**
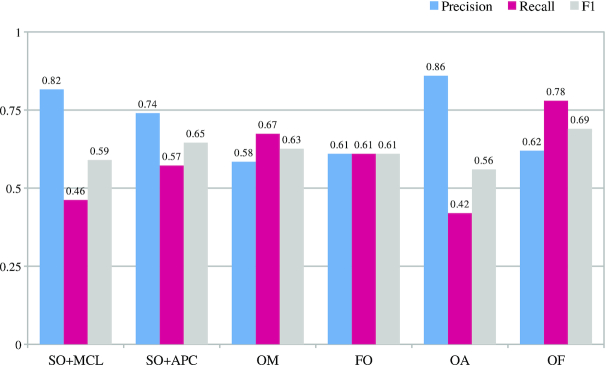
Evaluation of predicted orthologous groups. Evaluation of different tools on OrthoBench database. SO+MCL: SwiftOrtho with MCL; SO+APC: SwiftOrtho with Affinity Propagation Clustering; OM: OrthoMCL v2; FO: FastOrtho; OA: OrthAgogue; OF: OrthoFinder.

The quality of predicted orthologous groups is shown in Fig. 2. OrthoFinder had the best recall, while SwiftOrtho and OrthAgogue had top precision values but lower recall values than other tools. Because SwiftOrtho and OrthAgogue use a more stringent standard to perform orthology inference, this strategy often increases precision but decreases recall [11, 28, 29].

**Figure 3:**
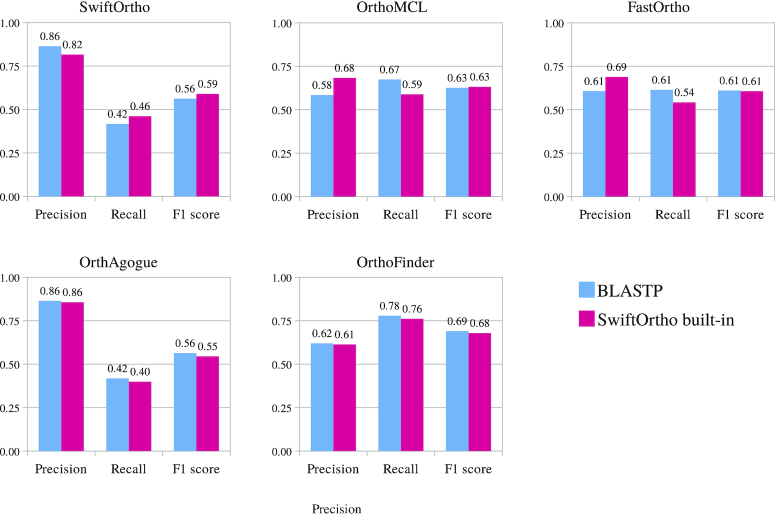
Comparing BLASTP and SwiftOrtho’s homology search module on the quality of orthologous group prediction. BLASTP and SwiftOrtho’s search module performed an all-vs-all search on the Euk set, respectively. Then, all the orthology prediction tools were used for orthology inference. Finally, the predicted orthology relationships were clustered into orthologous groups by MCL algorithm.

Because SwiftOrtho uses its built-in homology search module and its recall is lower than BLASTP’s, it may reduce the recall of orthologous groups. To address this problem, we made 2 replacements. We replaced SwiftOrtho’s homology module with BLASTP for SwiftOrtho and replaced BLASTP with SwiftOrtho’s homology module for OrthoMCL, FastOrtho, OrthAgogue, and OrthoFinder. We then reran the orthology analysis on Euk. The results show that for most tools, replacing BLASTP with SwiftOrtho’s built-in homology search module does not significantly reduce the recall (Fig. 3). The difference in recall between using SwiftOrtho’s homology search and using BLASTP is <4% except for OrthoMCL and FastOrtho. The recall for OrthoMCL and FastOrtho decreased by 8% and 7%, respectively. The most likely reason is that the E-value of SwiftOrtho’s homology search module is more precise than that of BLASTP, which reduces the false RBHs as mentioned above. These results show that SwiftOrtho’s homology search module is a reliable and fast alternative to BLASTP.

**Figure 4:**
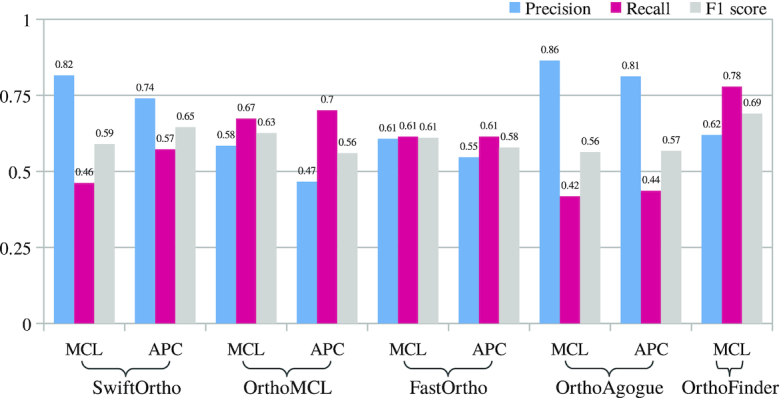
Markov clustering (MCL) versus Affinity Propagation Clustering (APC). Both algorithms were applied to cluster the orthology relationships of the Euk set inferred by different orthology prediction tools into orthologous groups. Because OrthoFinder does not report orthology relationships, the affinity propagation cannot be applied to its results.

To test the differences exhibited by the clustering component of SwiftOrtho, we ran SwiftOrtho with MCL and APC on the same data. The results (Fig. 4) show that the performance of APC is close to that of MCL. APC improves the recall of most tools (Fig. 4). These results show that APC has a performance similar to that of the MCL algorithm and is a reliable alternative to MCL.

### Orthology analysis on QfO 2011

The results of the orthology analysis on QfO 2011 are presented in Table 2 and elaborated below.

**Table 2.** Comparative orthology analysis on the Quest for Orthologs reference proteome 2011 data set.

		SwiftOrtho	OrthoMCL	FastOrtho	OrthAgogue	OrthoFinder
Homology search	Method	SO built-in	BLASTP			
	Hits	183,883,417	642,372,369			935,579,809
	Unique Hits	183,883,417	317,333,885			462,876,579
Orthology	(Co-)orthologs	2,209,243	3,743,779	2,588,851	2,716,128	N/A
inference	In-paralogs	6,929,058	11,427,118	13,649,582	13,694,208	N/A
Clustering	Algorithm	MCL				
	Orthologous groups	60,418	50,970	55,530	50,203	166,217

MCL: Markov clustering; APC: Affinity Propagation Cluster; N/A: not available.

#### Homology search

SwiftOrtho found 183,883,417 unique hits while BLASTP found 462,876,579 unique hits. However, SwiftOrtho was ∼163 times faster than BLASTP.

#### Orthology inference

OrthoMCL found many more orthologs and co-orthologs than the other tools. SwiftOrtho found fewer in-paralogs than other available tools. The CPU time of SwiftOrtho was the least of all tools. When the PyPy interpreter was used, the real time of SwiftOrtho was also close to that of the fastest one, OrthAgogue (Supplementary Table S6).

#### Cluster analysis

Overall, the clustering numbers of SwiftOrtho, OrthoMCL, FastOrtho, and OrthAgogue were similar. However, the number of clusters found by OrthoFinder was 3 times that of other tools, and the next evaluation also shows that OrthoFinder performed poorly on QfO 2011.

#### Evaluation of predicted ortholog relationships

**Figure 5:**
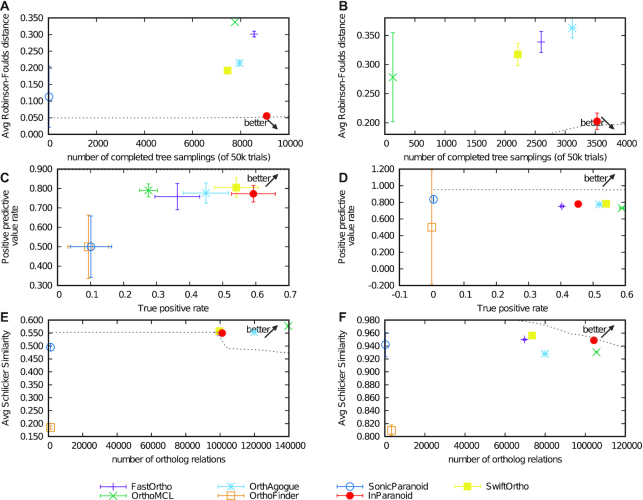
Benchmarking in Quest for Orthologs. (A) Species Tree Discordance Benchmark. InParanoid had the minimum average Robinson-Foulds distance. SwiftOrtho’s average RF distance was close to that of InParanoid. The prediction inferred by OrthoFinder was not available for this test. (B) Generalized Species Tree Discordance Benchmark. InParanoid had the minimum average Robinson-Foulds distance. The prediction inferred by OrthoFinder was not available for this test. (C) Agreement with the Reference Gene Phylogenies of SwissTree. SwiftOrtho had the highest positive prediction value rate (recall). InParanoid had the highest true-positive rate (precision). (D) Agreement with Reference Gene Phylogenies of TreeFam-A. SonicParanoid had the highest positive prediction value rate (recall); however, its true-positive rate (precision) was close to zero. SwiftOrtho had the second highest recall and precision. (E) Gene Ontology conservation test. OrthoMCL had the highest average Schlicker similarity. (F) Enzyme Classification conservation test. SwiftOrtho had the highest average Schlicker similarity. OrthoMCL detected the most orthology relationships and had the highest recall.

The evaluation shows that the performance of SwiftOrtho was close to that of InParanoid (Fig. 5). In some tests (Fig. 5D–E), SwiftOrtho outperformed InParanoid. SwiftOrtho had the best performance in the Generalized Species Tree Discordance Benchmark and Agreement with Reference Gene Phylogenies: TreeFam-A tests. In the Species Tree Discordance Benchmark, SwiftOrtho had the minimum Robinson-Foulds distance. In the Enzyme Classification (EC) conservation test, SwiftOrtho had the maximum Schlicker similarity. These 2 metrics reflect the accuracy of the algorithm, and the results show that SwiftOrtho has an overall higher accuracy than the other tools. At the same time, the recall of SwiftOrtho was lower in some of the QfO tests, the main reason being that SwiftOrtho uses a stringent metric system to identify orthology relationships.

### Orthology analysis on Bac

The results of orthology analysis on Bac are summarized in Table 3.

**Table 3.** Comparative orthology analysis on the Bac set

		SwiftOrtho		OrthoMCL	FastOrtho	OrthAgogue	OrthoFinder
Homology search	Method	SO built-in					N/A
	Hits	8,478,732,753					N/A
	Unique Hits	8,478,732,753					N/A
Orthology	(Co-)orthologs	876,766,940	N/A		950,683,849	N/A	N/A
inference	In-paralogs	622,292	N/A		663,052	N/A	N/A
Clustering	Algorithm	MCL	APC	MCL			
	Orthologous groups	240,162	167,355	N/A	242,816	N/A	N/A

MCL: Markov clustering; APC: Affinity Propagation Cluster; N/A: not available.

#### Homology search

SwiftOrtho detected 8,966,131,536 homologs in the Bac set within 1,247 CPU hours.

Because it takes a long time to perform all-vs-all BLASTP search on the full Bac, we randomly selected 1,000 protein sequences from Bac and used them to search against the full Bac set. It took BLASTP 5.1 CPU hours to find the homologs of these 1,000 protein sequences. We infer that the estimated CPU time of BLASTP on the full Bac set should be ∼30,000 CPU hours. SwiftOrtho was almost 25 times faster than BLASTP on Bac.

#### Orthology inference

SwiftOrtho, OrthoMCL, FastOrtho, and OrthAgogue were used to infer (co-)orthologs and in-paralogs from the homologs detected by the homology search module of SwiftOrtho in the Bac set. We did not test Orthofinder because Orthofinder does not accept a single file of homologs as input. For the 1,760 proteomes in Bac, OrthoFinder needs to perform 3,097,600 pairwise species-by-species comparisons, which will generate the same number of files. Then, OrthoFinder performs the orthology inference on these 3,097,600 files. Even at 1 minute per file, it will take an estimated 6 CPU years to process all the files.

Due to memory limitations, only SwiftOrtho and FastOrtho finished the orthology inference on Bac. The results are provided in Table 3. The numbers of (co-)orthologs and in-paralogs inferred by SwiftOrtho and FastOrtho were similar. The number of common orthology relationships between SwiftOrtho and FastOrtho was 861,619,519 (98.2% of SwiftOrtho and 90.6% of FastOrtho). Compared with Euk, SwiftOrtho and FastOrtho had a similar predictive quality on Bac. There are 3 possible explainations for these results. The first is that Euk contains many protein isoforms that cause FastOrtho to overestimate the number of orthologs and in-paralogs. The second is that the gene duplication rate in macteria is lower than that in eukaryotes [44, 45]. For Bac, each gene in 1 species has only a small number of homologs in other species, which makes FastOrtho unlikely to overestimate the number of RBHs. The third is that SwiftOrtho uses double-precision floating-point to store the e-value, which increases the precision of the e-value from 10^−180^ to 10^−308^. This improvement also reduces the possibility that FastOrtho may report false RBHs.

Computational resource use varied: of the programs tested, only SwiftOrtho and FastOrtho finished the orthology inference step. FastOrtho and OrthAgogue did not finish the tests owing to insufficient RAM; OrthoMCL aborted after running out of disk space because it needed >18 TB. The peak RAM usage of SwiftOrtho and FastOrtho was 90.6 and 99.5 GB, respectively. When we used the PyPy interpreter, the peak RAM usage of SwiftOrtho was reduced to 72.1 GB. FastOrtho was ∼1.52 times faster than SwiftOrtho, which ran the tests in the CPython interpreter. When using the PyPy interpreter, SwiftOrtho ran 1.58 times faster than FastOrtho. The memory usage and CPU time are reported in Table S7.

#### Cluster analysis

The clustering numbers of SwiftOrtho and FastOrtho were similar. We compared the APC algorithm and the MCL algorithm, and APC found fewer clusters than MCL. The APC used much less memory and less CPU time than MCL. However, owing to the lack of support for multi-threading and a large number of I/O operations, the real run time of APC is longer than that of MCL.

#### Tests on a low-memory system

Because SwiftOrtho is designed to process large-scale data on low-memory computers, we used it to analyze Bac on a range of computers with different specifications.

The results show that the memory usage of SwiftOrtho is flexible and adapts to the size of the computer’s memory. In the tests, SwiftOrtho finished an orthology analysis of the Bac set on a computer with only 4 GB RAM in a reasonable time (Table S8).

### Comparison with other orthology analysis pipelines

SonicParanoid, OMA, and ProteinOrth are also graph-based methods and have been optimized for large-scale data sets [17, 31, 32]. We compared SwiftOrtho with these tools in both speed and memory usage. The results are presented in Table S10. OMA seems to be the slowest because it uses the Smith-Waterman algorithm to perform all-vs-all alignment. In our tests, OMA took 0.84 CPU hours to align 2 species (4,064 and 4,140 genes) of the Bac set. For the Bac set, OMA needs to perform 3,097,600 species-by-species alignments and the total time will be >2 million CPU hours. SonicParanoid worked well on the Euk and QfO 2011 sets. Compared with SwiftOrtho, SonicParanoid ran faster and required less RAM on small data sets. However, it exited abnormally when applied to the large Bac set. Proteinortho also worked well on the Euk and QfO 2011 sets. When applied to the Bac set, Proteinortho needed to perform 1,547,920 species-by-species proteome alignments. It took Proteinortho 186.5 CPU hours, using DIAMOND, to complete 23,331 (1.5%) alignments; we therefore estimate that Proteinortho will take ∼12,355 CPU hours to finish a full homology search. Because LAST is much faster than DIAMOND, we reran Proteinortho on the Bac set, using LAST for homology search. The CPU time for LAST on the Bac set was 2,368 hours. Although the previous results (Supplementary Table S9) show that LAST is ∼20 times faster than SwiftOrtho, LAST required much more CPU time than SwiftOrtho in the all-vs-all homology search step. We think it is because the species-by-species alignment approach requires >1.5 million I/O operations, which significantly reduces the speed. The CPU utilization of orthology inference and clustering of Proteinortho was very low (<10%) when applied to the Bac set, which led to an exceptionally long real time run (>150 hours). The reason for this exceptionally long run time is because Proteinortho occupied ∼85% of physical memory when applied to large-scale data, which resulted in frequent data exchange between RAM and swap space and greatly reduced the speed. In sum, these results show that SwiftOrtho is a top performer on large-scale data.

## Discussion

We present SwiftOrtho, a new high-performance graph-based homology classification tool. Unlike most tools that only perform orthology inference, SwiftOrtho integrates all the modules necessary for a full orthology analysis, including homology search, orthology inference, and cluster analysis. SwiftOrtho is designed to analyze large-scale genomic data on a normal desktop computer in a reasonable time. In our tests, SwiftOrtho’s homology search module was nearly 30 times faster than BLASTP. The orthology inference module of SwiftOrtho was nearly 500 times faster than OrthoMCL when applied to Euk. When applied to the large-scale data set, Bac, SwiftOrtho was the only program that finished the orthology inference test on a workstation with 32 GB RAM. The cluster module of SwiftOrtho using APC can handle data that are much larger than the available RAM. In our test, APC had comparable recall and accuracy but required considerably less memory than MCL. It should be noted that APC improved the *F*_1_-measure score by increasing recall in most cases. With the help of these optimized modules, SwiftOrtho has successfully finished an orthology analysis of proteins from 1,760 bacterial genomes on a machine with only 4 GB RAM, which makes SwiftOrho usable for large-scale analyses for researchers who may not have access to expensive computational resources. SwiftOrtho is not only fast but also accurate, as shown in the results produced when running on orthobench and QfO [13, 33].

## Potential Implications

In summary, SwiftOrtho is a fast and accurate orthology prediction tool that can analyze a large number of sequences with minimal computational resource use. The installation and configuration of SwiftOrtho is simple and does not require the user to have any experience in database configuration. It is easy to use because the only input required by SwiftOrtho is a FASTA format file of protein sequences with taxonomy information in the header line. SwiftOrtho can be integrated into various common pipelines where fast orthology classification is required such as pan-genome analysis, large-scale phylogenetic tree construction, and other multi-genome analyses. It is specifically suited for microbial community analyses, where a large number of sequences and species are involved.

## Methods

### Algorithms

Here we outline the homology search, orthology inference, and clustering as implemented in SwiftOrtho.

#### Homology search

**Figure 6:**
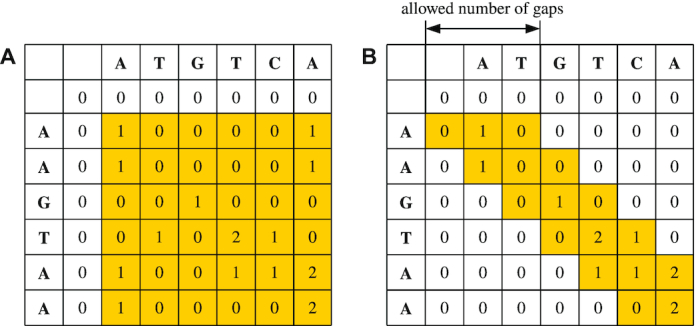
Comparing standard Smith-Waterman with banded Smith-Waterman. A. Similarity score matrix for standard Smith-Waterman. The standard Smith-Waterman algorithm needs to calculate all the entries. B. Similarity score matrix for banded Smith-Waterman. The banded Smith-Waterman algorithm only needs to calculate the entries on and near the diagonal.

SwiftOrtho uses a seed-and-extension algorithm to find homologous gene pairs [46, 47]. At the seed phase, SwiftOrtho finds candidate target sequences that share common *k*-mers with the query sequence. *k*-mer size is an important factor that affects search sensitivity and speed [38, 48]. SwiftOrtho therefore uses long (≥6) *k*-mers to accelerate search speed. At the same time, *k*-mer length is negatively correlated with sensitivity [38]. To compensate for the loss of sensitivity caused by increasing the *k*-mer size, SwiftOrtho uses 2 approaches: non-consecutive *k*-mers and reduced amino acid alphabets. Non-consecutive *k*-mer seeds (known as spaced seeds) were introduced in PatternHunter [19, 49]. The main difference between consecutive seeds and spaced seeds is that the latter allow mismatches in alignment. For example, the spaced seed 101101 allows mismatches at positions 2 and 5. The total number of matched positions in a spaced seed is known as the weight, so the weight of this seed is 4. A consecutive seed can be considered as a special case of spaced seed in which its weight equals its length. Spaced seeds often provide a better sensitivity than consecutive seeds [49, 50]. Several tools such as PatternHunter, Usearch, LAST, and DIAMOND [19, 39–41, 49] have used spaced seed to increase sensitivity. PatternHunter and Usearch allow users to use custom spaced seed. The default spaced seed patterns of SwiftOrtho are 1110100010001011, 11010110111—two spaced seeds with weight of 8—but the user can define their own spaced seeds. Seed patterns were optimized using SpEED [50] and manual inspection. The choice of the spaced seeds and default alphabet are elaborated upon in the Methods section and in the Supplementary Materials sections 2.1 and 3.. At the extension phase, SwiftOrtho uses a variation of the Smith-Waterman algorithm [51], the *k*-banded Smith-Waterman or *k*-SWAT, which only allows for *k* gaps [52]. *k*-SWAT fills a band of cells along the main diagonal of the similarity score matrix (Figure 6B), and the complexity of *k*-SWAT is reduced to *O*[*k* · min(*n, m*)], where *k* is the maximum allowed number of gaps.

Reduced alphabets are used to represent protein sequences using an alternative alphabet that combines several amino acids into a single representative letter, based on common physico-chemical traits [53–55]. Compared with the original alphabet of 20 amino acids, reduced alphabets usually improve sensitivity [56, 57]. At the same time, reduced alphabets also introduce less specific seeds than the original alphabet, reducing the search speed.

#### Orthology inference

The orthology inference step in Fig. 1 shows the algorithm to infer orthologs and in-paralogs from homologs: gene A_1_ in genome A and B_1_ in genome B are considered to be orthologs according to the RBH rule. If the bit score between gene A_1_ and A_2_ in genome A is higher than that between A_1_ and all its orthologs in other genomes, A_1_ and A_2_ are considered in-paralogs in genome A. If A_1_ in genome A and B_1_ in genome B are orthologs, in-paralogs of A_1_ and B_1_ are co-orthologs. Because orthology inference requires many queries, it is better to store the data in a way that facilitates fast querying. First, SwiftOrtho sorts the data and stores it in the file system. Then, it uses binary search to query the sorted data, dramatically reducing memory usage when compared with a relational database management system or a hash table. With the help of this query system, SwiftOrtho can process data that are much larger than the computer memory.

The inferred relationships are treated as the edges of a graph. Each edge is assigned a weight for cluster analysis, where using appropriate edge-weighting metrics can improve the accuracy of cluster analysis. Gibbons et al. [58] compared the performance of several BLAST-based edge-weighting metrics and found that the bit score had the best performance. Therefore, SwiftOrtho uses the normalized bit score as edge-weighting metric. The normalization step takes the same approach as OrthoMCL [20]. For orthologs or co-orthologs, the weight of (co-)ortholog (Fig. 1) A_1_ in genome A and B_1_ in genome B is divided by the average edge-weight of all the (co-)orthologs between genome A and genome B. For in-paralogs, SwiftOrtho identifies a subset S of all in-paralogs in genome A, with each in-paralog A_*x*_-A_*y*_ in subset S, A_*x*_ or A_*y*_ having ≥1 ortholog in another genome. The weight of each in-paralog in genome A is divided by the mean edge-weight of subset S in genome A [20].

#### Clustering orthology relationships into orthologous groups

SwiftOrtho provides 2 methods to cluster orthology relationships into orthologous groups. One is the Markov cluster algorithm (MCL), an unsupervised clustering algorithm based on simulation of flow in graphs [21]. MCL is fast and robust on small networks and has been used by several graph-based tools [19, 59–61]. However, MCL may run out of memory when applied to a large-scale network. To reduce memory usage, we cluster each individual connected component instead of the whole network because there is no flow among components [21]. For large and dense networks a single connected component could still be too large to be loaded into memory.

For large networks, SwiftOrtho uses an APC algorithm [62]. The APC algorithm finds a set of centers in a network, where the centers are the actual data points and are called “exemplars.” To find exemplars, APC needs to maintain 2 matrices: the responsibility matrix *R* and the availability matrix *A*. The element *R_i, k_* in *R* reflects how well suited node *k* is to serve as the exemplar for node *i* while the element *A_i, k_* in *A* reflects how appropriate node *i* is to choose node *k* as its exemplar [62]. APC uses Equation (1) to update *R* and Equation (2) to update *A*, where *i, k, i*′, *k*′ denote the node number and *S$_{i,k^{\prime }}$* denotes the similarity between node *i* and node *k*′.

(1) $$\begin{equation*}
R_{i,k} = S_{i,k} - \mathrm{max}_{k^{\prime } \ne k} \lbrace A_{i, k^{\prime }} + S_{i, k^{\prime }}\rbrace,
\end{equation*}$$

(2) $$\begin{equation*}
A_{i, k} = \left\lbrace \begin{array}{@{}l@{\quad }l@{}}\mathrm{min} \lbrace 0, R_{k, k} + \sum _{i^{\prime }\not\in \lbrace i, k\rbrace } \mathrm{max} \lbrace 0, R_{i^{\prime }, k} \rbrace , & \text{if}\ i \ne k \\
\sum _{i^{\prime }\ne k} \mathrm{max} \lbrace 0, R_{i^{\prime }, k} \rbrace , & \text{if}\ i = k \end{array}\right.
\end{equation*}$$

The node *k* that maximizes *A_i, k_* + *R_i, k_* is the exemplar of node *i*, and each node *i* is assigned to its nearest exemplar. APC can update each element of matrix *R* and *A* one by one, so it is unnecessary to keep the whole matrix of *R* and *A* in memory. Generally, the time complexity of APC is O(*N*^2^ · *T*), where *N* is the number of nodes and *T* is the number of iterations [62]. In this case, the time complexity is *O*(*E* · *T*), where *E* stands for edges, which is the number of orthology relationships, and *T* is the number of iterations. We implemented APC in Python, using Numba [63] to accelerate the numeric-intensive calculation parts.

## Availability of Source Code and Requirements

Project Name: SwiftOrtho

Project Home Page: https://github.com/Rinoahu/SwiftOrtho

Operating System(s): SwiftOrtho was tested on GNU/Linux distribution Ubuntu 16.04 64-bit, but we expect SwitOrtho to work on most *nix systems

Programming Language: Python

Other Requirements: Python 2.7, Python 3.7, PyPy2.7 v7.0 or higher

License: GPLv3

RRID:SCR_017122

## Availability of Supporting Data and Materials

The data sets supporting the results of this article are available in the GigaDB repository [64].

## Additional Files

Supplementary Material S1. Further details on the methodolgy.

giz118_GIGA-D-19-00043_Original_Submission

giz118_GIGA-D-19-00043_Revision_1

giz118_GIGA-D-19-00043_Revision_2

giz118_GIGA-D-19-00043_Revision_3

giz118_Response_to_Reviewer_Comments_Original_Submission

giz118_Response_to_Reviewer_Comments_Revision_1

giz118_Response_to_Reviewer_Comments_Revision_2

giz118_Reviewer_1_Report_Original_Submission Marnix Medema -- 3/14/2019 Reviewed

giz118_Reviewer_1_Report_Revision_1 Marnix Medema -- 6/27/2019 Reviewed

giz118_Reviewer_2_Report_Original_Submission Marcus Lechner, Ph.D. -- 3/22/2019 Reviewed

giz118_Reviewer_2_Report_Revision_1 Marcus Lechner, Ph.D. -- 7/10/2019 Reviewed

giz118_Reviewer_3_Report_Original_Submission Robert Davey -- 3/29/2019 Reviewed

giz118_Reviewer_3_Report_Revision_1 Robert Davey -- 7/16/2019 Reviewed

giz118_Supplemental_File

## Abbreviations

APC: Affinity Propagation Clustering; BLAST: Basic Local Alignment Search Tool; BLAT: BLAST-Like Alignment Tool; COG: Clusters of Orthologous Groups; CPU: central processing unit; I/O: input/output; MCL: Markov clustering; RBH: reciprocal best hit; OMA: Orthologous Matrix; QFO: Quest for Orthologs; RAM: random access memory.

## Competing Interests

The authors declare that they have no competing interests.

## Funding

This study has been funded, in part, by National Science Foundation award ABI 1458359. The funders had no role in the design of the study and collection, analysis, and interpretation of data and in writing the manuscript.

## Author information

I.F. is an associate professor at the Department of Veterinary Microbiology and Preventive Medicine at Iowa State University. He is also the chair of the Interdepartmental Bioinformatics and Computational Biology graduate program. X.H. was a postodcotoral associate at Iowa State University at the time of this work, and currently is a postdoctoral associate at the Gianforte School of Computing, Montana State University.

## Author’s Contributions

Both authors conceived the study. X.H. wrote the software and performed the analysis. Both authors wrote the manuscript.
