# Supplementary material for: SwiftOrtho: A fast, memory-efficient, multiple genome orthology classifier
Source: Gigascience. 2019 Oct 24;8(10):giz118. doi: 10.1093/gigascience/giz118 (PMC6812468; doi:10.1093/gigascience/giz118)
Supplement: giz118_GIGA-D-19-00043_Revision_1 [file giz118_giga-d-19-00043_revision_1.pdf]

GigaScience
SwiftOrtho: a Fast, Memory-Efficient, Multiple Genome Orthology Classifier
--Manuscript Draft--

**Manuscript Number:**	GIGA-D-19-00043R1
**Full Title:**	SwiftOrtho: a Fast, Memory-Efficient, Multiple Genome Orthology Classifier
**Article Type:**	Technical Note
**Funding Information:**	Directorate for Biological Sciences (1854685) — Dr. Iddo Friedberg
**Abstract:**	Background: Gene homology type classification is a requisite for many types of genome analyses, including comparative genomics, phylogenetics, and protein function annotation. A large variety of tools have been developed to perform homology classification across genomes of different species. However, when applied to large genomic datasets, these tools require high memory and CPU usage,typically available only in computational clusters.<br><br>Findings: Here we present a new graph-based orthology analysis tool,SwiftOrtho, which is optimized for speed and memory usage when applied to large-scale data. SwiftOrtho uses long k-mers to speed up homology search, while using a reduced amino acid alphabet and spaced seeds to compensate for the loss of sensitivity due to long k-mers. In addition, it uses an Affinity Propagation algorithm to reduce the memory usage when clustering large-scale orthology relationships into orthologous groups. In our tests, SwiftOrtho is the only tool that completed orthology analysis of proteins from 1,760 bacterial genomes on computer with only 4GB RAM. Using various standard orthology datasets, we also show that SwiftOrtho has a high accuracy.<br><br>Conclusion: SwiftOrtho enables the accurate comparative genomic analyses of thousands of genomes using low memory computers.Availability:https://github.com/Rinoahu/SwiftOrtho
**Corresponding Author:**	Iddo Friedberg<br>Iowa State University<br>Ames, Iowa UNITED STATES
**Corresponding Author Secondary Information:**	
**Corresponding Author's Institution:**	Iowa State University
**Corresponding Author's Secondary Institution:**	
**First Author:**	Xiao Hu
**First Author Secondary Information:**	
**Order of Authors:**	Xiao Hu
	Iddo Friedberg
**Order of Authors Secondary Information:**	
**Response to Reviewers:**	Dear Dr. Zauner,<br><br>Enclosed please find our revised manuscript. We would like to thank the reviewers and yourself for the large amount of time and effort that were spent reading and commenting on the manuscript. We are grateful for this effort, and we have addressed all comments in detail. We would like to stress that SwiftOrtho's strength lies both in its modular versatility, and in its low consumption of computational resources, making it especially suitable for low- and medium budget labs, but is easy and accurate enough to be used universally.<br><br>We have upgraded the software to Python 3, and rewrote the code to conform with PEP-8. We have also compared with all the other tools requested, and some others,

Powered by Editorial Manager® and ProduXion Manager® from Aries Systems Corporation

	including DIAMOND, Usearch, TOPAZ, LAST and BLAT.<br><br>We are happy to include the paper in the Technical Notes section. We have registered SwiftOrtho in SciCrunch.org, and added the Software Availability section, and the availability of supporting source code and requirements.<br><br>Attached are the reviewers' requests, and our detailed responses are in italics. We are looking forward to your feedback.<br><br>Sincerely,<br><br>Iddo Friedberg
**Additional Information:**	
**Question**	**Response**
Are you submitting this manuscript to a special series or article collection?	No
**Experimental design and statistics**<br><br>Full details of the experimental design and statistical methods used should be given in the Methods section, as detailed in our Minimum Standards Reporting Checklist. Information essential to interpreting the data presented should be made available in the figure legends.<br><br>Have you included all the information requested in your manuscript?	Yes
**Resources**<br><br>A description of all resources used, including antibodies, cell lines, animals and software tools, with enough information to allow them to be uniquely identified, should be included in the Methods section. Authors are strongly encouraged to cite Research Resource Identifiers (RRIDs) for antibodies, model organisms and tools, where possible.<br><br>Have you included the information requested as detailed in our Minimum Standards Reporting Checklist?	Yes
**Availability of data and materials**	Yes

Powered by Editorial Manager® and ProduXion Manager® from Aries Systems Corporation

All datasets and code on which the conclusions of the paper rely must be either included in your submission or deposited in publicly available repositories (where available and ethically appropriate), referencing such data using a unique identifier in the references and in the "Availability of Data and Materials" section of your manuscript.<br><br>Have you have met the above requirement as detailed in our Minimum Standards Reporting Checklist?	

Manuscript

Click here to access/download;Manuscript;SwiftOrtho_note_2_gigascience_re

Click here to view linked References

SwiftOrtho: a Fast, Memory-Efficient, Multiple Genome Orthology Classifier

Xiao Hu
and Iddo Friedberg*

*Correspondence: idoerg@iastate.edu
Department of Veterinary Microbiology and Preventive Medicine, College of Veterinary Medicine, Iowa State University, 2118 Vet Med, Ames, IA 50011, USA
Full list of author information is available at the end of the article

Abstract

Background: Gene homology type classification is a requisite for many types of genome analyses, including comparative genomics, phylogenetics, and protein function annotation. A large variety of tools have been developed to perform homology classification across genomes of different species. However, when applied to large genomic datasets, these tools require high memory and CPU usage, typically available only in computational clusters.

Findings: Here we present a new graph-based orthology analysis tool, SwiftOrtho, which is optimized for speed and memory usage when applied to large-scale data. SwiftOrtho uses long k-mers to speed up homology search, while using a reduced amino acid alphabet and spaced seeds to compensate for the loss of sensitivity due to long k-mers. In addition, it uses an Affinity Propagation algorithm to reduce the memory usage when clustering large-scale orthology relationships into orthologous groups. In our tests, SwiftOrtho is the only tool that completed orthology analysis of proteins from 1,760 bacterial genomes on a computer with only 4GB RAM. Using various standard orthology datasets, we also show that SwiftOrtho has a high accuracy.

Conclusion: SwiftOrtho enables the accurate comparative genomic analyses of thousands of genomes using low memory computers.

Availability: https://github.com/Rinoahu/SwiftOrtho

Abbreviations: RBH: Reciprocal Best Hit; **MCL:** Markov Clustering algorithm; **APC:** Affinity Propagation Clustering.

Background

Gene homology type classification consists of identifying paralogs and orthologs across species. Orthologs are genes that evolved from a common ancestral gene following speciation, while paralogs are genes that are homologous due to duplication. Paralogs can be further classified into in-paralogs, which evolved via gene duplication before the speciation event, and out-paralogs, which evolved via gene duplication after the speciation event [1]. Classifying orthologs and paralogs across species is an important problem, as the evolutionary history of genes has implications for our understanding of gene function and evolution.

While the proper inference of homology type involves tracing gene history using phylogenetic trees [2], several proxy methods have been developed over the years. The most common method to infer orthologs by proxy is Reciprocal Best Hit or RBH [3, 4]. Briefly, RBH states the following: when two proteins that are encoded by two genes, each in a different genome, find each other as the best scoring match among all homologs, they are considered to be orthologs [3, 4].

Inparanoid extends the RBH orthology relationship to include both orthologs and in-paralogs. Specifically, Inparanoid uses RBH to identify orthologs between two species. The genes in the two species are classified as in-paralogs if they are more similar to the corresponding ortholog than to any gene in the other species [5–7]. The concept of orthologous pairs between two species can be extended to an *ortholog group*, whcihc is a set of genes that are hypothesized to have descended from a common ancestor [7]. Several methods have been developed to identify ortholog groups across multiple species typically classified as either tree-based or graph-based methods. Tree-based methods construct a gene tree from an alignment of homologous sequences in different species and infer orthology relationships by reconciling the gene tree with its corresponding species tree [2, 8, 9], and can infer a correct orthology relationship if the correct gene tree and species tree are provided [10]. The chief limiting factor of tree-based methods is the accuracy of the given gene tree and species tree. Erroneous trees lead to incorrect ortholog and in-paralog assignments [9–11]. Tree-based methods are also computationally expensive which limits the ability to apply them to large number of species [10, 12–14]. Graph-based methods infer orthologs and in-paralogs from homologs and then use different strategies to cluster them into orthologous groups [9, 12, 13] (Figure 1). The Clusters of Orthologous Groups (COG) database detects triangles of RBHs in three different species and merges the triangles with a common side [15]. Orthologous Matrix (OMA) clusters RBHs in orthologous groups by finding maximum

weight cliques from the similarity graph [16, 17]. MultiParanoid is an extension of Inparanoid, which uses InParanoid to detect triangle orthologs and in-paralogs in three different species as seeds and then merges the seeds into larger groups [18]. OrthoMCL also uses InParanoid to detect orthologs, co-orthologs, and in-paralogs between two species [19, 20] and then uses Markov Clustering (MCL) [21] to cluster these relationships into orthologous groups, where the co-orthologs are two or more genes in one species that are orthologous to one or more genes in another species due to a gene duplication event [1, 22]. In addition, there is a hybrid method that combines both graph-based and tree-based methods [12, 23–26]. The hybrid method first perform all-*vs*-all sequence alignment, then constructs gene families by the sequence similarity or conserved gene neighborhood. EnsEMBL first uses RBH to find the gene families, then constructs a phylogenetic gene tree for each gene family [24]. Finally, each gene tree is reconciled with the species tree to infer paralogs and orthologs.

In theory, graph-based methods are less accurate than tree-based methods, as the former identify orthologs and in-paralogs using proxy methods rather than directly inferring homology type from gene and species evolutionary history. In practice, graph-based methods are comparably accurate to tree-based methods [10, 11, 27]. Moreover, a comparison of several methods found that tree-based methods had even a worse performance than graph-based methods on large datasets [11]. One study compared several common methods, including simple RBH, graph-based, tree-based, and hybrid methods, and found that tree-based methods of Inparanoid and OrthoMCL exhibit the best balance of sensitivity and specificity [28]. Several studies have also shown that graph-based methods find a better trade-off between specificity and sensitivity than tree-based methods [11, 28, 29]. For these reasons, graph-based methods are generally preferred for analyzing large-scale data sets. OrthoMCL and

InParanoid have been applied to analyze hundreds of genomes, however, they require considerable computational resources that may not be readily available [20, 30].

Recently, several graph-based tools, such as SonicParanoid, OMA, and ProteinOrtho [17, 31, 32] have been developed to speed up orthology analysis on large-scale data sets. However, these tools require high performance computers to analyze large-scale data.

Here we present SwiftOrtho, a fast method for orthology classification that requires minimal use of computational resources, especially memory. SwiftOrtho uses a seed-and-extension method to speed up homology search, a binary search method and RBH rule to infer orthologs and in-paralogs, and the Affinity Propagation algorithm to reduce memory usage in cluster analysis. We compare SwiftOrtho with several existing graph-based tools using the gold standard dataset Orthobench [13], and the Quest for Orthologs service [33]. Using both benchmarks, we show that SwiftOrtho provides a high accuracy with lower CPU and memory usage than other graph-based methods. SwiftOrtho is the only tool that completed an orthology analysis of 1,760 bacterial genomes on very a low-memory computer. With the growing number of genomes, especially microbial genomes, we see SwiftOrtho to be a tool of choice for a fast and accurate ortholog classification, while requiring low computational resources, as are found in conventional desktop or laptop computers.

Methods

Algorithms

Here we outline the homology search, orthology inference, and clustering as implemented in SwiftOrtho.

Homology Search

SwiftOrtho employs a seed-and-extension algorithm to find homologous gene pairs [34, 35]. At the seed phase, SwiftOrtho finds candidate target sequences that share common k-mers with the query sequence. k-mer size is an important fac-

tor that affects search sensitivity and speed [36, 37]. SwiftOrtho therefore uses long (≥ 6) k-mers to accelerate search speed. At the same time, k-mer length is negatively correlated with sensitivity [36]. To compensate for the loss of sensitivity caused by increasing the k-mer size, SwiftOrtho uses two approaches: non-consecutive k-mers and reduced amino-acid alphabets. Non-consecutive k-mer seeds (known as spaced seeds), were introduced in PatternHunter [19, 38]. The main difference between consecutive seeds and spaced seeds is that the latter allow mismatches in alignment. For example, the spaced seed `101101` allows mismatches at positions 2 and 5. The total number of matched positions in a spaced seed is known as the weight, so the weight of this seed is 4. A consecutive seed can be considered as a special case of spaced seed in which its weight equal its length. Spaced seeds often provide a better sensitivity than consecutive seeds [38, 39]. Several tools such as PatternHunter, Usearch, LAST, and DIAMOND [19, 38, 40–42] have used spaced seed to increase sensitivity. PatternHunter and Usearch allow users to use custom spaced seed. The default spaced seed patterns of SwiftOrtho are `1110100010001011`, `11010110111` –two spaced seeds with weight of 8– but the user may define their own spaced seeds. Seed patterns were optimized using SpEED [39] and manual inspection. The choice of the spaced seeds and default alphabet are elaborated upon in the Methods section and in the Supplementary Materials. At the extension phase, SwiftOrtho uses a variation of the Smith-Waterman algorithm [43], the k-banded Smith-Waterman or k-SWAT, which only allows for k gaps [44]. k-SWAT fills a band of cells along the main diagonal of the similarity score matrix (Figure 2B), and the complexity of k-swat is reduced to $O(k \cdot min(n, m))$, where k is the maximum allowed number of gaps. Reduced alphabets are used to represent protein sequences using an alternative alphabet that combines several amino acids into a single representative letter, based on common physico-chemical traits [45–47]. Compared with the original alphabet of 20 amino acids, reduced alphabets usually improve sensitivity [48, 49]. At

the same time, reduced alphabets also introduce less specific seeds than the original alphabet, which reduces the search speed.

Orthology Inference

The orthology inference step in Figure 1 shows the algorithm to infer orthologs and in-paralogs from homologs: gene A_1 in genome A and B_1 in genome B are considered to be orthologs according to the RBH rule; If the bit score between gene A_1 and A_2 in genome A is higher than that between A_1 and all its orthologs in other genomes, A_1 and A_2 are considered in-paralogs in genome A; If A_1 in genome A and B_1 in genome B are orthologs, in-paralogs of A_1 and B_1 are co-orthologs. Since orthology inference requires many queries it is better to store the data in a way that facilitates fast querying. First, SwiftOrtho sorts the data and store it on hard drive. Then, it uses binary search to query the sorted data, which significantly reduces memory usage when compared with a relational database management system or a hash table. With the help of this query system, SwiftOrtho can process data that are much larger than the computer memory.

After inferring orthology, the inferred orthology relationships are treated as the edges of a graph. Each edge is assigned a weight for cluster analysis. Appropriate edge-weighting metrics can improve the accuracy of cluster analysis. Gibbons [50], compared the performance of several BLAST-based edge-weighting metrics and found that the bit score has the best performance. Therefore, SwiftOrtho uses the normalized bit score as edge-weighting metric. The normalization step takes the same approach as OrthoMCL [20]. For orthologs or co-orthologs, the weight of (co-)ortholog (Figure 1) A_1 in genome A and B_1 in genome B is divided by the average edge-weight of all the (co-)orthologs between genome A and genome B. For in-paralogs, SwiftOrtho identifies a subset S of all in-paralogs in genome A, with each in-paralog A_x-A_y in subset S, A_x or A_y having at least one ortholog in

another genome. The weight of each in-paralog in genome A is divided by the mean edge-weight of subset S in genome A [20].

Clustering Orthology Relationships into Orthologous Groups

SwiftOrtho provides two methods to cluster orthology relationships into orthologous groups. One is the Markov Cluster algorithm (MCL), an unsupervised clustering algorithm based on simulation of flow in graphs [21]. MCL is fast and robust on small networks and has been used by several graph-based tools [19, 51–53]. However, MCL may run out of memory when applied to a large-scale network. To reduce memory usage, we cluster each individual connected component instead of the whole network because there is no flow among components [21]. For large and dense networks a single connected component could still be too large to be loaded into memory.

For the large networks, SwiftOrtho uses an Affinity Propagation Clustering algorithm (APC) [54]. The APC algorithm finds a set of centers in a network, where the centers are the actual data points and are called "exemplars". To find exemplars, APC needs to maintain two matrices: the responsibility matrix R, and the availability matrix A. The element $R_{i,k}$ in R reflects how well-suited node k is to serve as the exemplar for node i while the element $A_{i,k}$ in A reflects how appropriate node i to choose node k as its exemplar [54]. APC uses Equation 1 to update R, and Equation 2 to update A, where i, k, i', k' denote the node number, and $S_{i,k'}$ denotes the similarity between node i and node k'.

$$R_{i,k} = S_{i,k} - max_{k' \neq k}\{A_{i,k'} + S_{i,k'}\} \tag{1}$$

$$A_{i,k} = \begin{cases} min\{0, R_{k,k} + \sum_{i' \notin \{i,k\}} max\{0, R_{i',k}\}, & \text{if } i \neq k \\ \sum_{i' \neq k} max\{0, R_{i',k}\}, & \text{if } i = k \end{cases} \tag{2}$$

The node k that maximizes $A_{i,k} + R_{i,k}$ is the exemplar of node i, and each node i is assigned to its nearest exemplar. APC can update each element of matrix R and A one by one, so, it is unnecessary to keep the whole matrix of R and A in memory. Generally, the time complexity of APC is $\mathrm{O}(N^2 \cdot T)$ where N is number of nodes and T is number of iterations [54]. In this case, the time complexity is $O(E \cdot T)$, where E stands for edges which is number of orthology relationships and T is number of iterations. We implemented APC in Python, using Numba [55] to accelerate the numeric-intensive calculation parts.

Application of SwiftOrtho

Data Sets

We applied SwiftOrtho to three data sets to evaluate its predictive quality and performance:

1 The *Euk* set was used to evaluate the quality of predicted orthologous groups. This set contains 420,415 protein sequences from 12 eukaryotic species, including *Caenorhabditis elegans, Drosophila melanogaster, Ciona intestinalis, Danio rerio, Tetraodon nigroviridis, Gallus gallus, Monodelphis domestica, Mus musculus, Rattus norvegicus, Canis familiaris, Pan troglodytes* and *Homo sapiens*. The protein sequences for these genes were downloaded from EMBL v65 [56].

2 The *QfO 2011* set was used to evaluate the quality of predicted orthology relationships. This set was the reference proteome dataset (2011) of The Quest for Orthologs[33], which contains 754,149 protein sequences of 66 species.

3 The large *Bac* set was used to evaluate performance, including CPU time, real time and RAM usage. This set includes 5,950,817 protein sequences from 1,760 bacterial species. The protein sequences were downloaded from GenBank [57]. For a full list, see the additional file 1.

We also compared SwiftOrtho with several existing orthology analysis tools for predictive quality and performance. The methods compared were: OrthoMCL(v2.0), FastOrtho, OrthAgogue, and OrthoFinder.

Orthology Analysis Pipeline

The pipeline for all the tools follows the standard steps of graph-based orthology prediction, (1) all-*vs*-all homology search, (2) orthology inference, and (3) cluster analysis.

Homology Search

SwiftOrtho used its built-in module to perform all-*vs*-all homology search. For all the three sets, the E-value was set 10^{-5}. The amino acid alphabet was set to the regular 20 amino acids for the three sets. The spaced seed parameter was set to `1011111,11111` for the `Euk`, `11111111` for the *QfO 2011*, and `111111` for *Bac*.

OrthoMCL, FastOrtho, OrthAgogue, and OrthoFinder use BLASTP (v2.2.27+) [58] to perform all-*vs*-all homology search. The first three tools require the user to do this manually. To compare the methods, the -e (e-value), -v (number of database sequences to show one-line descriptions), and -b (number of database sequence to show alignments) parameters of BLASTP were set to 10^{-5}, 1,000,000, and, 1,000,000 for OrthoMCL, FastOrtho, and OrthAgogue. The OrthoFinder calls BLASTP, and the E-value of BLASTP have been set to 10^{-3}.

Orthology Inference

SwiftOrtho, OrthoMCL, FastOrtho, OrthAgogue, and OrthoFinder were applied to perform orthology inference on the homologs. The first four tools are able to identify (co-)orthologs and in-paralogs, and the coverage (fraction of aligned regions) was set to 50%, while other parameters were set to their default values, see Supplementary Materials for full details. FastOrtho does not report (co-)orthologs and in-paralogs directly. However, the relevant information is stored in an intermediate file, from

which we have extracted that information. Orthofinder does not report orthology relationships.

Cluster Analysis

All the tools in this study use MCL [21] for clustering. To control the granularity of the clustering, MCL performs an inflation operation set by the *-I* option [21, 59]. In this study, *-I* was set to 1.5. To take advantage of multiprocessor capabilities, we set the thread number of MCL to 12. SwiftOrtho has an alternative clustering algorithm APC, which we have also applied to *Euk* and*Bac*.

Evaluation of Prediction Quality

Evaluation of Predicted Orthologous Group

The OrthoBench set was used to evaluate the quality of predicted orthologous groups in *Bac*. This set contains 70 manually curated orthologous groups of the 12 species from *Bac* and has been used as a high quality gold standard benchmark set for orthologous group prediction [13]. In this study, we used OrthoBench v2 (Supplementary Table S1). Each manually curated group of OrthoBench v2 set finds the best match in the predicted orthologous groups, where the best match means that the number of genes shared between manually curated and predicted orthologs is maximized, and the method to calculate precision and recall is shown in Supplementary Figure S1.

Evaluation of Predicted Orthology Relationships

The *Quest of Orthologs* web-based service (QfO) was employed to evaluate the quality of the orthology relationships predicted from the *QfO 2011* set[33]. QfO service evaluates the predictive quality by performing four phylogeny-based tests of *Species Tree Discordance Benchmark*, *Generalized Species Tree Discordance Benchmark*, *Agreement with Reference Gene Phylogenies: SwissTree*, and *Agreement with Reference Gene Phylogenies: TreeFam-A*, and two function-based tests of *Gene Ontology*

conservation test and *Enzyme Classification conservation test* [33]. We also applied two more orthology prediction tools, SonicParanoid[31] and InParanoid (v4.1)[5], on the *QfO 2011* set and used their results as control because InParanoid has best performance among the results from QfO service website and SonicParanoid is a fast implementation of InParanoid. The pairwise orthology relationships were extracted from the predicted orthologous groups of all the tools, including SonicParanoid and InParanoid, and then submitted to the QfO web-service for further evaluation.

Hardware

Unless specified otherwise, all tests were run on the Condo cluster of Iowa State University with Intel Xeon E5-2640 v3 at 2.60GHz, 128GB RAM, 28TB free disk. The Linux command `time -v` was used to track CPU and peak memory usage.

Findings

We compared the orthology analysis performance of SwiftOrtho, OrthoMCL, FastOrtho, OrthAgogue, and OrthFinder using *Euk*, *QfO 2011*, and *Bac*. The orthology analysis consists of homology search, orthology inference, and cluster analysis.

Orthology Analysis on *Euk*

The results of orthology analysis on *Euk* are summarized in Table 1, and are elaborated upon below.

<table>
<tr><th colspan="2"></th><th colspan="2">SwiftOrtho</th><th>OrthoMCL</th><th>FastOrtho</th><th>OrthAgogue</th><th>OrthoFinder</th></tr>
<tr><td rowspan="3">Homology Search</td><td>Method</td><td colspan="2">SO built-in</td><td colspan="4">BLASTP</td></tr>
<tr><td>Hits</td><td colspan="2">162,695,330</td><td colspan="3">947,203,546</td><td>654,792,861</td></tr>
<tr><td>Uniq Hits</td><td colspan="2">162,695,330</td><td colspan="3">297,107,872</td><td>266,104,611</td></tr>
<tr><td colspan="8"></td></tr>
<tr><td rowspan="2">Orthology Inference</td><td>(Co-)orthologs</td><td colspan="2">1,422,920</td><td>8,279,424</td><td>3,297,613</td><td>1,265,553</td><td>N/A</td></tr>
<tr><td>In-paralogs</td><td colspan="2">631,033</td><td>2,517,166</td><td>2,546,296</td><td>759,989</td><td>N/A</td></tr>
<tr><td colspan="8"></td></tr>
<tr><td rowspan="2">Clustering</td><td>Algorithm</td><td>MCL</td><td>APC</td><td colspan="4">MCL</td></tr>
<tr><td>Orthologous Groups</td><td>44,551</td><td>38,748</td><td>36,901</td><td>40,943</td><td>51,297</td><td>19,904</td></tr>
</table>

Table 1 Comparative orthology analysis on the *Euk* set. N/A: not available, SO: SwiftOrtho, MCL: Markov Clustering, APC: Affinity Propagation Cluster.

Homology Search

The homology search results show that BLASTP detected the largest number of homologs (947,203,546). SwiftOrtho found 57.5% of the homologs detected by BLASTP but was 38.7 times faster than BLASTP. SwiftOrtho used longer k-mers, which reduced both specific and non-specific seed extension. The longer k-mers cause seed-and-extension methods to ignore sequences with low similarity. According to the RBH rule, orthologs should have higher similarity than non-orthologs, so, the decrease in homolgs of SwiftOrtho does not significantly affect the next orthology inference. We compared RBHs inferred from homologs detected by BLASTP and SwiftOrtho, and the numbers of RBHs for BLASTP and SwiftOrtho are 899,473 and 957,387, respectively. Identical RBHs are 767,884 (85.37% of BLASTP). These results shows that although SwiftOrtho found fewer homologs than BLASTP, it does not significantly reduce the number of RBHs. The following results in Figure 4 also show that there is no significant difference between SwiftOrtho and BLASTP in orthologous groups prediction. Homology searches against a large number of protein sequences are a major bottleneck in bioinformatics pipelines. For that reason, many tools have been developed to speed up this process including, among others, BLAT, Usearch, LAST, DIAMOND, and Topaz [36, 40–42, 60]. All these tools use longer k-mers than BLASTP to speed up performance. We also compared SwiftOrtho with them in speed and sensitivity, (Supplementary Table S9). Because BLASTP is widely considered the gold standard for comparing protein sequences, we use its results as the benchmark to evaluate the sensitivity of other homology search tools. We found that Usearch and LAST to be the fastest, however, they only found 0.88% and 2.97% hits of BLASTP, respectively. Topaz and BLAT used the most CPU time, but found only 33.48% and 28.34% hits of BLASTP, respectively. SwiftOrtho and DIAMOND (more sensitive mode) have the highest sensitivity and found 52.72% and 58.30% hits of BLASTP in a moderate amount of time, respec-

tively. These results show that SwiftOrtho has a good trade-off between speed and sensitivity.

Orthology Inference

OrthoMCL and FastOrtho found more orthology relationships than SwiftOrtho and OrthAgogue. This is because OrthoMCL and FastOrtho use the negative log ratio of the e-value as the edge-weighting metric. The BLASTP program rounds E-value $< 10^{-180}$ to 0. Consequently, for homolgs with an e-value $< 10^{-180}$, OrthoMCL and FastOrtho treat them as the RBHs, overestimating the number of orthologs. An example showing the OrthoMCL and FastOrtho overestimation can be found in Table S4.

Use of Computational Resources

OrthoMCL v2.0 used the most CPU time and real time because of the required I/O operations. The RAM usage of OrthoMCL was 3.45GB, while the generated intermediate file occupied >19 TB disk space. OrthAgogue was the most efficient in real time, because of its ability to exploit a multi-core processor. However, the RAM usage of OrthAgogue was more than 100GB which exceeds that of most workstations. The orthology inference module of FastOrtho was the most memory-efficient among all the tools and was also fast. SwiftOrtho was the most CPU time efficient, although its real time was twice as that of OrthAgogue. Because the orthology inference module of SwiftOrtho was written in pure Python, we retested it by using the PyPy interpreter, an alternate implementation of Python [61]. When running with PyPy the real run time of SwiftOrtho was close to that of OrthAgogue (Table S5)

Cluster Analysis

OrthoFinder identified the smallest number of orthologous groups. Other tools identified many more orthologous groups than OrthoFinder, ranging from 36,901 to 51,297. The APC algorithm found fewer clusters than the MCL algorithm.

Evaluation of Predicted Orthologous Groups

The quality of predicted orthologous groups is shown in Figure 3. OrthoFinder has the best recall, while SwiftOrtho and OrthAgogue have top precision values but lower recall values than other tools. Since SwiftOrtho and OrthAgogue use a more stringent standard to perform orthology inference, this strategy often increases precision but decreases recall [11, 28, 29].

Because SwiftOrtho uses its built-in homology search module and its recall is lower than BLASTP's, it may reduce the recall of orthologous groups. To address this problem, we made two replacements. We replaced SwiftOrtho's homology module with BLASTP for SwiftOrtho and replaced BLASTP with SwiftOrtho's homology module for OrthoMCL, FastOrtho, OrthAgogue, and OrthoFinder. We then reran the orthology analysis on *Euk*. The results show that for most tools, replacing BLASTP with SwiftOrtho's built-in homology search module does not significantly reduce the recall (Figure 4). The difference in recall between using SwiftOrtho's homology search and using BLASTP is less than 4% except for OrthoMCL and FastOrtho. The recall for OrthoMCL and FastOrtho decreased by 8% and 7%, respectively. The most likely reason is that the E-value of SwiftOrtho's homology search module is more precise than that of BLASTP, which reduces the false RBHs as mentioned above. These results show that SwiftOrtho's homology search module is a reliable and fast alternative to BLASTP.

Since SwiftOrtho uses an APC clustering algorithm, we ran SwiftOrtho with MCL and APC on the same data. The results (Figure 5) show that performance of APC is very close to that of MCL. APC improves the recall of most tools (Figure 5).

These results show that APC has the similar performance as the MCL algorithm and is a reliable alternative to MCL.

Orthology Analysis on *QfO 2011*

The results of the orthology analysis on *QfO 2011* are shown in Table 2 and elaborated below.

		**SwiftOrtho**	**OrthoMCL**	**FastOrtho**	**OrthAgogue**	**OrthoFinder**
**Homology Search**	Method	SO built-in	BLASTP			
	Hits	183,883,417	642,372,369			935,579,809
	Uniq Hits	183,883,417	317,333,885			462,876,579
**Orthology Inference**	(Co-)orthologs	2,209,243	3,743,779	2,588,851	2,716,128	N/A
	In-paralogs	6,929,058	11,427,118	13,649,582	13,694,208	N/A
**Clustering**	Algorithm	MCL				
	Orthologous Groups	60,418	50,970	55,530	50,203	166,217

Table 2 Comparative orthology analysis on the Quest for Orthologs reference proteome 2011 dataset. SO: SwiftOrtho; MCL: Markov Clustering; APC: Affinity Propagation Cluster; N/A: not available.

Homology Search

SwiftOrtho found 183,883,417 unique hits while BLASTP found 462,876,579 unique hits. However, SwiftOrtho is about 163 times faster than BLASTP.

Orthology Inference

OrthoMCL found many more orthologs and co-orthologs than the other tools. SwiftOrtho found fewer in-paralogs than other available tools. The CPU time of SwiftOrtho is the least of all tools. When using the PyPy interpreter, the real time of SwiftOrtho is also close to that of the fastest one, OrthAgogue (Supplementary Table S6).

Cluster Analysis

Overall, the clustering numbers of SwiftOrtho, OrthoMCL, FastOrtho, and OrthAgogue are similar. However, the number of clusters found by OrthoFinder is three times that of other tools, and the next evaluation also shows that OrthoFinder performed poorly on *QfO 2011*.

Evaluation of Predicted Ortholog Relationships

The evaluation shows that the performance of SwiftOrtho is close to that of Inparanoid (Figure 6). In some tests (Figure 6, D-E), SwiftOrtho outperformed Inparanoid. SwiftOrtho had the best performance in the Generalized Species Tree Discordance Benchmark and Agreement with Reference Gene Phylogenies: TreeFam-A tests. In the Species Tree Discordance Benchmark, SwiftOrtho had the minimum Robinson-Foulds distance. In the Enzyme Classification (EC) conservation test, SwiftOrtho had the maximum Schlicker similarity. These two metrics reflect the accuracy of the algorithm, and the results show that SwiftOrtho has an overall higher accuracy than the other tools. At the same time, the recall of SwiftOrtho was lower in some of the QfO tests, the main reason is that SwiftOrtho uses an stringent metric system to identify orthology relationships.

Orthology Analysis On *Bac*

The results of orthology analysis on *Bac* are summarized in Table 3.

		**SwiftOrtho**		**OrthoMCL**	**FastOrtho**	**OrthAgogue**	**OrthoFinder**
**Homology Search**	Method	SO built-in					N/A
	Hits	8,478,732,753					N/A
	Uniq Hits	8,478,732,753					N/A
**Orthology Inference**	(Co-)orthologs	876,766,940		N/A	950,683,849	N/A	N/A
	In-paralogs	622,292		N/A	663,052	N/A	N/A
**Clustering**	Algorithm	MCL	APC	MCL			
	Orthologous Groups	240,162	167,355	N/A	242,816	N/A	N/A

Table 3 Comparative orthology analysis on the *Bac* set. SO: SwiftOrtho; MCL: Markov Clustering; APC: Affinity Propagation Cluster; N/A: not available.

Homology Search

SwiftOrtho detected 8,966,131,536 homologs in the *Bac* set within 1,247 CPU hours. Because it takes long time to perform all-*vs*-all BLASTP search on the full *Bac*, we randomly selected 1,000 protein sequences from *Bac* and searched them against the full *Bac* set. It took BLASTP 5.1 CPU hours to find the homologs of these 1,000 protein sequences. We infer that the estimated CPU time of BLASTP on the full

Bac set should be around 30,000 CPU hours. SwiftOrtho was almost 25 times faster than BLASTP on *Bac*.

Orthology Inference

SwiftOrtho, OrthoMCL, FastOrtho, and OrthAgogue were used to infer (co-)orthologs and in-paralogs from the homologs detected by the homology search module of SwiftOrtho in the *Bac* set. We did not test Orthofinder, because Orthofinder does not accept a single file of homologs as input. For the 1,760 proteomes in *Bac*, OrthoFinder needs to perform 3,097,600 pairwise species-by-species comparisons, which will generate the same number of files. Then, OrthoFinder performs the orthology inference on these 3,097,600 files. Even at one minute per file, it will take an estimated 6 CPU years to process all the files.

Due to memory limitations, only SwiftOrtho and FastOrtho finished the orthology inference on *Bac*. The results are shown in Table 3. The numbers of (co-)orthologs and in-paralogs inferred by SwiftOrtho and FastOrtho are similar. The number of common orthology relationships between SwiftOrtho and FastOrtho was 861,619,519 (98.2% of SwiftOrtho and 90.57% of FastOrtho). Compared with *Euk*, SwiftOrtho and FastOrtho have a similar predictive quality on *Bac*. There are three possible explainations for these results. The first is that *Euk* contains many protein isoforms which cause FastOrtho to overestimate the number of orthologs and in-paralogs. The second is that the gene duplication rate in Bacteria is lower than that in Eukaryotes [62, 63]. For *Bac*, each gene in one species has only small number of homolgs in other species, which makes FastOrtho unlikely to overestimate the number of RBHs. The third is that SwiftOrtho uses double-precision floating-point to store the E-value, which increases the precision of E-value from 10^{-180} to 10^{-308}. This improvement also reduces the possibility that FastOrtho may report false RBHs.

Computational resource use: Of the porgeams tested, only SwiftOrtho and FastOrtho finished the orthology inference step. FastOrtho and OrthAgogue did not finish the tests due to insufficient RAM; OrthoMCL aborted after running out of disk space, as it needed more than 18TB. The peak RAM usage of SwiftOrtho and FastOrtho were 90.6GB and 99.5GB, respectively. When we used the PyPy interpreter, the Peak RAM usage of SwiftOrtho was reduced to 72.1GB. FastOrtho was about 1.52 times faster than SwiftOrtho which ran the tests in the CPython interpreter. When using the PyPy interpreter, SwiftOrtho ran 1.58 times faster than FastOrtho. The memory usage and CPU time are shown in Table S7.

Cluster Analysis

The clustering numbers of SwiftOrtho and FastOrtho are similar. We compared the APC algorithm and the MCL algorithm, and APC found fewer clusters than MCL. The APC used much less memory and less CPU time than MCL. However, due to the lack of support for multi-threading and a large number of I/O operations, the real run time of APC is longer than that of MCL.

Tests on a Low-memory System

Because SwiftOrtho is designed to process large-scale data on low-memory computers, we used it to analyze *Bac* on a range of computers with different specifications. The results show that the memory usage of SwiftOrtho is flexible and adaptes to the size of the computer's memory. In the tests, SwiftOrtho finished orthology analysis of *Bac* set on a computer with only 4GB RAM in a reasonable time (Table S8).

Comparison with other Orthology Analysis Pipelines

SonicParanoid, OMA, and ProteinOrth are also graph-based methods and have been optimized for large-scale data sets [17, 31, 32]. We compared SwiftOrtho with them in both speed and memory usage. The results are shown in Table S10. OMA is very slow because it uses the Smith-Waterman algorithm to perform all-*vs*-all

alignment. In our tests, OMA took 0.84 CPU hours to align two species (4,064 and 4,140 genes) of *Bac* set. For *Bac* set, OMA needs to perform 3,097,600 species-by-species alignments and the total time will be over two million CPU hours. It is impractical to apply OMA to large-scale data set.

SonicParanoid worked well on *Euk* and *QfO 2011* sets. Compared with SwiftOrtho, SonicParanoid ran faster and required less RAM on small data sets. However, it exited abnormally when applied to *Bac* set.

Proteinortho also worked well on the *Euk* and *QfO 2011* sets. When applied to the *Bac* set, Proteinortho needs to perform 1,547,920 species-by-species proteome alignments. It took Proteinortho 186.5 CPU hours (using DIAMOND) to complete 23,331 (1.5%) alignments, we estimate that Proteinortho will take about 12,355 CPU hours to finish homology search. Since LAST is much faster than DIAMOND, we reran Proteinortho on *Bac* set, using LAST for homology search. The CPU time for LAST on *Bac* set was 2,368 hours. Although the previous results (Supplementary Table S9) show that LAST is about 20 times faster than SwiftOrtho, LAST took much more CPU time than SwiftOrtho in all-*vs*-all homology search step. We think it is because the species-by-species alignment approach requires more than 1.5 million I/O operations, which significantly reduces the speed. The CPU utilization of orthology inference and clustering of Proteinortho was very low (less than 10%) when applied to *Bac* set, which led to a very long real time (more than 150 hours); this is because Proteinortho occupied about 85% of physical memory when applied to large-scale data, which resulted in frequent data exchange between RAM and swap space and greatly reduced the speed. In sum, these results show that SwiftOrtho is a top performer on large-scale data.

Discussion

We present SwiftOrtho, a new high performance graph based homology classification tool. Unlike most tools that only perform orthology inference, SwiftOrtho integrates

all the modules necessary for a full orthology analysis, including homology search, orthology inference and cluster analysis. SwiftOrtho is designed to analyze large-scale genomic data on a normal desktop computer in a reasonable time. In our tests, SwiftOrtho's homology search module was nearly 30 times faster than BLASTP. The orthology inference module of SwiftOrtho was nearly 500 times faster than OrthoMCL when applied to *Euk*. When applied to the large-scale dataset, *Bac*, SwiftOrtho was the only one that finished orthology inference test on a workstation with 32GB RAM. The cluster module of SwiftOrtho using APC can handle data that is much larger than the computer memory. In our test, APC has comparable recall and accuracy, but requires much less memory than MCL. APC even improved F_1-measure score by increasing recall in most cases. With the help of these optimized modules, SwiftOrtho has successfully finished an orthology analysis of proteins from 1,760 bacterial genomes on a machine with only 4GB RAM, which makes SwiftOrho usable for large scale analyses for researchers who may not have access to expensive computational resources. SwiftOrtho is not only fast but also accurate, as shown in the results produced when running on orthobench and QfO[13, 33].

Conclusion

In summary, SwiftOrtho is a fast and accurate orthology prediction tool that can analyze a large number of sequences with minimal computational resource use. The installation and configuration of SwiftOrtho is simple and does not require the user to have any experience in database configuration. It is easy to use, as the only input required by SwiftOrtho is a FASTA format file of protein sequences with taxonomy information in the header line.

SwiftOrtho can be integrated into various common pipelines where fast orthology classification is required such as pan-genome analysis, large-scale phylogenetic tree construction, and other multi-genome analyses. It is specifically suited for microbial community analyses, where large number of sequences and species are involved.

Availability of supporting source code and requirements

The software and related information are listed below:

Project Name: SwiftOrtho

Project Home Page: https://github.com/Rinoahu/SwiftOrtho

Operating System(s): SwiftOrtho was tested on GNU/Linux distribution Ubuntu 16.04 64-bit, but we expect SwitOrtho to work on most *nix systems

Programming Language: Python

Other Requirements: Python 2.7, Python 3.7, PyPy2.7 v7.0 or higher

License: GPLv3

RRID: SCR_017122

Competing interests
The authors declare that they have no competing interests.

Author's contributions
Text for this section . . .

Acknowledgements
Text for this section . . .

References
1. Koonin, E.V.: Orthologs, paralogs, and evolutionary genomics. Annu. Rev. Genet. (2005). doi:10.1146/annurev.genet.39.073003.114725
2. Fitch, W.M.: Distinguishing Homologous from Analogous Proteins. Syst. Zool. **19**(2), 99 (1970). doi:10.2307/2412448
3. Overbeek, R., Fonstein, M., D 'souza, M., Pusch, G.D., Maltsev, N.: The use of gene clusters to infer functional coupling. Genetics **96**, 2896–2901 (1999)
4. Rivera, M.C., Jain, R., Moore, J.E., Lake, J.A.: Genomic evidence for two functionally distinct gene classes. Genetics **95**, 6239–6244 (1998)
5. Remm, M., Storm, C.E.V.V., Sonnhammer, E.L.L.L.: Automatic clustering of orthologs and in-paralogs from pairwise species comparisons. J. Mol. Biol. **314**(5), 1041–1052 (2001). doi:10.1006/jmbi.2000.5197
6. O'Brien, K.P., Remm, M., Sonnhammer, E.L.L.: Inparanoid: a comprehensive database of eukaryotic orthologs. Nucleic Acids Res. **33**(Database issue), 476–80 (2005). doi:10.1093/nar/gki107
7. Gabaldón, T., Koonin, E.V.: . Nature Reviews Genetics **14**(5), 360–366 (2013). doi:10.1038/nrg3456
8. Goodman, M., Czelusniak, J., Moore, G.W., Romero-Herrera, A.E., Matsuda, G.: Fitting the Gene Lineage into its Species Lineage, a Parsimony Strategy Illustrated by Cladograms Constructed from Globin Sequences. Syst. Biol. **28**(2), 132–163 (1979). doi:10.1093/sysbio/28.2.132
9. Kristensen, D.M., Wolf, Y.I., Mushegian, A.R., Koonin, E.V.: . Briefings in bioinformatics **12**(5), 379–91 (2011). doi:10.1093/bib/bbr030
10. Gabaldón, T.: Large-scale assignment of orthology: back to phylogenetics? Genome Biol. **9**(10), 235 (2008). doi:10.1186/gb-2008-9-10-235
11. Hulsen, T., Huynen, M.A., de Vlieg, J., Groenen, P.M.A.: Benchmarking ortholog identification methods using functional genomics data. Genome Biol. **7**(4), 31 (2006). doi:10.1186/gb-2006-7-4-r31
12. Kuzniar, A., van Ham, R.C.H.J., Pongor, S., Leunissen, J.A.M.: The quest for orthologs: finding the corresponding gene across genomes (2008). doi:10.1016/j.tig.2008.08.009
13. Trachana, K., Larsson, T.A., Powell, S., Chen, W.-H., Doerks, T., Muller, J., Bork, P.: Orthology prediction methods: a quality assessment using curated protein families. Bioessays **33**(10), 769–80 (2011). doi:10.1002/bies.201100062
14. Ward, N., Moreno-Hagelsieb, G.: Quickly finding orthologs as reciprocal best hits with BLAT, LAST, and UBLAST: How much do we miss? PLoS One **9**(7) (2014). doi:10.1371/journal.pone.0101850
15. Tatusov, R.L., Galperin, M.Y., Natale, D.A., Koonin, E.V.: The COG database: a tool for genome-scale analysis of protein functions and evolution. Nucleic Acids Res. **28**(1), 33–36 (2000). doi:10.1093/nar/28.1.33
16. Roth, A.C.J., Gonnet, G.H., Dessimoz, C.: Algorithm of OMA for large-scale orthology inference. BMC Bioinformatics **9**(1), 518 (2008). doi:10.1186/1471-2105-9-518
17. Altenhoff, A.M., Glover, N.M., Train, C.M., Kaleb, K., Warwick Vesztrocy, A., Dylus, D., De Farias, T.M., Zile, K., Stevenson, C., Long, J., Redestig, H., Gonnet, G.H., Dessimoz, C.: The OMA orthology database in 2018: Retrieving evolutionary relationships among all domains of life through richer web and programmatic interfaces. Nucleic Acids Res. (2018). doi:10.1093/nar/gkx1019
18. Alexeyenko, A., Tamas, I., Liu, G., Sonnhammer, E.L.L.: Automatic clustering of orthologs and inparalogs shared by multiple proteomes. In: Bioinformatics (2006). doi:10.1093/bioinformatics/btl213
19. Li, M., Ma, B., Kisman, D., Tromp, J.: PatternHunter II: highly sensitive and fast homology search. Genome Inform. **14**(03), 164–75 (2003). doi:10.1142/S0219720004000661
20. Fischer, S., Brunk, B.P., Chen, F., Gao, X., Harb, O.S., Iodice, J.B., Shanmugam, D., Roos, D.S., Stoeckert, C.J.: Using OrthoMCL to assign proteins to OrthoMCL-DB groups or to cluster proteomes into new ortholog groups. Curr. Protoc. Bioinforma. (2011). doi:10.1002/0471250953.bi0612s35
21. van Dongen, S.: Graph clustering by flow simulation. Graph Stimul. by flow Clust. **PhD thesis**, (2000). doi:10.1016/j.cosrev.2007.05.001
22. Sonnhammer, E.L.L., Koonin, E.V.: Orthology, paralogy and proposed classification for paralog subtypes. Trends Genet. **18**(12), 619–620 (2002). doi:10.1016/S0168-9525(02)02793-2
23. Cannon, S.B., Young, N.D.: OrthoParaMap: Distinguishing orthologs from paralogs by integrating comparative genome data and gene phylogenies. BMC Bioinformatics (2003). doi:10.1186/1471-2105-4-35
24. Cutts, T., Down, T., Dyer, S.C., Fitzgerald, S., Fernandez-Banet, J., Graf, S., Haider, S., Hammond, M., Herrero, J., Holland, R., Hubbard, T.J.P., Howe, K., Johnson, N., Kahari, A.,

Keefe, D., Kokocinski, F., Kulesha, E., Lawson, D., Longden, I., Melsopp, C., Aken, B.L., Megy, K., Meidl, P., Ouverdin, B., Parker, A., Prlic, A., Rice, S., Rios, D., Schuster, M., Sealy, I., Severin, J., Beal, K., Slater, G., Smedley, D., Spudich, G., Trevanion, S., Vilella, A., Vogel, J., White, S., Wood, M., Cox, T., Curwen, V., Ballester, B., Durbin, R., Fernandez-Suarez, X.M., Flicek, P., Kasprzyk, A., Proctor, G., Searle, S., Smith, J., Ureta-Vidal, A., Birney, E., Caccamo, M., Chen, Y., Clarke, L., Coates, G., Cunningham, F.: Ensembl 2007. Nucl. Acids Res. (2007). doi:10.1093/nar/gkl996

25. Ruan, J., Li, H., Chen, Z., Coghlan, A., Coin, L.J.M., Guo, Y., Hériché, J.K., Hu, Y., Kristiansen, K., Li, R., Liu, T., Moses, A., Qin, J., Vang, S., Vilella, A.J., Ureta-Vidal, A., Bolund, L., Wang, J., Durbin, R.: TreeFam: 2008 Update. Nucleic Acids Res. (2008). doi:10.1093/nar/gkm1005
26. Goodstadt, L., Ponting, C.P.: Phylogenetic reconstruction of orthology, paralogy, and conserved synteny for dog and human. PLoS Comput. Biol. (2006). doi:10.1371/journal.pcbi.0020133
27. Vilella, A.J., Severin, J., Ureta-Vidal, A., Heng, L., Durbin, R., Birney, E.: EnsemblCompara GeneTrees: Complete, duplication-aware phylogenetic trees in vertebrates. Genome research **19**(2), 327–35 (2009). doi:10.1101/gr.073585.107
28. Chen, F., Mackey, A.J., Vermunt, J.K., Roos, D.S.: Assessing performance of orthology detection strategies applied to eukaryotic genomes. PLoS One **2**(4), 383 (2007). doi:10.1371/journal.pone.0000383
29. Altenhoff, A.M., Dessimoz, C.: Phylogenetic and functional assessment of orthologs inference projects and methods. PLoS Comput. Biol. **5**(1), 1000262 (2009). doi:10.1371/journal.pcbi.1000262
30. Sonnhammer, E.L.L., Östlund, G.: InParanoid 8: orthology analysis between 273 proteomes, mostly eukaryotic. Nucleic acids research **43**(Database issue), 234–9 (2015). doi:10.1093/nar/gku1203
31. Cosentino, S., Iwasaki, W.: SonicParanoid: Fast, accurate and easy orthology inference. Bioinformatics (2019). doi:10.1093/bioinformatics/bty631
32. Lechner, M., Findeiß, S., Steiner, L., Marz, M., Stadler, P.F., Prohaska, S.J.: Proteinortho: Detection of (Co-)orthologs in large-scale analysis. BMC Bioinformatics (2011). doi:10.1186/1471-2105-12-124
33. Altenhoff, A.M., Boeckmann, B., Capella-Gutierrez, S., Dalquen, D.A., DeLuca, T., Forslund, K., Huerta-Cepas, J., Linard, B., Pereira, C., Pryszcz, L.P., Schreiber, F., Da Silva, A.S., Szklarczyk, D., Train, C.M., Bork, P., Lecompte, O., Von Mering, C., Xenarios, I., Sjölander, K., Jensen, L.J., Martin, M.J., Muffato, M., Gabaldón, T., Lewis, S.E., Thomas, P.D., Sonnhammer, E., Dessimoz, C.: Standardized benchmarking in the quest for orthologs. Nat. Methods (2016). doi:10.1038/nmeth.3830
34. Pearson, W.R., Lipman, D.J.: Improved tools for biological sequence comparison. Proc. Natl. Acad. Sci. **85**(8), 2444–2448 (1988). doi:10.1073/pnas.85.8.2444
35. Altschul, S.F., Gish, W., Miller, W., Myers, E.W., Lipman, D.J.: Basic local alignment search tool. J. Mol. Biol. **215**(3), 403–410 (1990). doi:10.1016/S0022-2836(05)80360-2. arXiv:1611.08307v1
36. Kent, W.J.: BLAT — The BLAST -Like Alignment Tool. Genome Research **12**, 656–664 (2002). doi:10.1101/gr.229202.
37. Shiryev, S.A., Papadopoulos, J.S., Schäffer, A.A., Agarwala, R., Schaffer, A.A., Agarwala, R.: Improved BLAST searches using longer words for protein seeding. Bioinformatics **23**(21), 2949–2951 (2007). doi:10.1093/bioinformatics/btm479
38. Ma, B., Tromp, J., Li, M.: PatternHunter: faster and more sensitive homology search. Bioinformatics **18**(3), 440–445 (2002). doi:10.1093/bioinformatics/18.3.440
39. Ilie, L., Ilie, S., Khoshraftar, S., Bigvand, A.M.: Seeds for effective oligonucleotide design. BMC Genomics **12**(1), 280 (2011). doi:10.1186/1471-2164-12-280
40. Edgar, R.C.: Search and clustering orders of magnitude faster than BLAST. Bioinformatics **26**(19), 2460–2461 (2010). doi:10.1093/bioinformatics/btq461. Edgar, Robert C., 2010, Search
41. Kiełbasa, S.M., Wan, R., Sato, K., Horton, P., Frith, M.C.: Adaptive seeds tame genomic sequence comparison. Genome Res. **21**(3), 487–493 (2011). doi:10.1101/gr.113985.110
42. Buchfink, B., Xie, C., Huson, D.H.: Fast and sensitive protein alignment using DIAMOND (2014). doi:10.1038/nmeth.3176
43. Smith, T.F., Waterman, M.S.: Identification of common molecular subsequences. J. Mol. Biol. **147**(1), 195–197 (1981). doi:10.1016/0022-2836(81)90087-5
44. Chao, K.M., Pearson, W.R., Miller, W.: Aligning two sequences within a specified diagonal band. Bioinformatics **8**(5), 481–487 (1992). doi:10.1093/bioinformatics/8.5.481
45. Landès, C., Risler, J.L.: Fast databank searching with a reduced amino-acid alphabet. Computer applications in the biosciences : CABIOS **10**(4), 453–454 (1994)
46. Murphy, L.R., Wallqvist, A., Levy, R.M.: Simplified amino acid alphabets for protein fold recognition and implications for folding. Protein Eng. Des. Sel. **13**(3), 149–152 (2000). doi:10.1093/protein/13.3.149
47. Peterson, E.L., Kondev, J., Theriot, J.A., Phillips, R.: Reduced amino acid alphabets exhibit an improved sensitivity and selectivity in fold assignment. Bioinformatics (Oxford, England) **25**(11), 1356–1362 (2009). doi:10.1093/bioinformatics/btp164
48. Edgar, R.C.: Local homology recognition and distance measures in linear time using compressed amino acid alphabets. Nucleic acids research **32**(1), 380–5 (2004). doi:10.1093/nar/gkh180
49. Ye, Y., Choi, J.-H., Tang, H.: RAPSearch: a fast protein similarity search tool for short reads. BMC Bioinformatics **12**(1), 159 (2011). doi:10.1186/1471-2105-12-159
50. Gibbons, T.R., Mount, S.M., Cooper, E.D., Delwiche, C.F.: Evaluation of BLAST-based edge-weighting metrics used for homology inference with the Markov Clustering algorithm. BMC

Bioinformatics **16**(1) (2015). doi:10.1186/s12859-015-0625-x
51. Enright, A.J., Van Dongen, S., Ouzounis, C.A.: An efficient algorithm for large-scale detection of protein families. Nucleic Acids Res. **30**(7), 1575–1584 (2002). doi:10.1093/nar/30.7.1575. journal.pone.0035671
52. Emms, D.M., Kelly, S.: OrthoFinder: solving fundamental biases in whole genome comparisons dramatically improves orthogroup inference accuracy. Genome Biology **16**(1), 157 (2015). doi:10.1186/s13059-015-0721-2
53. Davis, J.J., Gerdes, S., Olsen, G.J., Olson, R., Pusch, G.D., Shukla, M., Vonstein, V., Wattam, A.R., Yoo, H.: PATtyFams: Protein families for the microbial genomes in the PATRIC database. Front. Microbiol. **7**(FEB), 118 (2016). doi:10.3389/fmicb.2016.00118
54. Frey, B.J., Dueck, D.: Clustering by passing messages between data points. Science **315**(5814), 972–6 (2007). doi:10.1126/science.1136800. 1401.2548
55. Lam, S.K., Pitrou, A., Seibert, S.: Numba: A LLVM-based python JIT compiler. Proc. Second Work. LLVM Compil. Infrastruct. HPC - LLVM '15, 1–6 (2015). doi:10.1145/2833157.2833162
56. Curwen, V., Eyras, E., Andrews, T.D., Clarke, L., Mongin, E., Searle, S.M.J., Clamp, M.: The Ensembl automatic gene annotation system. Genome Res. **14**(5), 942–950 (2004). doi:10.1101/gr.1858004
57. Benson, D.A.: GenBank. Nucleic Acids Res. **28**(1), 15–18 (2000). doi:10.1093/nar/28.1.15
58. Camacho, C., Coulouris, G., Avagyan, V., Ma, N., Papadopoulos, J., Bealer, K., Madden, T.L.: BLAST+: architecture and applications. BMC Bioinformatics (2009). doi:10.1186/1471-2105-10-421
59. Brohée, S., van Helden, J.: Evaluation of clustering algorithms for protein-protein interaction networks. BMC Bioinformatics (2006). doi:10.1186/1471-2105-7-488
60. Medlar, A., Holm, L.: TOPAZ: Asymmetric suffix array neighbourhood search for massive protein databases. BMC Bioinformatics (2018). doi:10.1186/s12859-018-2290-3
61. Rigo, A., Pedroni, S.: PyPy ' s Approach to Virtual Machine Construction. Companion to 21st ACM SIGPLAN Symp., 944–953 (2006). doi:10.1145/1176617.1176753
62. Bratlie, M.S., Johansen, J., Sherman, B.T., Huang, D.W., Lempicki, R.A., Drabløs, F.: Gene duplications in prokaryotes can be associated with environmental adaptation. BMC Genomics (2010). doi:10.1186/1471-2164-11-588
63. Katju, V., Bergthorsson, U.: Copy-number changes in evolution: Rates, fitness effects and adaptive significance (2013). doi:10.3389/fgene.2013.00273

Figures

Figure 1 The flow cart of SwiftOrtho. SwiftOrtho is a graph-based method which consist of three major steps: **All-*vs*-All Homology Search**: A seed-and-extension method is used to perform homology search; **Orthology Inference:** Nodes are gene names, edges are similarity score of pairwise genes. 1. A_1-B_1 are putative orthologs identified by RBH. 2. A_1-A_2 and B_1-B_2 are putative in-paralogs as the bit scores of these pairs greater than A_1-B_1; 3. A_2-B_1 and A_2-B_2 are putative co-orthologs as these pairs are not orthologs but A_1-B_1 are orthologs and A_1-A_2, B_1-B_2 are in-paralogs; **Cluster Analysis:** Markov clustering or Affinity Propagation Algorithm is used to cluster orthology relationships.

Figure 2 Comparing Standard Smith-Waterman with Banded Smith-Waterman. A. Similarity score matrix for Standard Smith-Waterman. Standard Smith-Waterman algorithm need to calculate all the entries. **B.** Similarity score matrix for Banded Smith-Waterman. Banded Smith-Waterman algorithm only need to calculate the entries on and near the diagonal.

Figure 3 Evaluation of predicted orthologous groups. Evaluation of different tools on OrthoBench database. **SO+MCL**: SwiftOrtho with MCL; **SO+APC**: SwiftOrtho with Affinity Propagation Clustering; **OM**: OrthoMCL v2; **FO**: FastOrtho; **OA**: OrthAgogue; **OF**: OrthoFinder.

Figure 4 Comparing BLASTP and SwiftOrtho's homology search module on the quality of orthologous groups prediction. BLASTP and SwiftOrtho's search module perform an all-*vs*-all search on the *Euk* set, respectively. Then, all the orthology prediction tools were employed for orthology inference. Finally, the predicted orthology relationships were clustered into orthologous groups by MCL algorithm.

Figure 5 Markov Clustering versus Affinity Propagation Clustering. Both algorithms were applied to cluster the orthology relationships of the Euck set inferred by different orthology prediction tools, into orthologous groups. As OrthoFinder does not report orthology relationships, the Affinity Propagation can not be applied to its results. **MCL:** Markov Clustering algorithm; **APC:** Affinity Propagation Clustering.

Figure 6 The Benchmarking in Quest for Orthologs. A: Species Tree Discordance Benchmark. Inparanoid has minimum average Robinson-Foulds distance. SwiftOrtho's average RF distance is close to that of Inparanoid. The prediction inferred by OrthoFinder is not aviable for this test; **B:** Generalized Species Tree Discordance Benchmark. InParanoid has minimum average Robinson-Foulds distance. The prediction inferred by OrthoFinder is not aviable for this test; **C:** Agreement with the Reference Gene Phylogenies of SwissTree. SwiftOrtho has the highest positive prediction value rate(Recall). InParanoid has the highest true positive rate (precision); **D:** Agreement with Reference Gene Phylogenies of TreeFam-A. SonicParanoid has the highest positive prediction value rate (recall), however, its true positive rate (precision) is close to zero. SwiftOrtho has the second highest recall and precision; **E:** Gene Ontology conservation test. OrthoMCL has the highest average Schlicker similarity; **F:** Enzyme Classification conservation test. SwiftOrtho has the highest average Schlicker similarity. OrthoMCL detected the most orthology relationships and has the highest recall.

Tables

Additional Files

Additional file 1 —
Metadata for the genome assemblies of the *Bac* set (tab-delimited text file).
https://figshare.com/s/19a006d6fea9c2494ab8

Figure 1 Click here to access/download;Figure;F1_swo_flow.eps

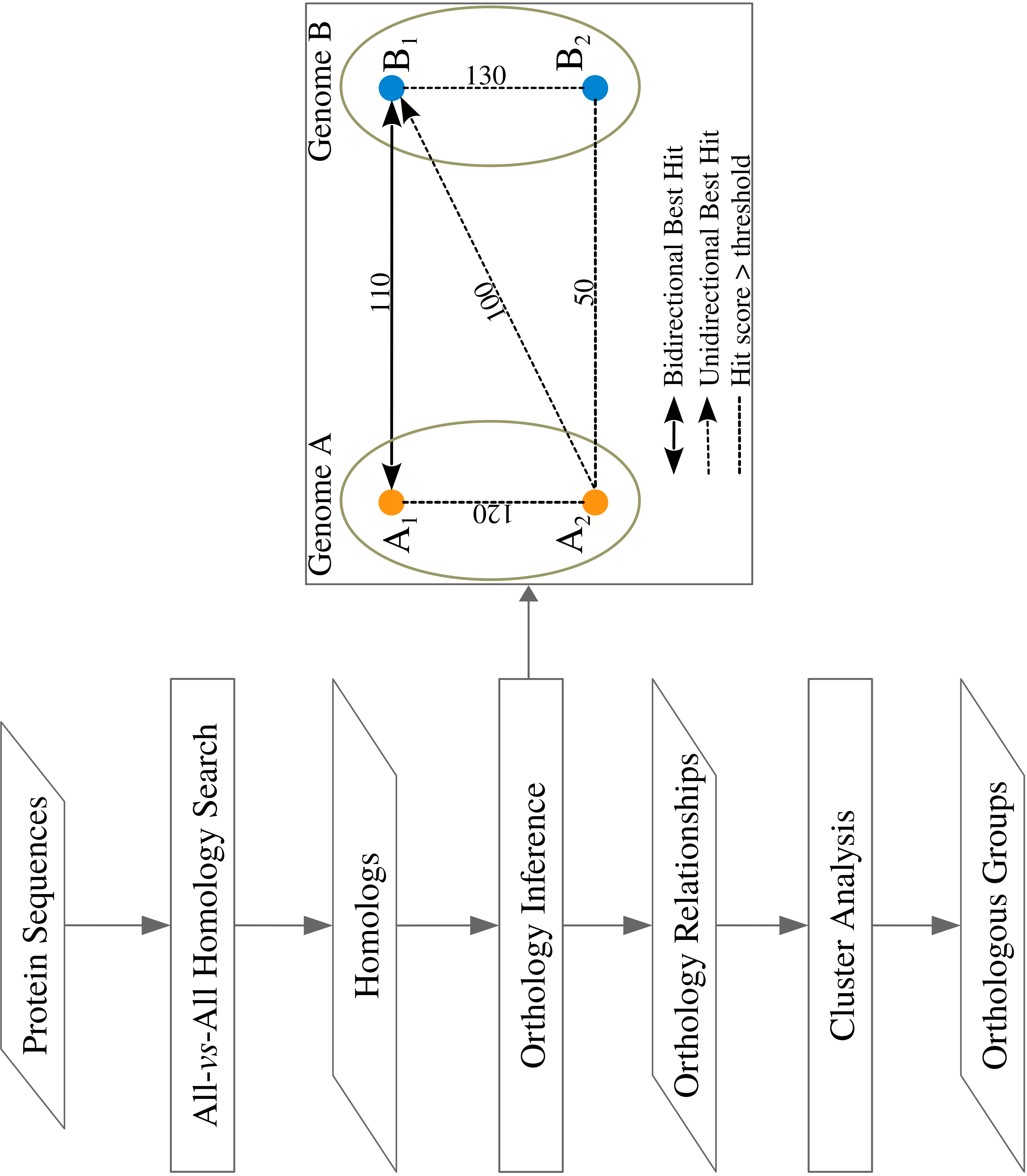

Figure 2

Click here to access/download;Figure;F2_bandSW.eps

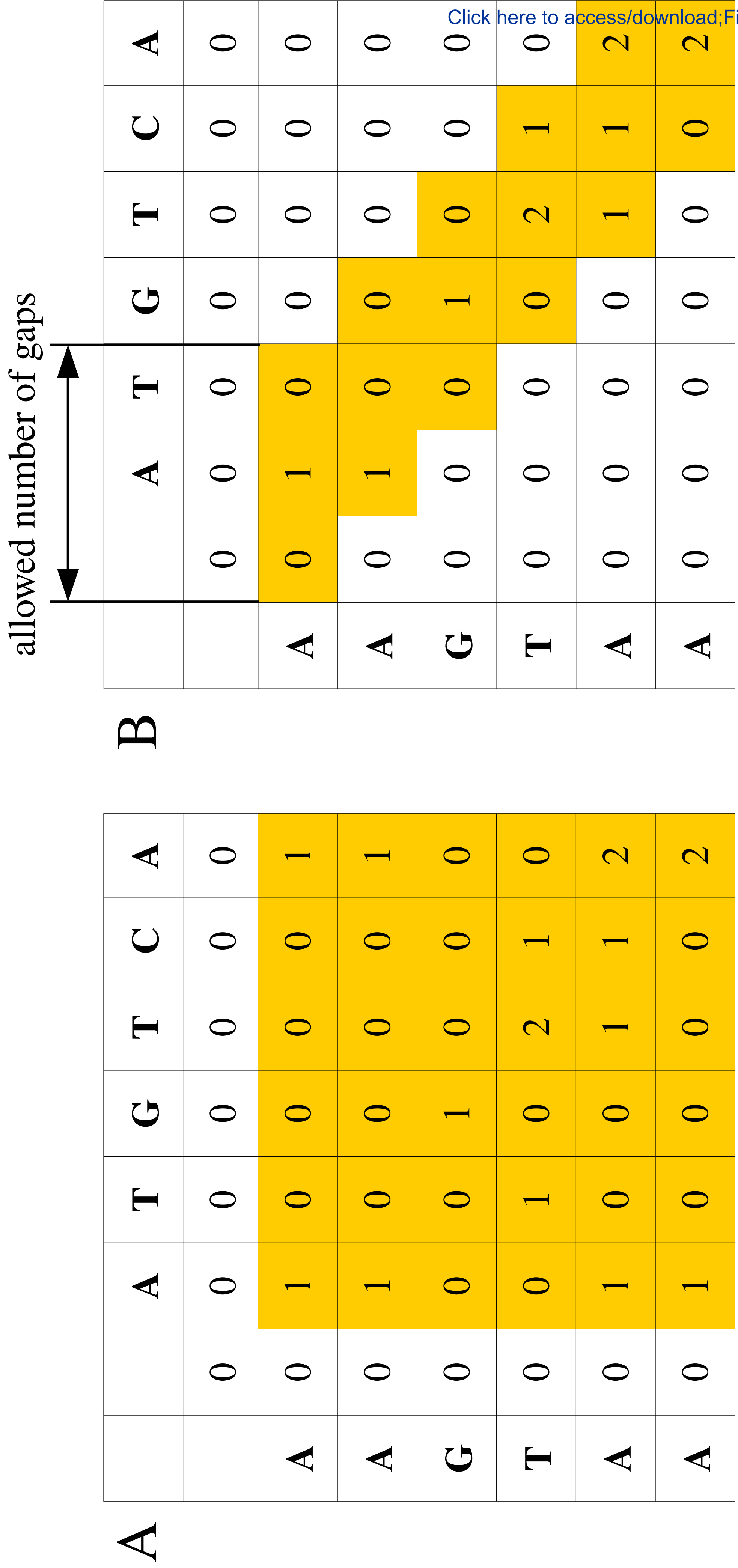

Figure 3

Click here to access/download;Figure;F3_recall_acc.eps

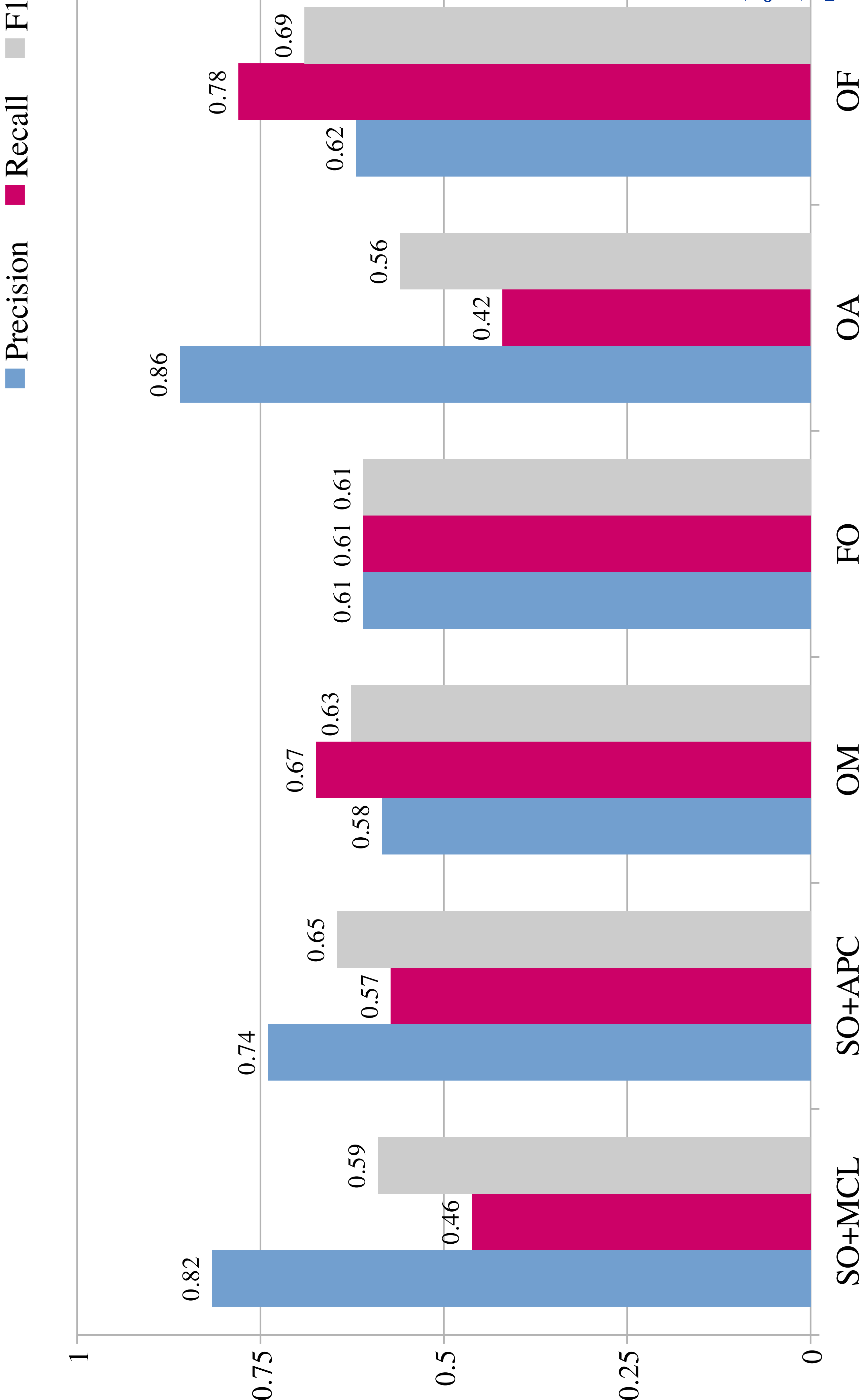

Figure 4

Click here to access/download;Figure;F4_blastp_vs_so.eps

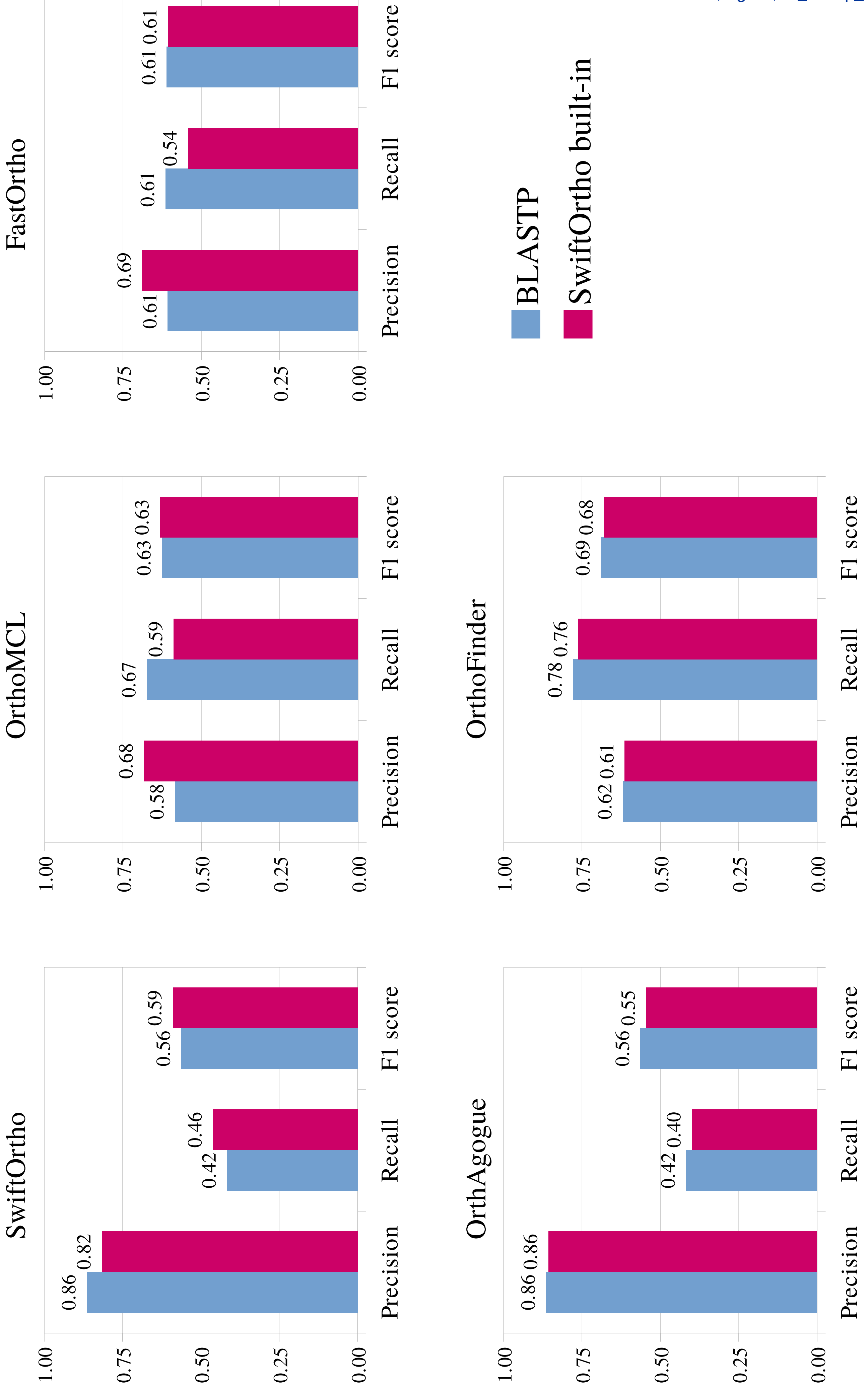

Figure 5

Click here to access/download;Figure;F5_comp_mcl_apc.eps

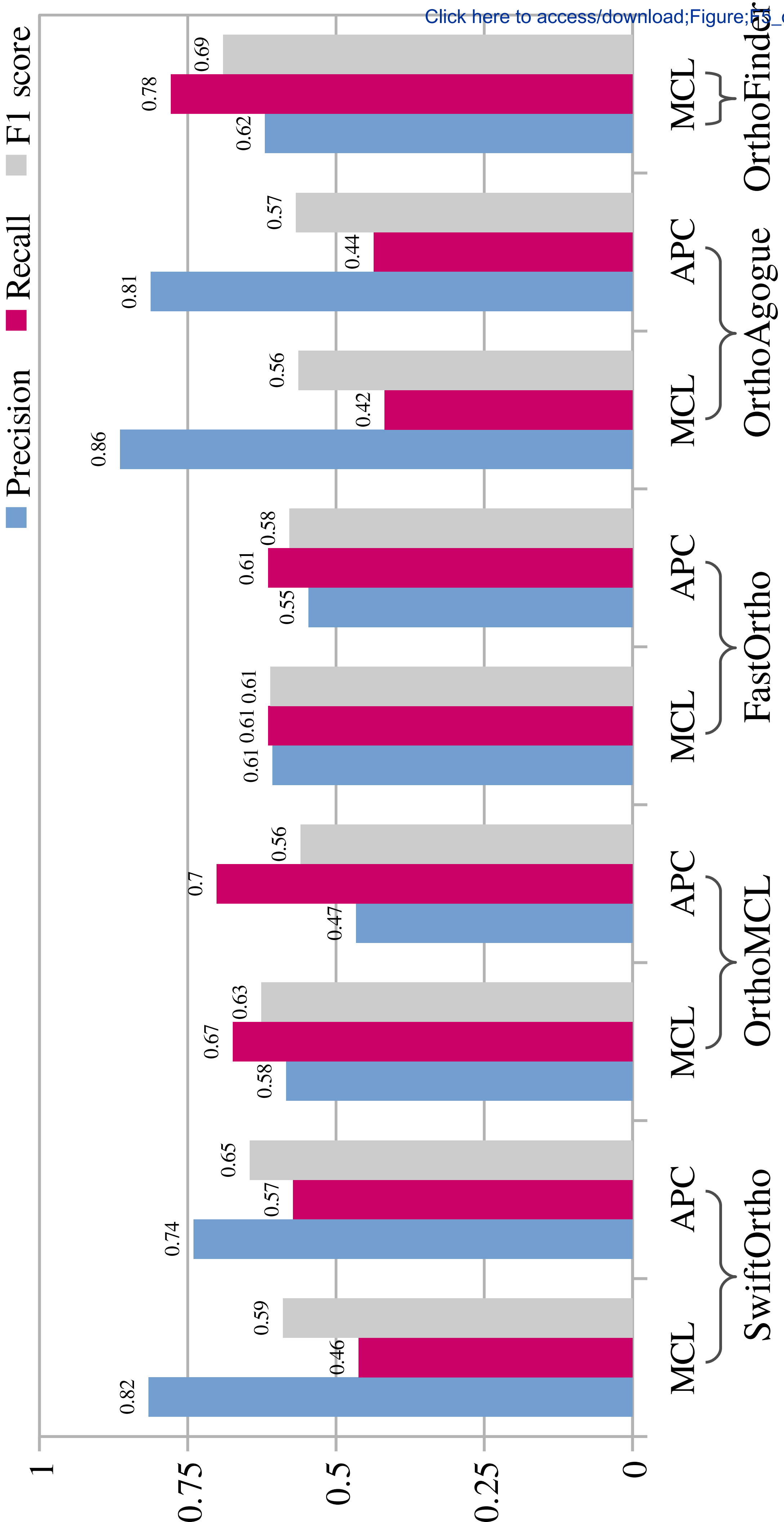

Figure 6

Click here to access/download;Figure;F6_s_qfo.eps

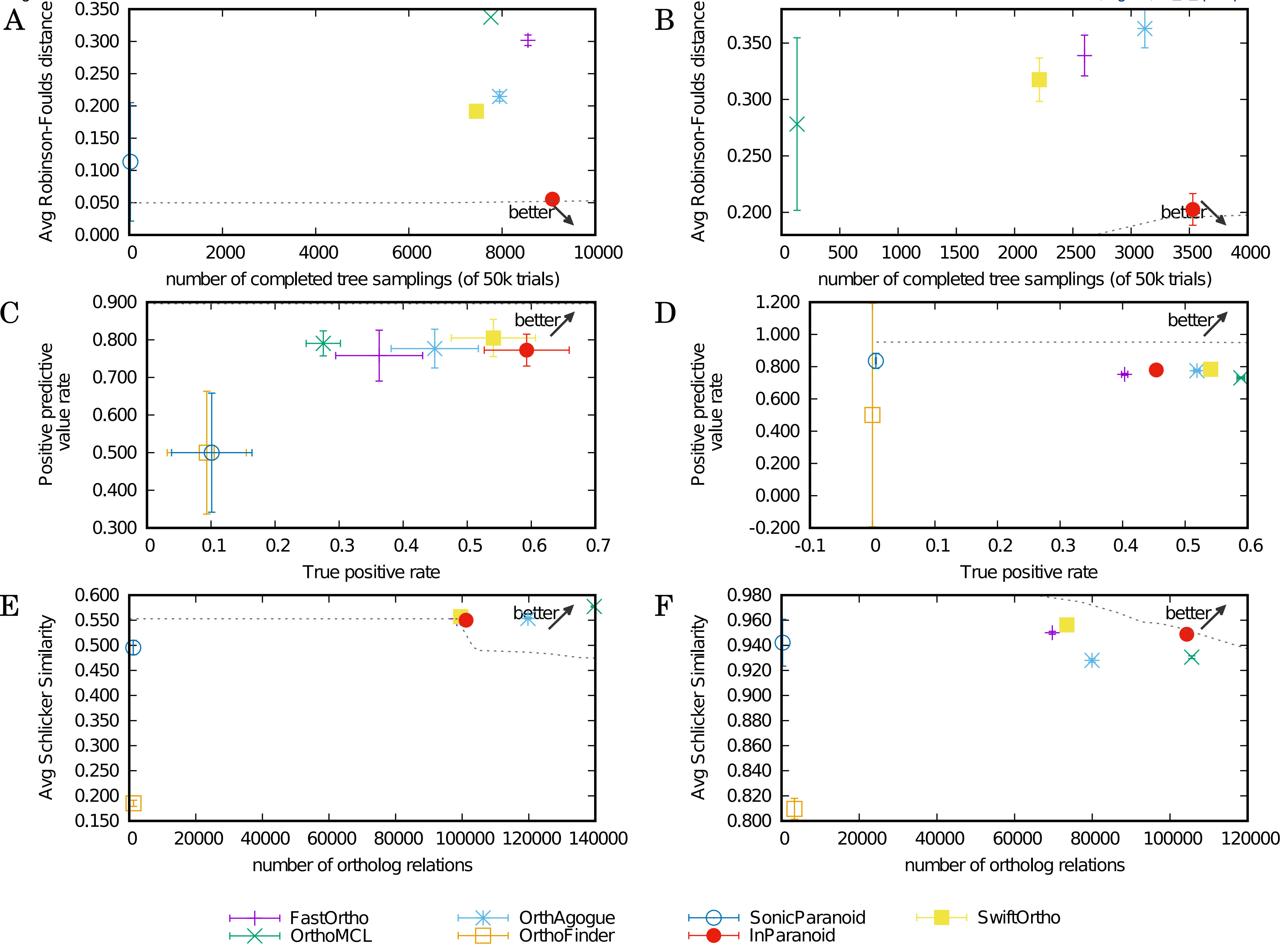

Figure S1

Click here to access/download;Figure;S1_evaluate_OGs.eps

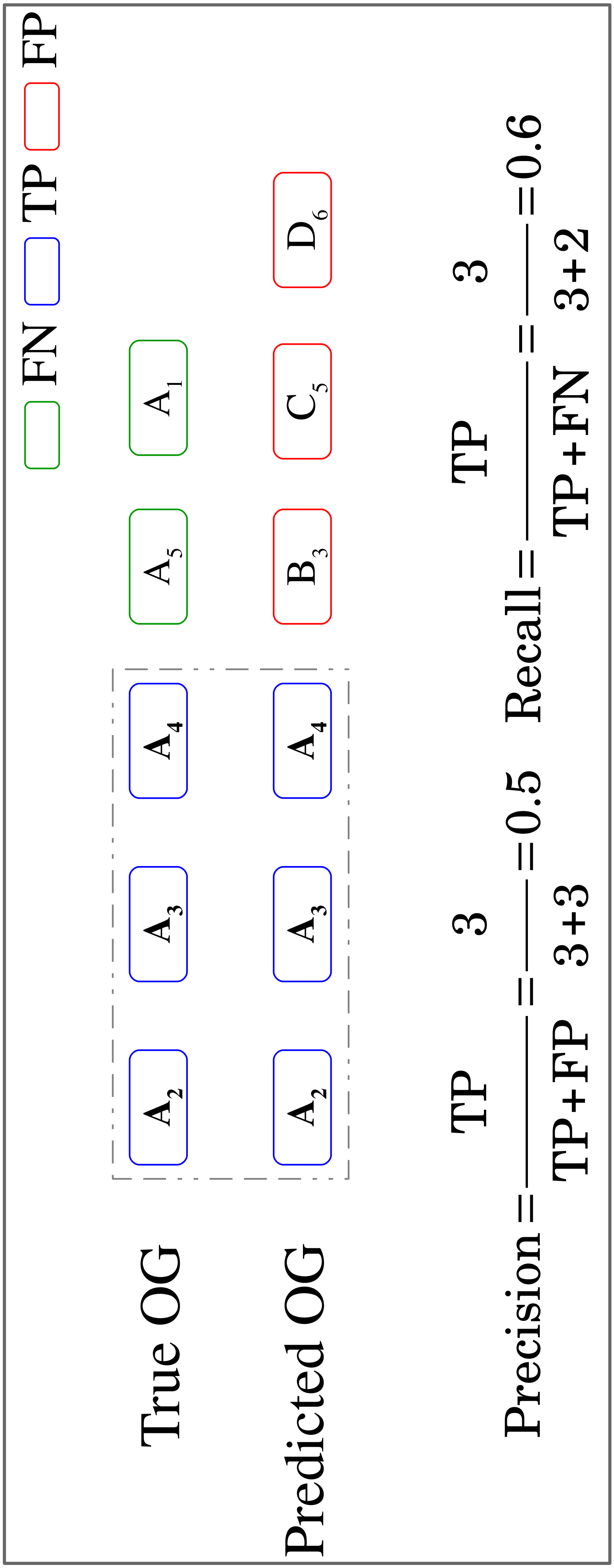

Click here to access/download
Supplementary Material
Supp_SwiftOrtho_note_2_gigascience_revised.pdf

Personal Cover
Click here to access/download;Personal Cover;SwiftOrtho Rebuttal Letter.pdf

IOWA STATE UNIVERSITY
OF SCIENCE AND TECHNOLOGY

Hans Zauner, PhD
Editor, GigaScience
Oxford University Press

Iddo Friedberg, PhD
Associate Professor
College of Veterinary Medicine
Iowa State University
Ames, IA 50011
T: +1 515 294 5959
E: idoerg@iastate.edu

June 6, 2019

Dear Dr. Zauner,

Enclosed please find our revised manuscript. We would like to thank the reviewers and yourself for the large amount of time and effort that were spent reading and commenting on the manuscript. We are grateful for this effort, and we have addressed all comments in detail. We would like to stress that SwiftOrtho's strength lies both in its modular versatility, and in its low consumption of computational resources, making it especially suitable for low- and medium budget labs, but is easy and accurate enough to be used universally.

We have upgraded the software to Python 3, and rewrote the code to conform with PEP-8. We have also compared with all the other tools requested, and some others, including DIAMOND, Usearch, TOPAZ, LAST and BLAT.

We are happy to include the paper in the Technical Notes section. We have registered SwiftOrtho in SciCrunch.org, and added the Software Availability section, and the availability of supporting source code and requirements.

Below are the reviewers' requests, and our detailed responses are in italics. We are looking forward to your feedback.

Sincerely,

I. Friedberg

Iddo Friedberg

(Reviewer 1)

The paper of Hu and Friedberg presents an interesting new method for large-scale identification of orthologous groups without the need for large-scale compute servers. The use of spaced seeds and reduced amino alphabets are innovative and interesting, and the method has a high precision across benchmarks, compared to its competitors. While the method is interesting and has added value, I think there are a number of ways in which the paper and the tool could be improved. I have ordered my recommendations in a few different categories.

Scientific:

* The key point of the paper seems to be that SwiftOrtho requires less memory and CPU compared to other tools, yet there is no figure or table that compares these characteristics across tools for datasets of different sizes. I would strongly recommend adding this to actually show the difference with each of the other tools.

Orthology analysis has three steps: 1) homology search, 2) orthology inference, 3) clustering orthology relationships into orthologous groups. We have compared SwiftOrtho with other tools in each of these steps, and the results are already in the Supplementary Table S5, ,S6, S7, and S8:

1. *In Supplementary Table S5, ,S6 and S7, we have compared CPU time usage of SwiftOrtho with BLASTP in homology search on different sized data sets. The results show that SwiftOrtho is about 30-60 times faster than BLASTP.*
2. *In Supplementary Table S5, ,S6 and S7, we have compared the memory and CPU time usage of orthology inference of SwiftOrtho with other software on different data sets. The results show SwiftOrtho is fast and requires less memory than other tools. For example, in Table S7, only SwiftOrtho and FastOrtho finished orthology inference on the Bac set (1,760 genomes).*
3. *In Table S7, we compared two clustering algorithm of MCL and APC. The results show APC requires less memory than MCL.*
4. *To further show the performance of SwiftOrtho, we also used it to analyze Bac on several hardware configurations. Supplementary Table S8 shows that SwiftOrtho with APC algorithm finished orthology analysis of Bac on a virtual machine with 4GB RAM. None of orthology inference or MCL algorithm can run on the this virtual machine.*

* Line 227: SwiftOrtho is compared to BlastP, but not to other fast equivalents such as Diamond or Usearch. I think this comparison should be added, to show the added value of the method compared to the current state of the art.

Thank you for this comment. It should be noted that the orthology prediction tools we compared with using BLASTP as the default sequence alignment tool. That's why we only used BLASTP. However, we also added a comparison of SwiftOrtho with

BLASTP, LAST, Topaz, Diamond, BLAT, and Usearch. The new benchmark results can be found in Table S9. The results show that SwfitOrtho has a good trade-off between speed and recall.

General writing:

* The introduction does not read fluently. It is a bit 'staccato', without clear connections and transitions between the parts. I think this can use some editing to more clearly describe the field, its relevance (which is only explained in vague terms at the moment), the state of the art, the challenge and the contribution by the authors.

We have now improved the Introduction based on this comment, and added the points requested by the reviewer.

* It is surprising that the methodology for orthology classification (which is what makes it fast and memory-efficient) is not mentioned in the abstract.

We added more details in the abstract: "SwiftOrtho uses the long k-mers to speed up homology search, utilizes a reduced amino acid alphabet and spaced seeds to compensate for the loss of sensitivity due to long k-mers. SwiftOrtho uses Affinity Propagation algorithm to cluster large-scale data sets." The part has been highlighted in red.

* Line 14: Inparanoid is mentioned without introducing it.

We have now introduced InParanoid, and added a few details. See lines: 17-20 " ...Inparanoid uses RBH method to identify orthologs between two species, the genes in the two species will be identified as the in-paralogs if they are more similar to the corresponding ortholog than to any gene in the other species..."

* The figures could use a make-over, e.g. by removing horizontal/vertical skewing, changing fonts and applying a more pleasing color scheme.

We changed fonts and color scheme in Figure 2 and 3, and removed the skewing.

* It would be very helpful to have a flowchart-like figure that outlines the different steps taken by the SwiftOrtho algorithm.

We have now added an explanatory flowchart to Figure 1.

* Some of the figures (e.g., Fig 3a) are unnecessary, as they explain things that can safely be assumed to be textbook knowledge.

It is difficult to know what is textbook knowledge for different groups of readers in a general-audience journal such as GigaScience. We would therefore rather err on the side of being inclusive. We believe it is better to have some readers read things they already know and consider textbook, rather than omit too much and lose readers. That being said, we have removed Fig 3a and placed it as Figure S1.

* The use of horizontal scales in Figure 6 is puzzling, e.g. the -0.200 lower end for

Fig. 6D. Also, this makes OrthoFinder looks unrealistically bad in panel F.

The value on x-axis for orthofinder is nearly zero. This is the performance we observed in Swiftortho.

Grammar/spelling:
A thorough round of copy-editing would be highly recommended, as there are several spelling/grammar/style issues throughout. Some examples are:

* Line 25: limitation -> limiting factor
* Line 51: data set -> data sets (also in line 46)
* Line 401: the sentence is broken.
* Figure legend of Figure 5: OrthFinder -> OrthoFinder, 'can not apply' -> 'cannot be applied'

Thank you for these comments. We have now fixed these errors, and proofread the manuscript carefully.

Code/software:

* In my honest opinion, the code needs some serious refactoring; it has many global variables (code outside functions/classes), has several commented out sections, largely lacks proper documentation (with docstrings etc.; see https://www.python.org/dev/peps/pep-0008/) and has many functions that are far too long (and need to be broken up into smaller ones). Additionally, implementing tests would ensure that the code functions as intended.

We reformatted the code to PEP-8 style. The Global Variables we used are the score matrices or char to int tables. If we put these variables in a function, each time the function is called, it would need to regenerate these matrices or tables, which adds unnecessary computational overhead. We have also implemented user testing.

* The readme should detail which versions are needed of the packages that are required.

We added version requirements. We also added an installation script which will find the packages of correct versions.

* SwiftOrtho is written and distributed in Python 2.7, which is going to be retired very soon (see https://pythonclock.org/). Updating to Python3 is highly recommended for durability of the software. This is not difficult to do.

We rewrote the code in Python3.

(Reviewer 2)

Hu and Friedberg present an orthology prediction tool named SwiftOrtho. It is geared towards low memory and CPU usage in order to enable large-scale analyses including orthology inference and clustering, which is a desirable goal in the field. The tool is written in Python and accessible on GitHub where it is also well documented. Overall, two new concepts are introduced to orthology prediction. First, an alternative algorithm for homology search which replaces the classical BLAST algorithm. Second, the application of the "Affinity Propagation Clustering algorithm" (APC) which replaces the well established but rather memory-intense MCL algorithm.

While the authors exemplify good prediction and recall characteristics compared to some other approaches, the principal argument of SwiftOrtho being a faster and more memory efficient tool than currently available is not convincingly demonstrated. There are already published orthology tools geared in the same direction. The few numbers presented on runtime and memory usage do not sound like a major improvement from my experience. Comparative benchmarks are missing in this respect. Similarly, the presentation of the current state of the field appears rather limited and the choice of alternative tools or example data sets is outdated. Overall, the provided data did not convince me.

Major issues:

1. Runtime and memory usage should be benchmarked not only versus OrthoMCL but also against more recent tools that cover homology search and clustering. I namely suggest SonicParanoid (cited but not benchmarked), Proteinortho6, and the standalone tool provided for OMA. Ideally, the two steps (homology search and clustering) could be evaluated separately.

Thank you for this comment. To clarify, we compared SwiftOrtho not only with OrthoMCL, but also with FastOrtho, Orthagogue, and Orthofinder. These results are shown in Tables 1-3 and Figures 3-6. As requested, We added the benchmark results of SonicParanoid, OMA, and ProteinOrtho6 on the same datasets used in this study. The results are found in Table S10. Currently, only SwiftOrtho can analyze the large Bac *set.*

2. BLAST is the golden standard in the field regarding sensitivity and specificity but not with respect to runtime or memory requirements. While I like the major concept, there are plenty of options besides the presented "built-in module" for homology search. It needs to be put into perspective. Ward, et al. (cited) for instance suggests BLAT, LAST, and UBLAST. Other promising BLAST-drop-ins are e.g. topaz and diamond. Besides, a loss of 24% of all RBHs when using the built-in BLAST alternative (line 238) is way more than what I would have expected from most of these other alternatives.

We used BLASTP as the orthology prediction tools we compared with specify

BLASTP as the default sequence alignment software. We require a homology search tool to: (1) not create a database on disk, which saves space; (2) be able to adapt to various application scenarios and flexibly switch between speed and sensitivity because it will be applied to our other projects; (3) be able to generate all the information we need so that it can be deeply integrated with our pipelines. Unfortunately, no software meets all these requirements, that's why we developed the "built-in module". We added the benchmark results of some fast BLAST-drop-ins, such as BLAT, LAST, UBLAST, Diamond, Topaz. The results are shown in Table S9.

3. Please use more recent tools and data sets. For instance, BLASTP v2.2.26 was published in 2012, which does not represent the current level of optimizations. The current version is 2.8.1 (2018). OrthoMCL is available at version 5 since 2011 (version 2 was used). Similarly, the QfO 2011 data set is deprecated since 2017.

1. *We have compared v2.2.27 and v2.8.1, they generate almost the same results in almost the same time. However, v2.8.1 was not stable on our computers, especially after turning on multi-threading, it often aborted. So, we still use v2.2.27 to test the software.*
2. *The Version 5 is the OrthoMCL DataBase not the standalone.The latest version of standalone is still at 2.0. (URL https://orthomcl.org/common/downloads/software/v2.0/)*
3. *The number of protein sequences in QfO 2018 dataset is twice the number of protein sequences in QfO 2011. In theory, the former will take nearly 4 more time and memory usage than the latter. In our tests, all-vs-all blastp search on QfO 2011 data set takes 7,000 cpu hours. If using QfO 2018, it will take ~28,000 CPU hours. In the interest of saving time, we chose QfO 2011 data set as a benchmark.*

4. For orthology inference, the data is stored somewhere in a sorted manner such that one "can process data that are much larger than the computer". Where is it stored? Temporary files on the hard drive? This is mentioned for the MCL clustering. How does your APC-implementation behave in this respect?

1. *The temporary files are stored on the hard drive. We have added this clarification in lines 128-129.*
2. *The matrices used in APC algorithm are also stored on hard drive. During updating cells, APC loads a submatrice into memory, which avoids loading all the data into memory and saves large amounts of memory. This is indicated in the manuscript in lines 156-158.*

5. The concepts of genomes, DNA sequences and amino acid sequences of proteins need to be sorted throughout the manuscript including the title. E.g. You did not analyze 1760 bacterial genomes but proteomes. Figure 2 shows nucleic acid alignments while the discussed context is about amino acids.

1. *We changed "genome" to "proteome" or some other indcation that proteins are analyzed, rather than genes. This is indicated in lines 175, 373, 428, 459, and*

caption of Table 2.

2. *Figure 2 shows the difference between standard Smith-Waterman and k-banded Smith-Waterman algorithm used in SwiftOrtho. The Smith-Waterman algorithm can be applied to protein sequences or nucleic acid sequences. To simplify the presentation, we used nucleic acid sequences to demonstrate the difference between two algorithms.*

6. The concepts of (co-)orthology and (in-)paralogy should be explained in some more detail. E.g. the likelihood to maintain the same biological function is much higher for orthologs than for paralogs. The term "in-paralog" should be explained with its first use and also put in context to out-paralogs.

1. *The term orthologs and paralogs are used to describe evolutionary relationships, not functional ones. The secondary use of orthologs and paralogs as proxies for understanding protein function is a derivative, and there are many documented cases where functional maintenance is more prevalent in paralogs than in orthologs (e.g. Nehrt et.al, PLoS-CB, 2011). Since we are dealing with the evolutionary classification, we limit ourselves to the evolutionary use of the terms.*
2. *We add more details about co-orthology and in/out-paralogs in background section.*

7. I did not quite get the spaced seed concept. Why would the user need/want to define at which position of a k-mer mismatches are allowed? This needs to be elaborated in more detail. I also find the respective binary vector given as parameter (-s) somewhat strange for a tool that is otherwise designed with easy use in mind (which I really appreciate). How much does it actually affect the results?

1. *The spaced seed used to improve the sensitivity of homology search. Usually, fast homology search tools are based on seed-and-extension method. Increasing seed length (k-mer size) increases search speed by reducing non-specific extension. However, long seed also reduces specific extension and results in low sensitivity. Spaced seed is one of the methods to increase the sensitivity, and has been used in many homology search tools, including PatternHunter, LAST, Diamond, usearch… In these software, PatternHunter and Usearch allows the users to set the pattern of spaced seed, LAST and Diamond don't allow users to change the spaced seeds. We have added wording to better describe this "Several tools such as PatternHunter, Usearch, LAST, and DIAMOND [19, 38, 40–42] have used spaced seed to increase sensitivity. PatternHunter and Usearch allow users to use custom spaced seed" in lines: 102-104.*
2. *SwiftOrtho has a default value for spaced seeds and the users do not need to change it, However, we still keep this option for experts.*
3. *The fast and sensitive mode of DIAMOND uses different combination spaced seeds, and the results from different mode are very different. More benchmark results can be found in references[29, 30].*

8. How is the e-value calculated for the "built-in module" (homology search)?

*E = D * m * n * 2^-S', where D is the size of database, m and n are the length of query and reference sequences, S' is the bit score. This is described in the manuscript in Supplementary Material Section 2.2.*

9. When comparing different algorithms, be fair and make sure to use the same or equivalent parameters (as far as this is possible). The OrthoFinder e-value threshold was 1e-3 while the other tools were used with 1e-5.

We didn't set the e-value threshold for OrthoFinder. In fact, OrthoFinder has no option to change the e-value threshold. We checked the source code of OrthoFinder and found its e-value threshold is hardcoded to to 1x10^-3.

10. One of the two major selling points is the APC algorithm. It seems, however, not to be set as the default algorithm. The user can choose between this and MCL. Manuscript and tool description could use some information on this topic (when to use what). Is there any parameter for APC similar to the inflation parameter for MCL? Until multi-threading is implemented for APC, it looks more like a low-memory backup while it appears to be a fully fletched clustering alternative. What about simply running the clustering of several connected groups in parallel? In times with plenty of CPU-cores, it is the overall runtime that counts rather the bare CPU time.

1. *Currently, most of the orthology prediction tools prefer MCL as a clustering algorithm, which is why we also recommend that users try the MCL algorithm first. However, since the MCL algorithm is an in-memory algorithm which loads all the data into memory, it cannot cluster data which size is larger than the system memory. The APC algorithm processes data line by line and doesn't have the memory issue. So,in cases of low memory constraints, the users can use APC algorithm for clustering.*
2. *As far as we know, APC has no similar parameter to the inflation parameter for MCL.*
3. *The bottleneck of our APC implementation is the I/O performance. Even when multi-threading, the APC still uses much real time. Due to its complexity, we do not support multithreading for APC now.*
4. *Running clustering of connected components may be a good method to parallelize APC algorithm. However, the size of connected components is highly diverse, for example, the largest connected component of the Bac set covers 90% of the edges, which makes parallelization meaningless, single thread must process 90% of data.*

11. How is the RBH implemented in SwiftOrtho? Usually, not only the single best hit is used but also very close ones. This might be an alternative reason for the lower number of orthology relationships (line 243).

We have described in detail how to implement the RBH method in Figures 1 and lines 126-130. SwiftOrtho uses a stringent RBH method to detect orthologs. For a gene X in genome A, SwiftOrtho only picks up top 1 hit of gene X in genome B. SwiftOrtho

may pick up multiple hits if and only if the top N hits has the same similarity or score.

12. The underlying data sets, as well as the results from which precision and recall scores were calculated, should be made available online. It is not trivial to reproduce e.g. the initial RBH graph from 1760 species which is necessary to evaluate the clustering algorithm. How was the data chosen?

The species was chosen if they have complete sequenced genomes. In order to reduce redundancy, we only randomly selected one strain from each species. Since this data set is not a standard benchmark data set for assessing precision and recall, we cannot directly calculate precision and recall. We can only evaluate whether the results are reasonable through indirect methods. In this study, we compared the results of SwiftOrtho and FastOrtho, and found that ~90% orthology relationships were shared by these tools (lines: 380-382).

13. I could hardly follow the APC formulas. Important details are missing, e.g. that matrices are initialized with 0. What is the difference between a well-suited node and an appropriate node (lines below line 131)? A calculation example would be useful. In general, a reference or proof is required for the algorithm and the assumptions made (e.g. line 132). APC requires less memory, what are other advantage and disadvantage in general?

1. *A well-suited node has higher "responsibility value" than an appropriate node, where "responsibility value" represents how "appropriate" it would be for point i to pick point k as its exemplar, taking into account other points' preference for poin k as an exemplar. For more details, please refer to reference[42].*
2. *Reference[42] has more details about APC algorithm.*
3. *(1). Advantage: APC requires much less memory and is fast. (2). Disadvantage: Vlasblom et al.(2009) compared MCL and APC on protein-protein interaction network and the results show that MCL is more tolerant to noise and behaves more robustly than the APC; In our practice, the APC is more sensitive to the metric methods (similarity matrix), the clustering result of normalized similarity matrix is very different from raw one.*

Minor issues:

- Why is there a need to perform large-scale orthology predictions with only 4 GB of memory? Even consumer PCs have at least 8 GB installed.

1. *SwiftOrtho was designed to run on the computers with various hardware specifications, including old computers. Researchers in low and middle-income countries may still benefit from such a system. However, a low-overhead system is generally beneficial to all. The 4GB example is* in-extremis, *but it illustrates that SwiftOrtho is a low-resource package.*
2. *If SwiftOrtho can perform large-scale orthology predictions on low-memory computer, it can analyze more data on a computer with more RAM.*

- Line 37: If I'm not mistaken, OrthoMCL does not use the Inparanoid algorithm even though its implementation behaves similarly when used with only two genomes.

The authors of OrthoMCL (Li Li et.al, 2003) mentioned that: "...This approach is similar to INPARANOID, but differs primarily in the requirement that recent paralogs must be more similar to each other than to any sequence from other species…". OrthoMCL uses the same algorithm as Inparanoid to detect orthologs, but uses a higher threshold to detect recent paralogs (in-paralogs).

- Line 345: It might be advisable to remove different protein isoforms from the data set beforehand. Usually, these are indicated. If in doubt, only the longest variant can be used.

We tried to remove isoforms. However, the gene identifiers of the sequences don't contains related information. It takes too much time and effort to use self-alignment method to remove isoforms. So, we just keep all sequences.

- As the BLAST+ implementation is used, its paper should be cited as well: Camacho C., Coulouris G., Avagyan V., Ma N., Papadopoulos J., Bealer K., & Madden T.L. (2008) "BLAST+: architecture and applications." BMC Bioinformatics 10:421.

We have now cited this paper, reference [57].

Notes:
- Users typically download a single fasta file for each species of interest from databases. For use with SwiftOrtho they are required to merge them to a single file and adding a taxon code. While this might be trivial for experienced users, it is an unnecessary obstacle for less versed users. Moreover, this strategy of an all-vs-all BLAST increases memory requirements over a species-by-species implementation. The many output files (discussed in the manuscript) could be merged.

SwiftOrtho's homology search module is different from other tools. It builds in-memory database after loading the fasta file. If we use species-by-species strategy, SwiftOrtho must build the database for each species several times, which make it much slower. We added a simple script to merge the fasta at github of SwiftOrtho project.
- line 33: "and and"
- line 102: ". if"
- line 401: sentence was not finished
- citation 11 contains an unnecessary long link

Thank you for these comments. We have now fixed these errors, and proofread the manuscript carefully.

- author contribution and acknowledgments are missing

Those have been added.

(Reviewer 3)

The authors present SwiftOrtho, a lightweight and modular homology detection and classification tool. They correctly state that the current tools available for homology searching often involve all-against-all pairwise comparisons, meaning the compute and RAM requirements can be large, and these analyses can take hours or days depending on the number of species and genes. The authors carry out a number of comparative analyses to demonstrate the efficacy of SwiftOrtho. They present evidence that their tool is comparable to others in terms of accuracy via various metrics, but accuracy is sacrificed to achieve very low RAM use to allow the tool to be run on low power computers. This is the key novelty of this application - being able to access these kinds of tools on a broader range of compute options is useful. The modularity of SwiftOrtho is beneficial to allow various homology analyses to be used in place of SwiftOrtho's homology module, which can help with longevity of the tool.

For the most part, the paper is well-written, has few grammatical and typography errors, and is structured so that the authors' processes are easily followed. The code and data used in the manuscript are freely available.

Specific comments:

The tool is written in Python 2.7. This version is due to be unsupported in January 2020. The tool should be updated within the next year to support Python 3.

We upgraded the source code to Python3

Whilst there are two main methods of ortholog identification (tree and graph), these are not mutually exclusive and some approaches use both (Ensembl Compara / GeneSeqToFamily for example). This should be stated in the manuscript.

We added a review of the hybrid method (lines: 44-50).

The authors do not present the rationale for using SonicParanoid and InParanoid only on the QfO 2011 dataset rather than all datasets, apart from using it as a control. This should be stated.

F.Chen et al (2007) compared several orthology prediction methods and found InParanoid and OrthoMCL outperformed other tools. InParanoid has the best performance from the QfO website. So, why we chose InParanoid as control. SonicParanoid is an faster but less sensitive C-implementation of InParanoid, we also added it as control. We added this rationale in lines: 234-238.

In the abstract, the authors use the word "costly" to describe computational clusters capable of carrying out these types of analysis. Whilst bigger HPC/HTC clusters do indeed make the job quicker, these do not necessarily have to be costly. Indeed, the

cost is never explored in the paper. I suggest removing the subjectivity by omitting "costly" in the abstract.

We removed the word “cost”.

The authors also state that upwards of 100GB RAM for OrthoAgogue to run "exceeds most workstations and servers". This isn't true for the current (March 2019) situation. Building a server 128GB RAM is fairly trivial these days, and can easily cost less than a 2019 MacBook. Similarly, renting a memory-optimised ~128GB server (e.g. r4.4xlarge VM instance type) from Amazon Web Services is easy. This sentence should be reworded to reflect that whilst laptops and desktops do not have this RAM availability, it is certainly not uncommon in "most servers".

We removed the wording that includes “most servers”(line 295).

General grammatical fixes(line numbers shown):

30: "and and"
31: "or COG" -> "(COG)"
46: "on large dataset" -> "on large datasets"
51: "large data set" -> "large datasets"
71: When describing the two sensitivity compensation mechanisms (line 71) it is inferred that the authors will describe both. Consider removing the "Another method" sentence, and merging it with the next, e.g. "Reduced amino acid alphabets are also used to mitigate the loss of sensitivity be representing...." etc.
114: "as edge-weighting" -> "as the edge-weighting"
115: "step take" -> "step takes"
115: remove colon and replace with full stop
126, 129: Both lines start with "However". Consider removing the repetition to make the comparison clearer (probably the second "however" isn't needed)
After 131: the paragraph "For the large networks" contains "node i" and "node k" that aren't italicised.

Thank you for these comments. We have now fixed these errors, and proofread the manuscript carefully.

401: Incomplete sentence: "be used to ..."

We removed this sentence.

Code repository and tests:

The software installs and runs as intended.

The author should update the readme file with the following corrections:
- Example commands are showing missing input file (we used ref.fsa from the example)
- Example command for find_hit.py is missing the "-p" parameter
- Installation instructions are missing "pip install cffi" command in readme file

Thanks for the report. We add an installation step and the installation script will find and install all the packages.

The author should add available options for any of the command line parameters (i.e. -p in find_hit.py) in the help message when running the tool.

Users can find a help message for each tool by typing "python foo.py", foo.py is one of find_hit.py, find_orth.py, and find_cluster.py.

Most of the times SwiftOrtho tool is unable to recognise paralogs when orthologs are detected. SwiftOrtho only recognises in-paralogs if the bit score between two paralogs is higher than orthologs. This might be covered by the use of the term "in-paralogs", but it's not explicitly clear from the manuscript. This should be addressed.

SwiftOrtho can detected orthologs and in-paralogs. We changed "paralogs"to "in-paralogs"in the manuscript.

We were not able to run SwiftOrtho successfully when supplying data for the gene BRAT1 from (chimp, human, pig, dog, and rat) containing within-species paralogs from chimp. In this case, find_orth.py script fails with the following error:

```
Traceback (most recent call last):
  File "../SwiftOrtho/bin/find_orth.py", line 551, in <module>
    st, ed, pairs = binary_search(Sqco, [qip, sip], lambda x: x.split('\t', 3)[:2])
  File "../SwiftOrtho/bin/find_orth.py", line 403, in binary_search
    left = m - 1
UnboundLocalError: local variable 'm' referenced before assignment
```

We are happy to raise an issue in Github for this issue if required.

Thank you for reporting this issue. Recently, We found a bug that if there is no orthologs found, SwiftOrtho reports the same error. We fixed it but we are not sure whether it works for your data set. You can clone the latest SwiftOrtho to test it again. If the problem persists, please file a github issue and send us your test file to help us solve the problem.
