# Supplementary material for: SwiftOrtho: A fast, memory-efficient, multiple genome orthology classifier
Source: Gigascience. 2019 Oct 24;8(10):giz118. doi: 10.1093/gigascience/giz118 (PMC6812468; doi:10.1093/gigascience/giz118)
Supplement: giz118_GIGA-D-19-00043_Revision_2 [file giz118_giga-d-19-00043_revision_2.pdf]

GigaScience

SwiftOrtho: a Fast, Memory-Efficient, Multiple Genome Orthology Classifier

--Manuscript Draft--

**Manuscript Number:**	GIGA-D-19-00043R2
**Full Title:**	SwiftOrtho: a Fast, Memory-Efficient, Multiple Genome Orthology Classifier
**Article Type:**	Technical Note
**Funding Information:**	Directorate for Biological Sciences (1854685) — Dr. Iddo Friedberg
**Abstract:**	Background : Gene homology type classification is required for many types of genome analyses, including comparative genomics, phylogenetics, and protein function annotation. Consequently, a large variety of tools have been developed to perform homology classification across genomes of different species. However, when applied to large genomic datasets, these tools require high memory and CPU usage, typically available only in computational clusters.<br><br>Findings: Here we present a new graph-based orthology analysis tool, SwiftOrtho, which is optimized for speed and memory usage when applied to large-scale data. SwiftOrtho uses long k -mers to speed up homology search, while using a reduced amino acid alphabet and spaced seeds to compensate for the loss of sensitivity due to long k -mers. In addition, it uses an Affinity Propagation algorithm to reduce the memory usage when clustering large-scale orthology relationships into orthologous groups. In our tests, SwiftOrtho is the only tool that completed orthology analysis of proteins from 1,760 bacterial genomes on a computer with only 4GB RAM. Using various standard orthology datasets, we also show that SwiftOrtho has a high accuracy.<br>Conclusion: SwiftOrtho enables the accurate comparative genomic analyses of thousands of genomes using low memory computers.<br><br>Availability: SwiftOrtho is available at: https://github.com/Rinoahu/SwiftOrtho
**Corresponding Author:**	Iddo Friedberg<br>Iowa State University<br>Ames, Iowa UNITED STATES
**Corresponding Author Secondary Information:**	
**Corresponding Author's Institution:**	Iowa State University
**Corresponding Author's Secondary Institution:**	
**First Author:**	Xiao Hu
**First Author Secondary Information:**	
**Order of Authors:**	Xiao Hu
	Iddo Friedberg
**Order of Authors Secondary Information:**	
**Response to Reviewers:**	Iddo Friedberg, PhD<br>Associate Professor<br>College of Veterinary Medicine<br>Iowa State University<br>Ames, IA 50011<br>T: +1 515 294 5959<br>E: idoerg@iastate.edu<br><br><br><br>Hans Zauner, PhD

Powered by Editorial Manager® and ProduXion Manager® from Aries Systems Corporation

Editor, GigaScience
Oxford University Press

Dear Dr. Zauner,

Enclosed please find our revised manuscript. We are pleased that our manuscript has been accepted, and We would like to thank the reviewers and yourself for your time and effort. The comments were minor, and addressed mostly style and typographical errors.

We are happy to include the paper in the Technical Notes section. We have registered SwiftOrtho in SciCrunch.org, and added the Software Availability section, and the availability of supporting source code and requirements. We have already worked with your staff and uploaded the data to the GigaScience data repository, and referenced that in our manuscript.

Below are the reviewers' requests, and our detailed responses. Note that we also attached a PDF with these responses.

Sincerely,

Iddo Friedberg

Reviewer 1

Overall, the comments have been addressed adequately.
I only have two remaining issues, which should be possible to address without further re-review:
- My comment on Figure 6D was apparently misunderstood. I was wondering why the y-axis needs to have a lower value below zero. Similarly, the point regarding OrthoFinder in Figure 6F indicated that visually the y-axis choice makes it look as if it were horrible, while in reality there is a ±10-20% difference.
- The documentation of the code in e.g. https://github.com/Rinoahu/SwiftOrtho/blob/master/bin/find_cluster.py could still be improved; several functions only have a one word explanation or use unclear abbreviations. Also, the widespread use of single-letter variable names make it very difficult to follow what is happening. I feel that one more round of revision would really help for future use and maintenance of the code, but I will leave this at the author's discretion.

We have now fixed the Figures to reflect the reviewer's requests. We have also redocumented the code, but have not made major changes to the variable names, etc. as that will require a new release of the software.

Reviewer 2

The authors addressed all major points raised in the first review. The inclusion of comparative benchmarks with other BLAST-like methods and orthology inference tools puts SwiftOrtho in a good perspective to other software, highlighting its advantages regarding low computational requirements to a so far unmet degree. An upgrade to Python 3 increases its durability in the near future.

Minor points:
- author contribution and acknowledgments are still missing
- line 40: As stated in the author's answer: "This approach is similar to InParanoid" The current phrasing in the manuscript implies that InParanoid is explicitly called by OrthoMCL which is not true.
- typos: line 127 (stores), line 414 (Proteinortho)

Powered by Editorial Manager® and ProduXion Manager® from Aries Systems Corporation

	We have now fixed those errors. Thank you for pointing them out.<br><br><br><br>Reviewer 3<br><br>The authors have addressed my concerns, and appear to have covered those of the co-reviewers. The extra supplemental information on the tool comparisons is welcome. I only have minor grammatical changes to pass on:<br><br>Lines 46-47: "first perform all-vs-all sequence alignment, then constructs gene families by the sequence similarity or conserved gene neighborhood" : "first performs all-vs-all sequence alignment, then constructs gene families by sequence similarity or conserved gene neighborhood"<br>L67-68: "However, these tools require high performance computers to analyze large-scale data." : "However, these tools tend to require high performance computers with large memory available to analyze large-scale data"<br>L102-103: "Spaced seed" should be plural in both cases.<br>L127: "First, SwiftOrtho sorts the data and store it on hard drive." : "Firstly, SwiftOrtho sorts the data and stores it on the filesystem"<br>L419: "[of, For] Bac set" : "[of, For] the Bac set"<br>L421: "impractical to apply OMA to large-scale data set." : "impractical to apply OMA to a large-scale data set in most computational environments"<br>L424: "Bac set" : "the Bac set"<br>L430-431: "on Bac set" : "on the Bac set"<br>L433: "in all-vs-all homology search step" : "in the all-vs-all homology search step"<br>L436: "of orthology inference" : "of the orthology inference"<br>L437: "to Bac set" : "to the Bac set"<br>L440-441: "In sum, these results show that SwiftOrtho is a top performer on large-scale data." : "In summary, these results show that SwiftOrtho is the most computationally efficient on large-scale data."<br><br>Thank you for your diligence. We have now fixed these errors.
**Additional Information:**	
**Question**	**Response**
Are you submitting this manuscript to a special series or article collection?	No
**Experimental design and statistics**<br><br>Full details of the experimental design and statistical methods used should be given in the Methods section, as detailed in our Minimum Standards Reporting Checklist. Information essential to interpreting the data presented should be made available in the figure legends.<br><br>Have you included all the information requested in your manuscript?	Yes
**Resources**	Yes

Powered by Editorial Manager® and ProduXion Manager® from Aries Systems Corporation

A description of all resources used, including antibodies, cell lines, animals and software tools, with enough information to allow them to be uniquely identified, should be included in the Methods section. Authors are strongly encouraged to cite Research Resource Identifiers (RRIDs) for antibodies, model organisms and tools, where possible.<br><br>Have you included the information requested as detailed in our Minimum Standards Reporting Checklist?	
**Availability of data and materials**<br><br>All datasets and code on which the conclusions of the paper rely must be either included in your submission or deposited in publicly available repositories (where available and ethically appropriate), referencing such data using a unique identifier in the references and in the “Availability of Data and Materials” section of your manuscript.<br><br>Have you have met the above requirement as detailed in our Minimum Standards Reporting Checklist?	Yes

Powered by Editorial Manager® and ProduXion Manager® from Aries Systems Corporation

Manuscript
Click here to view linked References

GigaScience, 2017, 1–9
Click here to access/download;Manuscript;main.dvi

doi: xx.xxxx/xxxx
Manuscript in Preparation
Paper

PAPER

SwiftOrtho: a Fast, Memory-Efficient, Multiple Genome Orthology Classifier

Xiao Hu[1,2]* and Iddo Friedberg[1]*

[1]Department of Veterinary Microbiology and Preventive Medicine, College of Veterinary Medicine, Iowa State University, Ames Iowa USA and [2]Present address: Gianforte School of Computing, Montana State University, Bozeman, Montana, USA

*xiao.hu1@montana.edu; idoerg@iastate.edu

Abstract

Background: Gene homology type classification is required for many types of genome analyses, including comparative genomics, phylogenetics, and protein function annotation. Consequently, a large variety of tools have been developed to perform homology classification across genomes of different species. However, when applied to large genomic datasets, these tools require high memory and CPU usage, typically available only in computational clusters.
Findings: Here we present a new graph-based orthology analysis tool, SwiftOrtho, which is optimized for speed and memory usage when applied to large-scale data. SwiftOrtho uses long k-mers to speed up homology search, while using a reduced amino acid alphabet and spaced seeds to compensate for the loss of sensitivity due to long k-mers. In addition, it uses an Affinity Propagation algorithm to reduce the memory usage when clustering large-scale orthology relationships into orthologous groups. In our tests, SwiftOrtho is the only tool that completed orthology analysis of proteins from 1,760 bacterial genomes on a computer with only 4GB RAM. Using various standard orthology datasets, we also show that SwiftOrtho has a high accuracy.
Conclusion: SwiftOrtho enables the accurate comparative genomic analyses of thousands of genomes using low memory computers.
Availability: SwiftOrtho is available at: https://github.com/Rinoahu/SwiftOrtho

Key words: Orthology Analysis; Homology Search; Orthology Inference; Clustering; Orthologs; Paralogs

Background

Gene homology type classification consists of identifying paralogs and orthologs across species. Orthologs are genes that evolved from a common ancestral gene following speciation, while paralogs are genes that are homologous due to duplication. Paralogs can be further classified into in-paralogs, which evolved via gene duplication before the speciation event, and out-paralogs, which evolved via gene duplication after the speciation event [1]. Classifying orthologs and paralogs across species is an important problem, as the evolutionary history of genes has implications for our understanding of gene function and evolution.

While the proper inference of homology type involves tracing gene history using phylogenetic trees [2], several proxy methods have been developed over the years. The most common method to infer orthologs by proxy is Reciprocal Best Hits or RBH [3, 4]. Briefly, RBH states the following: when two proteins that are encoded by two genes, each in a different genome, find each other as the best scoring match among all homologs, they are considered to be orthologs [3, 4].

InParanoid extends the RBH orthology relationship to include both orthologs and in-paralogs. Specifically, InParanoid uses RBH to identify orthologs between two species. The genes in the two species are classified as in-paralogs if they are more similar to the corresponding ortholog than to any gene in the other species [5, 6, 7]. The concept of orthologous pairs between two species can be extended to an *ortholog group*, which is a set of genes that are hypothesized to have descended from a common ancestor [7]. Several methods have been developed to identify ortholog groups across multiple species typically classified as either tree-based or graph-based methods. Tree-based methods construct a gene tree from an alignment of homologous sequences in different species and infer orthology relationships by reconciling the gene tree with its corresponding species

Compiled on: August 30, 2019.
Draft manuscript prepared by the author.

tree [2, 8, 9], and can infer a correct orthology relationship if the correct gene tree and species tree are provided [10]. The chief limiting factor of tree-based methods is the accuracy of the given gene tree and species tree. Erroneous trees lead to incorrect ortholog and in-paralog assignments [11, 10, 9]. Tree-based methods are also computationally expensive which limits the ability to apply them to large number of species [12, 10, 13, 14]. Graph-based methods infer orthologs and in-paralogs from homologs and then use different strategies to cluster them into orthologous groups [12, 9, 13] (Figure 1). The Clusters of Orthologous Groups (COG) database detects triangles of RBHs in three different species and merges the triangles with a common side [15]. Orthologous Matrix (OMA) clusters RBHs in orthologous groups by finding maximum weight cliques from the similarity graph [16, 17]. MultiParanoid is an extension of InParanoid, which uses InParanoid to detect triangle orthologs and in-paralogs in three different species as seeds and then merges the seeds into larger groups [18]. OrthoMCL also uses InParanoid to detect orthologs, co-orthologs, and in-paralogs between two species [19, 20] and then uses Markov Clustering (MCL) [21] to cluster these relationships into orthologous groups, where the co-orthologs are two or more genes in one species that are orthologous to one or more genes in another species due to a gene duplication event [22, 1].

Finally, there are hybrid methods that combine both graph-based and tree-based methods [12, 23, 24, 25, 26]. Typically, hybrid methods first perform all-*vs*-all sequence alignment, then construct gene families by sequence similarity or conserved gene neighborhood. EnsEMBL first uses RBH to find the gene families, then constructs a phylogenetic gene tree for each gene family [24]. Finally, each gene tree is reconciled with the species tree to infer paralogs and orthologs.

In theory, graph-based methods are less accurate than tree-based methods, as the former identify orthologs and in-paralogs using proxy methods rather than directly inferring homology type from gene and species evolutionary history. However, graph-based methods have been found to be comparably accurate to tree-based methods [11, 10, 27]. Moreover, a comparison of several methods found that tree-based methods had even a worse performance than graph-based methods on large datasets [11].

One study compared several common methods, including simple RBH, graph-based, tree-based, and hybrid methods, and found that the tree-based methods of InParanoid and OrthoMCL exhibit the best balance of sensitivity and specificity [28]. Several studies have also shown that graph-based methods find a better trade-off between specificity and sensitivity than tree-based methods [11, 28, 29]. For these reasons, graph-based methods are generally preferred for analyzing large-scale data sets. OrthoMCL and InParanoid have been applied to analyze hundreds of genomes; at the same time, they require considerable computational resources that may not be readily available [20, 30]. More recently, several graph-based tools, such as SonicParanoid, OMA, and ProteinOrtho [31, 17, 32] have been developed to speed up orthology analysis on large-scale data sets. There tools also tend to require high performance computers with large memory to analyze large-scale data.

Here we present SwiftOrtho, a fast method for orthology classification that makes minimal use of computational resources, especially memory. SwiftOrtho uses a seed-and-extension method to speed up homology search, a binary search method and RBH rule to infer orthologs and in-paralogs, and the Affinity Propagation algorithm to reduce memory usage in cluster analysis. We compare SwiftOrtho with several existing graph-based tools using the gold standard dataset Orthobench [13], and the Quest for Orthologs service [33]. Using both benchmarks, we show that SwiftOrtho provides a high accuracy with lower CPU and memory usage than other graph-based methods. SwiftOrtho is the only tool that completed an orthology analysis of 1,760 bacterial genomes on very a low-memory computer. With the growing number of genomes, especially microbial genomes, we see SwiftOrtho to be a tool of choice for a fast and accurate ortholog classification, while requiring low computational resources, as are found in conventional desktop or laptop computers.

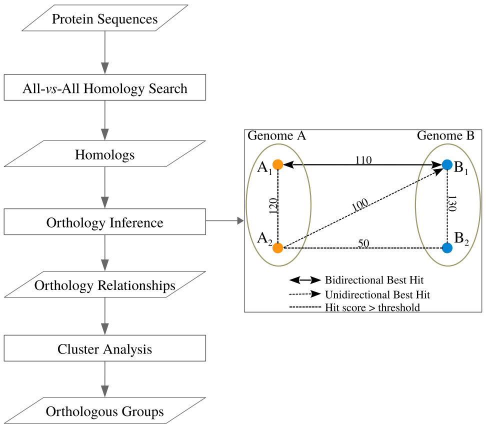

Figure 1. The flow cart of SwiftOrtho. SwiftOrtho is a graph-based method which consist of three major steps: **All-*vs*-All Homology Search**: A seed-and-extension method is used to perform homology search; **Orthology Inference:** Nodes are gene names, edges are similarity score of pairwise genes. 1. A_1-B_1 are putative orthologs identified by RBH. 2. A_1-A_2 and B_1-B_2 are putative in-paralogs as the bit scores of these pairs greater than A_1-B_1; 3. A_2-B_1 and A_2-B_2 are putative co-orthologs as these pairs are not orthologs but A_1-B_1 are orthologs and A_1-A_2, B_1-B_2 are in-paralogs; **Cluster Analysis:** Markov clustering or Affinity Propagation Algorithm is used to cluster orthology relationships.

Application of SwiftOrtho

Data Sets

We applied SwiftOrtho to three data sets to evaluate its predictive quality and performance:

i. The *Euk* set was used to evaluate the quality of predicted orthologous groups. This set contains 420,415 protein sequences from 12 eukaryotic species, including *Caenorhabditis elegans, Drosophila melanogaster, Ciona intestinalis, Danio rerio, Tetraodon nigroviridis, Gallus gallus, Monodelphis domestica, Mus musculus, Rattus norvegicus, Canis familiaris, Pan troglodytes* and *Homo sapiens*. The protein sequences for these genes were downloaded from EMBL v65 [34].
ii. The *QfO 2011* set was used to evaluate the quality of predicted orthology relationships. This set was the reference proteome dataset (2011) of The Quest for Orthologs[33], which contains 754,149 protein sequences of 66 species.
iii. The large *Bac* set was used to evaluate performance, including CPU time, real time and RAM usage. This set includes 5,950,817 protein sequences from 1,760 bacterial species. The protein sequences were downloaded from GenBank [35]. For a full list, see the additional file 1.

We also compared SwiftOrtho with several existing orthology analysis tools for predictive quality and performance. The methods compared were: OrthoMCL(v2.0), FastOrtho, OrthAgogue, and OrthoFinder.

Orthology Analysis Pipeline

The pipeline for all the tools follows the standard steps of graph-based orthology prediction, (1) all-*vs*-all homology search, (2) orthology inference, and (3) cluster analysis.

Homology Search

SwiftOrtho used its built-in module to perform all-*vs*-all homology search. For all the three sets, the E-value was set 10^{-5}. The amino acid alphabet was set to the regular 20 amino acids for the three sets. The spaced seed parameter was set to `1011111,11111` for the `Euk`, `11111111` for the *QfO 2011*, and `111111` for *Bac*.

OrthoMCL, FastOrtho, OrthAgogue, and OrthoFinder use BLASTP (v2.2.27+) [36] to perform all-*vs*-all homology search. The first three tools require the user to do this manually. To compare the methods, the -e (e-value), -v (number of database sequences to show one-line descriptions), and -b (number of database sequence to show alignments) parameters of BLASTP were set to 10^{-5}, 1,000,000, and, 1,000,000 for OrthoMCL, FastOrtho, and OrthAgogue. The OrthoFinder calls BLASTP, and the E-value of BLASTP have been set to 10^{-3}.

Orthology Inference

SwiftOrtho, OrthoMCL, FastOrtho, OrthAgogue, and OrthoFinder were applied to perform orthology inference on the homologs. The first four tools are able to identify (co-)orthologs and in-paralogs, and the coverage (fraction of aligned regions) was set to 50%, while other parameters were set to their default values, see Supplementary Materials for full details.

FastOrtho does not report (co-)orthologs and in-paralogs directly. However, the relevant information is stored in an intermediate file, from which we have extracted that information. Orthofinder does not report orthology relationships.

Cluster Analysis

All the tools in this study use MCL [21] for clustering. To control the granularity of the clustering, MCL performs an inflation operation set by the *-I* option [21, 37]. In this study, *-I* was set to 1.5. To take advantage of multiprocessor capabilities, we set the thread number of MCL to 12. SwiftOrtho has an alternative clustering algorithm APC, which we have also applied to *Euk* and*Bac*.

Evaluation of Prediction Quality

Evaluation of Predicted Orthologous Groups

The OrthoBench set was used to evaluate the quality of predicted orthologous groups in *Bac*. This set contains 70 manually curated orthologous groups of the 12 species from *Bac* and has been used as a high quality gold standard benchmark set for orthologous group prediction [13]. We used OrthoBench v2 (Supplementary Table S1). Each manually curated group of OrthoBench v2 set finds the best match in the predicted orthologous groups, where the best match means that the number of genes shared between manually curated and predicted orthologs is maximized, and the method to calculate precision and recall is shown in Supplementary Figure S1.

Evaluation of Predicted Orthology Relationships

The *Quest of Orthologs* web-based service (QfO) was employed to evaluate the quality of the orthology relationships predicted from the *QfO 2011* set[33]. The QfO service evaluates the predictive quality by performing four phylogeny-based tests of *Species Tree Discordance Benchmark*, *Generalized Species Tree Discordance Benchmark*, *Agreement with Reference Gene Phylogenies: SwissTree*, and *Agreement with Reference Gene Phylogenies: TreeFam-A*, and two function-based tests of *Gene Ontology conservation test* and *Enzyme Classification conservation test* [33].

We also applied two more orthology prediction tools, SonicParanoid[31] and InParanoid (v4.1)[5], on the *QfO 2011* set and used their results as control because InParanoid has best performance among the results from QfO service website and SonicParanoid is a fast implementation of InParanoid. The pairwise orthology relationships were extracted from the predicted orthologous groups of all the tools, including SonicParanoid and InParanoid, and then submitted to the QfO web-service for further evaluation.

Hardware

Unless specified otherwise, all tests were run on the Condo cluster of Iowa State University with Intel Xeon E5-2640 v3 at 2.60GHz, 128GB RAM, 28TB free disk. The Linux command `time -v` was used to track CPU and peak memory usage.

Findings

We compared the orthology analysis performance of SwiftOrtho, OrthoMCL, FastOrtho, OrthAgogue, and OrthFinder using *Euk*, *QfO 2011*, and *Bac*. The orthology analysis consists of homology search, orthology inference, and cluster analysis.

Orthology Analysis on *Euk*

The results of orthology analysis on *Euk* are summarized in Table 1, and are elaborated upon below.

		SwiftOrtho		OrthoMCL	FastOrtho	OrthAgogue	OrthoFinder
**Homology Search**	Method	SO built-in		BLASTP			
	Hits	162,695,330		947,203,546			654,792,861
	Uniq Hits	162,695,330		297,107,872			266,104,611
**Orthology Inference**	(Co-)orthologs	1,422,920		8,279,424	3,297,613	1,265,553	N/A
	In-paralogs	631,033		2,517,166	2,546,296	759,989	N/A
**Clustering**	Algorithm	MCL	APC	MCL			
	Orthologous Groups	44,551	38,748	36,901	40,943	51,297	19,904

Table 1. Comparative orthology analysis on the *Euk* set. N/A: not available, SO: SwiftOrtho, MCL: Markov Clustering, APC: Affinity Propagation Cluster.

Homology Search

The homology search results show that BLASTP detected the largest number of homologs, 947,203,546. SwiftOrtho found 57.5% of the homologs detected by BLASTP but was 38.7 times faster than BLASTP. SwiftOrtho used longer *k*-mers, which reduced both specific and non-specific seed extension. The longer *k*-mers cause seed-and-extension methods to ignore sequences with low similarity. According to the RBH rule, orthologs should have higher similarity than non-orthologs, so, the decrease in homolgs of SwiftOrtho does not significantly affect the next orthology inference.

We compared RBHs inferred from homologs detected by BLASTP and SwiftOrtho, and the numbers of RBHs for BLASTP and SwiftOrtho are 899,473 and 957,387, respectively. Identical RBHs are 767,884 (85.37% of BLASTP). These results show that although SwiftOrtho found fewer homologs than BLASTP, it does not significantly reduce the number of RBHs. The following results in Figure 3 also show that there is no significant difference between SwiftOrtho and BLASTP in predicting orthologous groups. Homology searches against a large number of protein sequences are a major bottleneck in bioinformatics pipelines. For that reason, many tools have been developed to speed up this process including, among others, BLAT, Usearch, LAST, DIAMOND, and Topaz [38, 39, 40, 41, 42]. All these tools use longer k-mers than BLASTP to speed up performance. We also compared SwiftOrtho with them in speed and sensitivity, (Supplementary Table S9). Because BLASTP is widely considered the gold standard for comparing protein sequences, we use its results as the benchmark to evaluate the sensitivity of other homology search tools. We found Usearch and LAST to be the fastest, however, they only found 0.88% and 2.97% hits of BLASTP, respectively. Topaz and BLAT used the most CPU time, but found only 33.48% and 28.34% of the BLASTP hits, respectively. SwiftOrtho and DIAMOND (more sensitive mode) have

the highest sensitivity and found 52.72% and 58.30% of the BLASTP hits in a moderate amount of time, respectively. These results show that SwiftOrtho delivers a good trade-off between speed and sensitivity.

Orthology Inference

OrthoMCL and FastOrtho found more orthology relationships than SwiftOrtho and OrthAgogue. This is because OrthoMCL and FastOrtho use the negative log ratio of the e-value as the edge-weighting metric. The BLASTP program rounds E-value $< 10^{-180}$ to 0. Consequently, for homolgs with an e-value $< 10^{-180}$, OrthoMCL and FastOrtho treat them as the RBHs, overestimating the number of orthologs. An example showing the OrthoMCL and FastOrtho overestimation can be found in Table S4.

Use of Computational Resources

OrthoMCL v2.0 used the most CPU time and real time because of the required I/O operations. The RAM usage of OrthoMCL was 3.45GB, while the generated intermediate file occupied >19 TB of disk space. OrthAgogue was the most efficient in real time, because of its ability to exploit a multi-core processor. However, the RAM usage of OrthAgogue was more than 100GB which exceeds that of common workstations and many servers. The orthology inference module of FastOrtho was the most memory-efficient among all the tools and was also fast. SwiftOrtho was the most CPU time efficient, although its real time was twice as that of OrthAgogue. Because the orthology inference module of SwiftOrtho was written in pure Python, we retested it by using the PyPy interpreter, an alternate implementation of Python [43]. When running with PyPy the real run time of SwiftOrtho was close to that of OrthAgogue (Table S5)

Cluster Analysis

OrthoFinder identified the smallest number of orthologous groups. Other tools identified many more orthologous groups than OrthoFinder, ranging from 36,901 to 51,297. The APC algorithm found fewer clusters than the MCL algorithm.

Evaluation of Predicted Orthologous Groups

The quality of predicted orthologous groups is shown in Figure 2. OrthoFinder has the best recall, while SwiftOrtho and OrthAgogue have top precision values but lower recall values than other tools. Since SwiftOrtho and OrthAgogue use a more stringent standard to perform orthology inference, this strategy often increases precision but decreases recall [11, 28, 29].

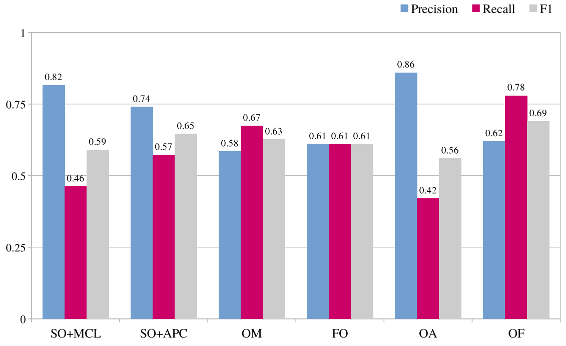

Figure 2. Evaluation of predicted orthologous groups. Evaluation of different tools on OrthoBench database. **SO+MCL**: SwiftOrtho with MCL; **SO+APC**: SwiftOrtho with Affinity Propagation Clustering; **OM**: OrthoMCL v2; **FO**: FastOrtho; **OA**: OrthAgogue; **OF**: OrthoFinder.

Because SwiftOrtho uses its built-in homology search module and its recall is lower than BLASTP's, it may reduce the recall of orthologous groups. To address this problem, we made two replacements. We replaced SwiftOrtho's homology module with BLASTP for SwiftOrtho and replaced BLASTP with SwiftOrtho's homology module for OrthoMCL, FastOrtho, OrthAgogue, and OrthoFinder. We then reran the orthology analysis on *Euk*. The results show that for most tools, replacing BLASTP with SwiftOrtho's built-in homology search module does not significantly reduce the recall (Figure 3). The difference in recall between using SwiftOrtho's homology search and using BLASTP is less than 4% except for OrthoMCL and FastOrtho. The recall for OrthoMCL and FastOrtho decreased by 8% and 7%, respectively. The most likely reason is that the E-value of SwiftOrtho's homology search module is more precise than that of BLASTP, which reduces the false RBHs as mentioned above. These results show that SwiftOrtho's homology search module is a reliable and fast alternative to BLASTP.

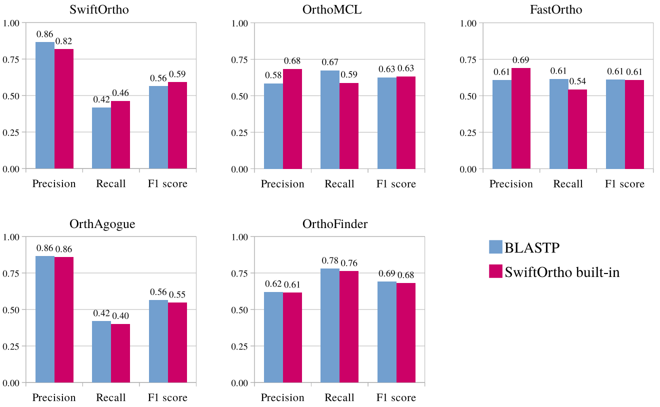

Figure 3. Comparing BLASTP and SwiftOrtho's homology search module on the quality of orthologous groups prediction. BLASTP and SwiftOrtho's search module perform an all-*vs*-all search on the *Euk* set, respectively. Then, all the orthology prediction tools were employed for orthology inference. Finally, the predicted orthology relationships were clustered into orthologous groups by MCL algorithm.

To test the differences exhibited by the clustering component of SwiftOrtho, we ran SwiftOrtho with MCL and APC on the same data. The results (Figure 4) show that performance of APC is close to that of MCL. APC improves the recall of most tools (Figure 4). These results show that APC has the similar performance as the MCL algorithm and is a reliable alternative to MCL.

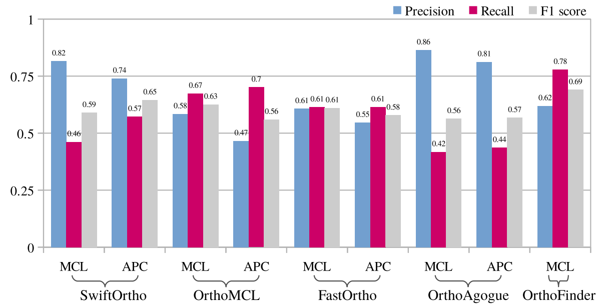

Figure 4. Markov Clustering versus Affinity Propagation Clustering. Both algorithms were applied to cluster the orthology relationships of the *Euk* set inferred by different orthology prediction tools, into orthologous groups. As OrthoFinder does not report orthology relationships, the Affinity Propagation can not be applied to its results. **MCL:** Markov Clustering algorithm; **APC:** Affinity Propagation Clustering.

Orthology Analysis on *QfO 2011*

The results of the orthology analysis on *QfO 2011* are shown in Table 2 and elaborated below.

		SwiftOrtho	OrthoMCL	FastOrtho	OrthAgogue	OrthoFinder
**Homology Search**	Method	SO built-in	BLASTP			
	Hits	183,883,417	642,372,369			935,579,809
	Uniq Hits	183,883,417	317,333,885			462,876,579
**Orthology Inference**	(Co-)orthologs	2,209,243	3,743,779	2,588,851	2,716,128	N/A
	In-paralogs	6,929,058	11,427,118	13,649,582	13,694,208	N/A
**Clustering**	Algorithm	MCL				
	Orthologous Groups	60,418	50,970	55,530	50,203	166,217

Table 2. Comparative orthology analysis on the Quest for Orthologs reference proteome 2011 dataset. SO: SwiftOrtho; MCL: Markov Clustering; APC: Affinity Propagation Cluster; N/A: not available.

Homology Search

SwiftOrtho found 183,883,417 unique hits while BLASTP found 462,876,579 unique hits. However, SwiftOrtho is about 163 times faster than BLASTP.

Orthology Inference

OrthoMCL found many more orthologs and co-orthologs than the other tools. SwiftOrtho found fewer in-paralogs than other available tools. The CPU time of SwiftOrtho is the least of all tools. When using the PyPy interpreter, the real time of SwiftOrtho is also close to that of the fastest one, OrthAgogue (Supplementary Table S6).

Cluster Analysis

Overall, the clustering numbers of SwiftOrtho, OrthoMCL, FastOrtho, and OrthAgogue are similar. However, the number of clusters found by OrthoFinder is three times that of other tools, and the next evaluation also shows that OrthoFinder performed poorly on *QfO 2011*.

Evaluation of Predicted Ortholog Relationships

The evaluation shows that the performance of SwiftOrtho is close to that of InParanoid (Figure 5). In some tests (Figure 5, D-E), SwiftOrtho outperformed InParanoid. SwiftOrtho had the best performance in the Generalized Species Tree Discordance Benchmark and Agreement with Reference Gene Phylogenies: TreeFam-A tests. In the Species Tree Discordance Benchmark, SwiftOrtho had the minimum Robinson-Foulds distance. In the Enzyme Classification (EC) conservation test, SwiftOrtho had the maximum Schlicker similarity. These two metrics reflect the accuracy of the algorithm, and the results show that SwiftOrtho has an overall higher accuracy than the other tools. At the same time, the recall of SwiftOrtho was lower in some of the QfO tests, the main reason is that SwiftOrtho uses an stringent metric system to identify orthology relationships.

Orthology Analysis On *Bac*

The results of orthology analysis on *Bac* are summarized in Table 3.

		SwiftOrtho		OrthoMCL	FastOrtho	OrthAgogue	OrthoFinder
**Homology Search**	Method	SO built-in					N/A
	Hits	8,478,732,753					N/A
	Uniq Hits	8,478,732,753					N/A
**Orthology Inference**	(Co-)orthologs	876,766,940		N/A	950,683,849	N/A	N/A
	In-paralogs	622,292		N/A	663,052	N/A	N/A
**Clustering**	Algorithm	MCL	APC	MCL			
	Orthologous Groups	240,162	167,355	N/A	242,816	N/A	N/A

Table 3. Comparative orthology analysis on the *Bac* set. SO: SwiftOrtho; MCL: Markov Clustering; APC: Affinity Propagation Cluster;
N/A: not available.

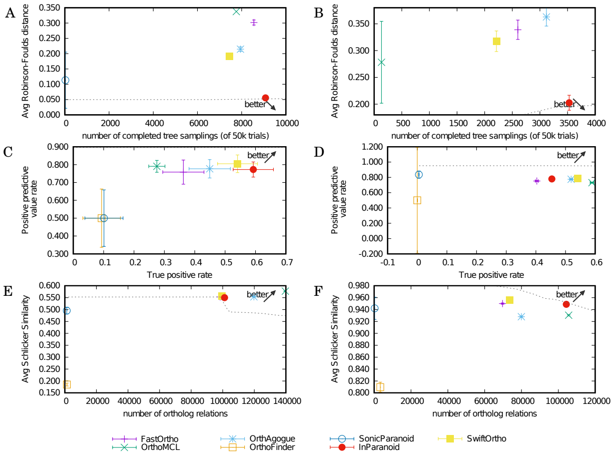

Figure 5. The Benchmarking in Quest for Orthologs. A: Species Tree Discordance Benchmark. InParanoid has minimum average Robinson-Foulds distance. SwiftOrtho's average RF distance is close to that of InParanoid. The prediction inferred by OrthoFinder is not available for this test; **B:** Generalized Species Tree Discordance Benchmark. InParanoid has minimum average Robinson-Foulds distance. The prediction inferred by OrthoFinder is not available for this test; **C:** Agreement with the Reference Gene Phylogenies of SwissTree. SwiftOrtho has the highest positive prediction value rate (recall). InParanoid has the highest true positive rate (precision); **D:** Agreement with Reference Gene Phylogenies of TreeFam-A. SonicParanoid has the highest positive prediction value rate (recall), however, its true positive rate (precision) is close to zero. SwiftOrtho has the second highest recall and precision; **E:** Gene Ontology conservation test. OrthoMCL has the highest average Schlicker similarity; **F:** Enzyme Classification conservation test. SwiftOrtho has the highest average Schlicker similarity. OrthoMCL detected the most orthology relationships and has the highest recall.

Homology Search

SwiftOrtho detected 8,966,131,536 homologs in the *Bac* set within 1,247 CPU hours.

Because it takes long time to perform all-*vs*-all BLASTP search on the full *Bac*, we randomly selected 1,000 protein sequences from *Bac* and used them to search against the full *Bac* set. It took BLASTP 5.1 CPU hours to find the homologs of these 1,000 protein sequences. We infer that the estimated CPU time of BLASTP on the full *Bac* set should be around 30,000 CPU hours. SwiftOrtho was almost 25 times faster than BLASTP on *Bac*.

Orthology Inference

SwiftOrtho, OrthoMCL, FastOrtho, and OrthAgogue were used to infer (co-)orthologs and in-paralogs from the homologs detected by the homology search module of SwiftOrtho in the *Bac* set. We did not test Orthofinder, because Orthofinder does not accept a single file of homologs as input. For the 1,760 proteomes in *Bac*, OrthoFinder needs to perform 3,097,600 pairwise species-by-species comparisons, which will generate the same number of files. Then, OrthoFinder performs the orthology inference on these 3,097,600 files. Even at one minute per file, it will take an estimated 6 CPU years to process all the files.

Due to memory limitations, only SwiftOrtho and FastOrtho finished the orthology inference on *Bac*. The results are shown in Table 3. The numbers of (co-)orthologs and in-paralogs inferred by SwiftOrtho and FastOrtho are similar. The number of common orthology relationships between SwiftOrtho and FastOrtho was 861,619,519 (98.2% of SwiftOrtho and 90.57% of FastOrtho). Compared with *Euk*, SwiftOrtho and FastOrtho have a similar predictive quality on *Bac*. There are three possible explainations for these results. The first is that *Euk* contains many protein isoforms which cause FastOrtho to overestimate the number of orthologs and in-paralogs. The second is that the gene duplication rate in Bacteria is lower than that in Eukaryotes [44, 45]. For *Bac*, each gene in one species has only small number of homologs in other species, which

makes FastOrtho unlikely to overestimate the number of RBHs. The third is that SwiftOrtho uses double-precision floating-point to store the E-value, which increases the precision of E-value from 10^{-180} to 10^{-308}. This improvement also reduces the possibility that FastOrtho may report false RBHs.

Computational resource use: Of the programs tested, only SwiftOrtho and FastOrtho finished the orthology inference step. FastOrtho and OrthAgogue did not finish the tests due to insufficient RAM; OrthoMCL aborted after running out of disk space, as it needed more than 18TB. The peak RAM usage of SwiftOrtho and FastOrtho were 90.6GB and 99.5GB, respectively. When we used the PyPy interpreter, the Peak RAM usage of SwiftOrtho was reduced to 72.1GB. FastOrtho was about 1.52 times faster than SwiftOrtho which ran the tests in the CPython interpreter. When using the PyPy interpreter, SwiftOrtho ran 1.58 times faster than FastOrtho. The memory usage and CPU time are shown in Table S7.

Cluster Analysis

The clustering numbers of SwiftOrtho and FastOrtho are similar. We compared the APC algorithm and the MCL algorithm, and APC found fewer clusters than MCL. The APC used much less memory and less CPU time than MCL. However, due to the lack of support for multi-threading and a large number of I/O operations, the real run time of APC is longer than that of MCL.

Tests on a Low-memory System

Because SwiftOrtho is designed to process large-scale data on low-memory computers, we used it to analyze *Bac* on a range of computers with different specifications.

The results show that the memory usage of SwiftOrtho is flexible and adapts to the size of the computer's memory. In the tests, SwiftOrtho finished an orthology analysis of *Bac* set on a computer with only 4GB RAM in a reasonable time (Table S8).

Comparison with other Orthology Analysis Pipelines

SonicParanoid, OMA, and ProteinOrth are also graph-based methods and have been optimized for large-scale data sets [31, 17, 32]. We compared SwiftOrtho with these tools in both speed and memory usage. The results are shown in Table S10. OMA appears to be the slowest as it it uses the Smith-Waterman algorithm to perform all-*vs*-all alignment. In our tests, OMA took 0.84 CPU hours to align two species (4,064 and 4,140 genes) of the *Bac* set. For the *Bac* set, OMA needs to perform 3,097,600 species-by-species alignments and the total time will be over two million CPU hours. SonicParanoid worked well on *Euk* and *QfO 2011* sets. Compared with SwiftOrtho, SonicParanoid ran faster and required less RAM on small data sets. However, it exited abnormally when applied to large *Bac* set. Proteinortho also worked well on the *Euk* and *QfO 2011* sets. When applied to the *Bac* set, Proteinortho needed to perform 1,547,920 species-by-species proteome alignments. It took Proteinortho 186.5 CPU hours, using DIAMOND, to complete 23,331 (1.5%) alignments; we therefore estimate that Proteinortho will take about 12,355 CPU hours to finish a full homology search. Since LAST is much faster than DIAMOND, we reran Proteinortho on the *Bac* set, using LAST for homology search. The CPU time for LAST on the *Bac* set was 2,368 hours. Although the previous results (Supplementary Table S9) show that LAST is about 20 times faster than SwiftOrtho, LAST required much more CPU time than SwiftOrtho in the all-*vs*-all homology search step. We think it is because the species-by-species alignment approach requires more than 1.5 million I/O operations, which significantly reduces the speed. The CPU utilization of orthology inference and clustering of Proteinortho was very low (less than 10%) when applied to the *Bac* set, which led to an exceptionally long real time run (more than 150 hours). The reason for this exceptionally long run time is because Proteinortho occupied about 85% of physical memory when applied to large-scale data, which resulted in frequent data exchange between RAM and swap space and greatly reduced the speed. In sum, these results show that SwiftOrtho is a top performer on large-scale data.

Discussion

We present SwiftOrtho, a new high performance graph based homology classification tool. Unlike most tools that only perform orthology inference, SwiftOrtho integrates all the modules necessary for a full orthology analysis, including homology search, orthology inference, and cluster analysis. SwiftOrtho is designed to analyze large-scale genomic data on a normal desktop computer in a reasonable time. In our tests, SwiftOrtho's homology search module was nearly 30 times faster than BLASTP. The orthology inference module of SwiftOrtho was nearly 500 times faster than OrthoMCL when applied to *Euk*. When applied to the large-scale dataset, *Bac*, SwiftOrtho was the only program that finished the orthology inference test on a workstation with 32GB RAM. The cluster module of SwiftOrtho using APC can handle data that are much larger than the available RAM. In our test, APC has comparable recall and accuracy, but requires considerably less memory than MCL. It should be noted that APC improved the F_1-measure score by increasing recall in most cases. With the help of these optimized modules, SwiftOrtho has successfully finished an orthology analysis of proteins from 1,760 bacterial genomes on a machine with only 4GB RAM, which makes SwiftOrho usable for large scale analyses for researchers who may not have access to expensive computational resources. SwiftOrtho is not only fast but also accurate, as shown in the results produced when running on orthobench and QfO[13, 33].

Potential implications

In summary, SwiftOrtho is a fast and accurate orthology prediction tool that can analyze a large number of sequences with minimal computational resource use. The installation and configuration of SwiftOrtho is simple and does not require the user to have any experience in database configuration. It is easy to use, as the only input required by SwiftOrtho is a FASTA format file of protein sequences with taxonomy information in the header line. SwiftOrtho can be integrated into various common pipelines where fast orthology classification is required such as pan-genome analysis, large-scale phylogenetic tree construction, and other multi-genome analyses. It is specifically suited for microbial community analyses, where large number of sequences and species are involved.

Methods

Algorithms

Here we outline the homology search, orthology inference, and clustering as implemented in SwiftOrtho.

Homology Search

SwiftOrtho employs a seed-and-extension algorithm to find homologous gene pairs [46, 47]. At the seed phase, SwiftOrtho finds candidate target sequences that share common k-mers with the query sequence. k-mer size is an important factor that affects search sensitivity and speed [38, 48]. SwiftOrtho therefore uses long (≥ 6) k-mers to accelerate search speed. At the same time, k-mer length is negatively correlated with sensitivity [38]. To compensate for the loss of sensitivity caused by increasing the k-mer size, SwiftOrtho uses two approaches: non-consecutive k-mers and reduced amino-acid alphabets. Non-consecutive k-mer seeds (known as spaced seeds), were introduced in PatternHunter [49, 19]. The main difference between consecutive seeds and spaced seeds is that the latter allow mismatches in alignment. For example, the spaced seed

101101 allows mismatches at positions 2 and 5. The total number of matched positions in a spaced seed is known as the weight, so the weight of this seed is 4. A consecutive seed can be considered as a special case of spaced seed in which its weight equal its length. Spaced seeds often provide a better sensitivity than consecutive seeds [50, 49]. Several tools such as PatternHunter, Usearch, LAST, and DIAMOND [49, 19, 39, 40, 41] have used spaced seed to increase sensitivity. PatternHunter and Usearch allow users to use custom spaced seed. The default spaced seed patterns of SwiftOrtho are 1110100010001011, 11010110111 –two spaced seeds with weight of 8– but the user may define their own spaced seeds. Seed patterns were optimized using SpEED [50] and manual inspection. The choice of the spaced seeds and default alphabet are elaborated upon in the Methods section and in the Supplementary Materials. At the extension phase, SwiftOrtho uses a variation of the Smith-Waterman algorithm [51], the k-banded Smith-Waterman or k-SWAT, which only allows for k gaps [52]. k-SWAT fills a band of cells along the main diagonal of the similarity score matrix (Figure 6B), and the complexity of k-swat is reduced to $O(k \cdot min(n, m))$, where k is the maximum allowed number of gaps.

Reduced alphabets are used to represent protein sequences using an alternative alphabet that combines several amino acids into a single representative letter, based on common physico-chemical traits [53, 54, 55]. Compared with the original alphabet of 20 amino acids, reduced alphabets usually improve sensitivity [56, 57]. At the same time, reduced alphabets also introduce less specific seeds than the original alphabet, reducing the search speed.

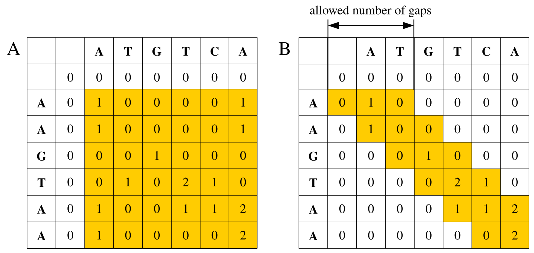

Figure 6. Comparing Standard Smith-Waterman with Banded Smith-Waterman. A. Similarity score matrix for Standard Smith-Waterman. Standard Smith-Waterman algorithm need to calculate all the entries. **B.** Similarity score matrix for Banded Smith-Waterman. Banded Smith-Waterman algorithm only need to calculate the entries on and near the diagonal.

Orthology Inference

The orthology inference step in Figure 1 shows the algorithm to infer orthologs and in-paralogs from homologs: gene A_1 in genome A and B_1 in genome B are considered to be orthologs according to the RBH rule. If the bit score between gene A_1 and A_2 in genome A is higher than that between A_1 and all its orthologs in other genomes, A_1 and A_2 are considered in-paralogs in genome A. If A_1 in genome A and B_1 in genome B are orthologs, in-paralogs of A_1 and B_1 are co-orthologs. Since orthology inference requires many queries it is better to store the data in a way that facilitates fast querying. First, SwiftOrtho sorts the data and stores it in the file system. Then, it uses binary search to query the sorted data, dramatically reducing memory usage when compared with a relational database management system or a hash table. With the help of this query system, SwiftOrtho can process data that are much larger than the computer memory.

The inferred relationships are treated as the edges of a graph. Each edge is assigned a weight for cluster analysis, where using appropriate edge-weighting metrics can improve the accuracy of cluster analysis. Gibbons [58], compared the performance of several BLAST-based edge-weighting metrics and found that the bit score has the best performance. Therefore, SwiftOrtho uses the normalized bit score as edge-weighting metric. The normalization step takes the same approach as OrthoMCL [20]. For orthologs or co-orthologs, the weight of (co-)ortholog (Figure 1) A_1 in genome A and B_1 in genome B is divided by the average edge-weight of all the (co-)orthologs between genome A and genome B. For in-paralogs, SwiftOrtho identifies a subset S of all in-paralogs in genome A, with each in-paralog A_x-A_y in subset S, A_x or A_y having at least one ortholog in another genome. The weight of each in-paralog in genome A is divided by the mean edge-weight of subset S in genome A [20].

Clustering Orthology Relationships into Orthologous Groups

SwiftOrtho provides two methods to cluster orthology relationships into orthologous groups. One is the Markov Cluster algorithm (MCL), an unsupervised clustering algorithm based on simulation of flow in graphs [21]. MCL is fast and robust on small networks and has been used by several graph-based tools [19, 59, 60, 61]. However, MCL may run out of memory when applied to a large-scale network. To reduce memory usage, we cluster each individual connected component instead of the whole network because there is no flow among components [21]. For large and dense networks a single connected component could still be too large to be loaded into memory.

For large networks, SwiftOrtho uses an Affinity Propagation Clustering algorithm (APC) [62]. The APC algorithm finds a set of centers in a network, where the centers are the actual data points and are called "exemplars". To find exemplars, APC needs to maintain two matrices: the responsibility matrix R, and the availability matrix A. The element $R_{i,k}$ in R reflects how well-suited node k is to serve as the exemplar for node i while the element $A_{i,k}$ in A reflects how appropriate node i to choose node k as its exemplar [62]. APC uses Equation 1 to update R, and Equation 2 to update A, where i, k, i', k' denote the node number, and $S_{i,k'}$ denotes the similarity between node i and node k'.

$$R_{i,k} = S_{i,k} - max_{k' \neq k}\{A_{i,k'} + S_{i,k'}\} \quad (1)$$

$$A_{i,k} = \begin{cases} min\{0, R_{k,k} + \sum_{i' \notin \{i,k\}} max\{0, R_{i',k}\}, & \text{if } i \neq k \\ \sum_{i' \neq k} max\{0, R_{i',k}\}, & \text{if } i = k \end{cases} \quad (2)$$

The node k that maximizes $A_{i,k} + R_{i,k}$ is the exemplar of node i, and each node i is assigned to its nearest exemplar. APC can update each element of matrix R and A one by one, so it is unnecessary to keep the whole matrix of R and A in memory. Generally, the time complexity of APC is $O(N^2 \cdot T)$ where N is number of nodes and T is number of iterations [62]. In this case, the time complexity is $O(E \cdot T)$, where E stands for edges which is number of orthology relationships and T is number of iterations. We implemented APC in Python, using Numba [63] to accelerate the numeric-intensive calculation parts.

Availability of source code and requirements (optional, if code is present)

The software and related information are listed below:

Project Name: SwiftOrtho

Project Home Page: https://github.com/Rinoahu/SwiftOrtho

Operating System(s): SwiftOrtho was tested on GNU/Linux distribution Ubuntu 16.04 64-bit, but we expect SwitOrtho to work on most *nix systems

Programming Language: Python

Other Requirements: Python 2.7, Python 3.7, PyPy2.7 v7.0 or higher

License: GPLv3

RRID: SCR_017122

Availability of supporting data and materials

The data sets supporting the results of this article are available in the GigaDB repository, http://dx.doi.org/10.5524/100633.

Declarations

List of abbreviations

APC: Affinity Propagation Clustering; **MCL:** Markov Clustering algorithm; **RBH:** Reciprocal Best Hit; **QFO:** Quest for Orthologs;

Ethical Approval

Not applicable

Consent for publication

Not Applicable

Competing Interests

The author(s) declare that they have no competing interests

Funding

This study has been funded, in part, by NSF award ABI 1458359. The funders had no role in the design of the study and collection, analysis, and interpretation of data and in writing the manuscript.

Author's Contributions

IF and XH conceived the study. XH wrote the software, performed the analysis. Both authors have written the manuscript.

Acknowledgements

The authors acknowledge fruitful discussions with all members of the Friedberg Lab.

Author information

IF is an Associate Professor at the Department of Veterinary Microbiology and Preventive Medicine at Iowa State University. He is also the chair of the Interdepartmental Bioinformatics and Computational Biology graduate program. XH currently is a postdoc at the Gianforte School of Computing, Montana State University.

References

1. Koonin EV. Orthologs, paralogs, and evolutionary genomics. Annu Rev Genet 2005;.
2. Fitch WM. Distinguishing Homologous from Analogous Proteins. Syst Zool 1970;19(2):99. https://academic.oup.com/sysbio/article-lookup/doi/10.2307/2412448.
3. Overbeek R, Fonstein M, D 'souza M, Pusch GD, Maltsev N. The use of gene clusters to infer functional coupling. Genetics 1999;96:2896–2901. http://www.pnas.org/content/96/6/2896.full.pdf.
4. Rivera MC, Jain R, Moore JE, Lake JA. Genomic evidence for two functionally distinct gene classes. Genetics 1998;95:6239–6244. https://www.ncbi.nlm.nih.gov/pmc/articles/PMC27643/pdf/pq006239.pdf.
5. Remm M, Storm CEVV, Sonnhammer ELLL. Automatic clustering of orthologs and in-paralogs from pairwise species comparisons. J Mol Biol 2001 dec;314(5):1041–1052. https://ac.els-cdn.com/S0022283600951970/1-s2.0-S0022283600951970-main.pd
6. O'Brien KP, Remm M, Sonnhammer ELL. Inparanoid: a comprehensive database of eukaryotic orthologs. Nucleic Acids Res 2005 jan;33(Database issue):D476–80. http://www.ncbi.nlm.nih.gov/pubmed/15608241http://www.pubmedcentral.nih.g
7. Gabaldón T, Koonin EV. . Nature Reviews Genetics 2013 apr;14(5):360–366. http://www.nature.com/doifinder/10.1038/nrg3456.
8. Goodman M, Czelusniak J, Moore GW, Romero-Herrera AE, Matsuda G. Fitting the Gene Lineage into its Species Lineage, a Parsimony Strategy Illustrated by Cladograms Constructed from Globin Sequences. Syst Biol 1979;28(2):132–163. http://sysbio.oxfordjournals.org/content/28/2/132{%}5Cnhttp://sysbio.oxfor
9. Kristensen DM, Wolf YI, Mushegian AR, Koonin EV. . Briefings in bioinformatics 2011 sep;12(5):379–91. http://www.ncbi.nlm.nih.gov/pubmed/21690100http://www.pubmedcentral.nih.g
10. Gabaldón T. Large-scale assignment of orthology: back to phylogenetics? Genome Biol 2008 oct;9(10):235. http://genomebiology.biomedcentral.com/articles/10.1186/gb-2008-9-10-235.
11. Hulsen T, Huynen MA, de Vlieg J, Groenen PMA. Benchmarking ortholog identification methods using functional genomics data. Genome Biol 2006;7(4):R31. http://www.ncbi.nlm.nih.gov/pubmed/16613613http://www.pubmedcentral.nih.g
12. Kuzniar A, van Ham RCHJ, Pongor S, Leunissen JAM, The quest for orthologs: finding the corresponding gene across genomes; 2008.
13. Trachana K, Larsson TA, Powell S, Chen WH, Doerks T, Muller J, et al. Orthology prediction methods: a quality assessment using curated protein families. Bioessays 2011 oct;33(10):769–80. https://www.ncbi.nlm.nih.gov/pmc/articles/PMC3193375/pdf/bies0033-0769.pd
14. Ward N, Moreno-Hagelsieb G. Quickly finding orthologs as reciprocal best hits with BLAT, LAST, and UBLAST: How much do we miss? PLoS One 2014;9(7).
15. Tatusov RL, Galperin MY, Natale DA, Koonin EV. The COG database: a tool for genome-scale analysis of protein functions and evolution. Nucleic Acids Res 2000 jan;28(1):33–36. http://www.ncbi.nlm.nih.gov/pubmed/10592175http://www.pubmedcentral.nih.g
16. Roth ACJ, Gonnet GH, Dessimoz C. Algorithm of OMA for large-scale orthology inference. BMC Bioinformatics 2008 dec;9(1):518. http://bmcbioinformatics.biomedcentral.com/articles/10.1186/1471-2105-9-5
17. Altenhoff AM, Glover NM, Train CM, Kaleb K, Warwick Vesztrocy A, Dylus D, et al. The OMA orthology database in 2018: Retrieving evolutionary relationships among all domains of life through richer web and programmatic interfaces. Nucleic Acids Res 2018;.
18. Alexeyenko A, Tamas I, Liu G, Sonnhammer ELL. Automatic clustering of orthologs and inparalogs shared by multiple proteomes. In: Bioinformatics; 2006. .
19. Li M, Ma B, Kisman D, Tromp J. PatternHunter II: highly sensitive and fast homology search. Genome Inform 2003 jan;14(03):164–75. http://citeseerx.ist.psu.edu/viewdoc/download?doi=10.1.1.1.2393{&}rep=rep
20. Fischer S, Brunk BP, Chen F, Gao X, Harb OS, Iodice JB, et al. Using OrthoMCL to assign proteins to OrthoMCL-DB groups or to cluster proteomes into new ortholog groups. Curr Protoc Bioinforma 2011;.
21. van Dongen S. Graph clustering by flow simulation. Graph Stimul by flow Clust 2000;PhD thesis:University of Utrecht.
22. Sonnhammer ELL, Koonin EV. Orthology, paralogy and proposed classification for paralog subtypes. Trends Genet 2002 dec;18(12):619–620. https://www.sciencedirect.com/science/article/pii/S0168952502027932?via{%
23. Cannon SB, Young ND. OrthoParaMap: Distinguishing orthologs from paralogs by integrating comparative genome data

and gene phylogenies. BMC Bioinformatics 2003;.

24. Cutts T, Down T, Dyer SC, Fitzgerald S, Fernandez-Banet J, Graf S, et al. Ensembl 2007. Nucl Acids Res 2007;.
25. Ruan J, Li H, Chen Z, Coghlan A, Coin LJM, Guo Y, et al. TreeFam: 2008 Update. Nucleic Acids Res 2008;.
26. Goodstadt L, Ponting CP. Phylogenetic reconstruction of orthology, paralogy, and conserved synteny for dog and human. PLoS Comput Biol 2006;.
27. Vilella AJ, Severin J, Ureta-Vidal A, Heng L, Durbin R, Birney E. EnsemblCompara GeneTrees: Complete, duplication-aware phylogenetic trees in vertebrates. Genome research 2009 feb;19(2):327–35. http://www.ncbi.nlm.nih.gov/pubmed/19029536http://www.pubmedcentral.nih.gov/articlerender.fcgi?artid=PMC2652215.
28. Chen F, Mackey AJ, Vermunt JK, Roos DS. Assessing performance of orthology detection strategies applied to eukaryotic genomes. PLoS One 2007 apr;2(4):e383. http://www.ncbi.nlm.nih.gov/pubmed/17440619http://www.pubmedcentral.nih.gov/articlerender.fcgi?artid=PMC1849888.
29. Altenhoff AM, Dessimoz C. Phylogenetic and functional assessment of orthologs inference projects and methods. PLoS Comput Biol 2009 jan;5(1):e1000262. http://dx.plos.org/10.1371/journal.pcbi.1000262.
30. Sonnhammer ELL, Östlund G. InParanoid 8: orthology analysis between 273 proteomes, mostly eukaryotic. Nucleic acids research 2015 jan;43(Database issue):D234–9. http://www.ncbi.nlm.nih.gov/pubmed/25429972http://www.pubmedcentral.nih.gov/articlerender.fcgi?artid=PMC4383983.
31. Cosentino S, Iwasaki W. SonicParanoid: Fast, accurate and easy orthology inference. Bioinformatics 2019;.
32. Lechner M, Findeiß S, Steiner L, Marz M, Stadler PF, Prohaska SJ. Proteinortho: Detection of (Co-)orthologs in large-scale analysis. BMC Bioinformatics 2011;.
33. Altenhoff AM, Boeckmann B, Capella-Gutierrez S, Dalquen DA, DeLuca T, Forslund K, et al. Standardized benchmarking in the quest for orthologs. Nat Methods 2016;.
34. Curwen V, Eyras E, Andrews TD, Clarke L, Mongin E, Searle SMJ, et al. The Ensembl automatic gene annotation system. Genome Res 2004;14(5):942–950.
35. Benson DA. GenBank. Nucleic Acids Res 2000;28(1):15–18. https://academic.oup.com/nar/article-lookup/doi/10.1093/nar/28.1.15.
36. Camacho C, Coulouris G, Avagyan V, Ma N, Papadopoulos J, Bealer K, et al. BLAST+: architecture and applications. BMC Bioinformatics 2009;.
37. Brohée S, van Helden J. Evaluation of clustering algorithms for protein-protein interaction networks. BMC Bioinformatics 2006;.
38. Kent WJ. BLAT — The BLAST -Like Alignment Tool. Genome Research 2002;12:656–664.
39. Edgar RC. Search and clustering orders of magnitude faster than BLAST. Bioinformatics 2010;26(19):2460–2461.
40. Kiełbasa SM, Wan R, Sato K, Horton P, Frith MC. Adaptive seeds tame genomic sequence comparison. Genome Res 2011;21(3):487–493.
41. Buchfink B, Xie C, Huson DH, Fast and sensitive protein alignment using DIAMOND; 2014.
42. Medlar A, Holm L. TOPAZ: Asymmetric suffix array neighbourhood search for massive protein databases. BMC Bioinformatics 2018;.
43. Rigo A, Pedroni S. PyPy ' s Approach to Virtual Machine Construction. Companion to 21st ACM SIGPLAN Symp 2006;p. 944–953.
44. Bratlie MS, Johansen J, Sherman BT, Huang DW, Lempicki RA, Drabløs F. Gene duplications in prokaryotes can be associated with environmental adaptation. BMC Genomics 2010;.
45. Katju V, Bergthorsson U, Copy-number changes in evolution: Rates, fitness effects and adaptive significance; 2013.
46. Pearson WR, Lipman DJ. Improved tools for biological sequence comparison. Proc Natl Acad Sci 1988;85(8):2444–2448. http://www.pnas.org/cgi/doi/10.1073/pnas.85.8.2444.
47. Altschul SF, Gish W, Miller W, Myers EW, Lipman DJ. Basic local alignment search tool. J Mol Biol 1990;215(3):403–410.
48. Shiryev SA, Papadopoulos JS, Schäffer AA, Agarwala R, Schaffer AA, Agarwala R. Improved BLAST searches using longer words for protein seeding. Bioinformatics 2007 nov;23(21):2949–2951. https://academic.oup.com/bioinformatics/article-lookup/doi/10.1093/bioinf
49. Ma B, Tromp J, Li M. PatternHunter: faster and more sensitive homology search. Bioinformatics 2002;18(3):440–445. https://academic.oup.com/bioinformatics/article-lookup/doi/10.1093/bioinf
50. Ilie L, Ilie S, Khoshraftar S, Bigvand AM. Seeds for effective oligonucleotide design. BMC Genomics 2011 jan;12(1):280. http://www.pubmedcentral.nih.gov/articlerender.fcgi?artid=3128067{&}tool=p
51. Smith TF, Waterman MS. Identification of common molecular subsequences. J Mol Biol 1981 mar;147(1):195–197. http://www.ncbi.nlm.nih.gov/pubmed/7265238http://linkinghub.elsevier.com/
52. Chao KM, Pearson WR, Miller W. Aligning two sequences within a specified diagonal band. Bioinformatics 1992 oct;8(5):481–487. http://www.ncbi.nlm.nih.gov/pubmed/1468007.
53. Landès C, Risler JL. Fast databank searching with a reduced amino-acid alphabet. Computer applications in the biosciences : CABIOS 1994 Jul;10(4):453–454. http://view.ncbi.nlm.nih.gov/pubmed/7804879.
54. Murphy LR, Wallqvist A, Levy RM. Simplified amino acid alphabets for protein fold recognition and implications for folding. Protein Eng Des Sel 2000 mar;13(3):149–152. https://academic.oup.com/peds/article-lookup/doi/10.1093/protein/13.3.149
55. Peterson EL, Kondev J, Theriot JA, Phillips R. Reduced amino acid alphabets exhibit an improved sensitivity and selectivity in fold assignment. Bioinformatics (Oxford, England) 2009 Jun;25(11):1356–1362. http://dx.doi.org/10.1093/bioinformatics/btp164.
56. Edgar RC. Local homology recognition and distance measures in linear time using compressed amino acid alphabets. Nucleic acids research 2004;32(1):380–5. http://www.ncbi.nlm.nih.gov/pubmed/14729922http://www.pubmedcentral.nih.g
57. Ye Y, Choi JH, Tang H. RAPSearch: a fast protein similarity search tool for short reads. BMC Bioinformatics 2011 may;12(1):159. http://bmcbioinformatics.biomedcentral.com/articles/10.1186/1471-2105-12-
58. Gibbons TR, Mount SM, Cooper ED, Delwiche CF. Evaluation of BLAST-based edge-weighting metrics used for homology inference with the Markov Clustering algorithm. BMC Bioinformatics 2015;16(1). https://www.ncbi.nlm.nih.gov/pmc/articles/PMC4496851/pdf/12859{_}2015{_}A
59. Enright AJ, Van Dongen S, Ouzounis CA. An efficient algorithm for large-scale detection of protein families. Nucleic Acids Res 2002 apr;30(7):1575–1584. http://www.pubmedcentral.nih.gov/articlerender.fcgi?artid=101833{&}tool=p
60. Emms DM, Kelly S. OrthoFinder: solving fundamental biases in whole genome comparisons dramatically improves orthogroup inference accuracy. Genome Biology 2015 dec;16(1):157. http://genomebiology.com/2015/16/1/157.
61. Davis JJ, Gerdes S, Olsen GJ, Olson R, Pusch GD, Shukla M, et al. PATtyFams: Protein families for the microbial genomes in the PATRIC database. Front Microbiol 2016;7(FEB):118. http://www.ncbi.nlm.nih.gov/pubmed/26903996http://www.pubmedcentral.nih.g
62. Frey BJ, Dueck D. Clustering by passing messages between data points. Science 2007 feb;315(5814):972–6. http://www.ncbi.nlm.nih.gov/pubmed/17218491http://science.sciencemag.org/
63. Lam SK, Pitrou A, Seibert S. Numba: A LLVM-based python JIT compiler. Proc Second Work LLVM Compil Infrastruct HPC - LLVM '15 2015;p. 1–6.

Reference PDF
Click here to access/download;Manuscript;main.pdf
Click here to view linked References

OXFORD
(GIGA)n SCIENCE

GigaScience, 2017, 1–10

doi: xx.xxxx/xxxx
Manuscript in Preparation
Paper

PAPER

SwiftOrtho: a Fast, Memory-Efficient, Multiple Genome Orthology Classifier

Xiao Hu[1,2*] and Iddo Friedberg[1*]

[1]Department of Veterinary Microbiology and Preventive Medicine, College of Veterinary Medicine, Iowa State University, Ames Iowa USA and [2]Present address: Gianforte School of Computing, Montana State University, Bozeman, Montana, USA

*xiao.hu1@montana.edu; idoerg@iastate.edu

Abstract

Background: Gene homology type classification is required for many types of genome analyses, including comparative genomics, phylogenetics, and protein function annotation. Consequently, a large variety of tools have been developed to perform homology classification across genomes of different species. However, when applied to large genomic datasets, these tools require high memory and CPU usage, typically available only in computational clusters.
Findings: Here we present a new graph-based orthology analysis tool, SwiftOrtho, which is optimized for speed and memory usage when applied to large-scale data. SwiftOrtho uses long k-mers to speed up homology search, while using a reduced amino acid alphabet and spaced seeds to compensate for the loss of sensitivity due to long k-mers. In addition, it uses an Affinity Propagation algorithm to reduce the memory usage when clustering large-scale orthology relationships into orthologous groups. In our tests, SwiftOrtho is the only tool that completed orthology analysis of proteins from 1,760 bacterial genomes on a computer with only 4GB RAM. Using various standard orthology datasets, we also show that SwiftOrtho has a high accuracy.
Conclusion: SwiftOrtho enables the accurate comparative genomic analyses of thousands of genomes using low memory computers.
Availability: SwiftOrtho is available at: https://github.com/Rinoahu/SwiftOrtho

Key words: Orthology Analysis; Homology Search; Orthology Inference; Clustering; Orthologs; Paralogs

Background

Gene homology type classification consists of identifying paralogs and orthologs across species. Orthologs are genes that evolved from a common ancestral gene following speciation, while paralogs are genes that are homologous due to duplication. Paralogs can be further classified into in-paralogs, which evolved via gene duplication before the speciation event, and out-paralogs, which evolved via gene duplication after the speciation event [1]. Classifying orthologs and paralogs across species is an important problem, as the evolutionary history of genes has implications for our understanding of gene function and evolution.

While the proper inference of homology type involves tracing gene history using phylogenetic trees [2], several proxy methods have been developed over the years. The most common method to infer orthologs by proxy is Reciprocal Best Hits or RBH [3, 4]. Briefly, RBH states the following: when two proteins that are encoded by two genes, each in a different genome, find each other as the best scoring match among all homologs, they are considered to be orthologs [3, 4].

InParanoid extends the RBH orthology relationship to include both orthologs and in-paralogs. Specifically, InParanoid uses RBH to identify orthologs between two species. The genes in the two species are classified as in-paralogs if they are more similar to the corresponding ortholog than to any gene in the other species [5, 6, 7]. The concept of orthologous pairs between two species can be extended to an *ortholog group*, which is a set of genes that are hypothesized to have descended from a common ancestor [7]. Several methods have been developed to identify ortholog groups across multiple species typically classified as either tree-based or graph-based methods. Tree-based methods construct a gene tree from an alignment of homologous sequences in different species and infer orthology rela-

Compiled on: August 30, 2019.
Draft manuscript prepared by the author.

tionships by reconciling the gene tree with its corresponding species tree [2, 8, 9], and can infer a correct orthology relationship if the correct gene tree and species tree are provided [10]. The chief limiting factor of tree-based methods is the accuracy of the given gene tree and species tree. Erroneous trees lead to incorrect ortholog and in-paralog assignments [11, 10, 9]. Tree-based methods are also computationally expensive which limits the ability to apply them to large number of species [12, 10, 13, 14]. Graph-based methods infer orthologs and in-paralogs from homologs and then use different strategies to cluster them into orthologous groups [12, 9, 13] (Figure 1). The Clusters of Orthologous Groups (COG) database detects triangles of RBHs in three different species and merges the triangles with a common side [15]. Orthologous Matrix (OMA) clusters RBHs in orthologous groups by finding maximum weight cliques from the similarity graph [16, 17]. MultiParanoid is an extension of InParanoid, which uses InParanoid to detect triangle orthologs and in-paralogs in three different species as seeds and then merges the seeds into larger groups [18]. OrthoMCL also uses InParanoid to detect orthologs, co-orthologs, and in-paralogs between two species [19, 20] and then uses Markov Clustering (MCL) [21] to cluster these relationships into orthologous groups, where the co-orthologs are two or more genes in one species that are orthologous to one or more genes in another species due to a gene duplication event [22, 1].

Finally, there are hybrid methods that combine both graph-based and tree-based methods [12, 23, 24, 25, 26]. Typically, hybrid methods first perform all-*vs*-all sequence alignment, then construct gene families by sequence similarity or conserved gene neighborhood. EnsEMBL first uses RBH to find the gene families, then constructs a phylogenetic gene tree for each gene family [24]. Finally, each gene tree is reconciled with the species tree to infer paralogs and orthologs.

In theory, graph-based methods are less accurate than tree-based methods, as the former identify orthologs and in-paralogs using proxy methods rather than directly inferring homology type from gene and species evolutionary history. However, graph-based methods have been found to be comparably accurate to tree-based methods [11, 10, 27]. Moreover, a comparison of several methods found that tree-based methods had even a worse performance than graph-based methods on large datasets [11].

One study compared several common methods, including simple RBH, graph-based, tree-based, and hybrid methods, and found that the tree-based methods of InParanoid and OrthoMCL exhibit the best balance of sensitivity and specificity [28]. Several studies have also shown that graph-based methods find a better trade-off between specificity and sensitivity than tree-based methods [11, 28, 29]. For these reasons, graph-based methods are generally preferred for analyzing large-scale data sets. OrthoMCL and InParanoid have been applied to analyze hundreds of genomes; at the same time, they require considerable computational resources that may not be readily available [20, 30]. More recently, several graph-based tools, such as SonicParanoid, OMA, and ProteinOrtho [31, 17, 32] have been developed to speed up orthology analysis on large-scale data sets. There tools also tend to require high performance computers with large memory to analyze large-scale data.

Here we present SwiftOrtho, a fast method for orthology classification that makes minimal use of computational resources, especially memory. SwiftOrtho uses a seed-and-extension method to speed up homology search, a binary search method and RBH rule to infer orthologs and in-paralogs, and the Affinity Propagation algorithm to reduce memory usage in cluster analysis. We compare SwiftOrtho with several existing graph-based tools using the gold standard dataset Orthobench [13], and the Quest for Orthologs service [33]. Using both benchmarks, we show that SwiftOrtho provides a high accuracy with lower CPU and memory usage than other graph-based methods. SwiftOrtho is the only tool that completed an orthology analysis of 1,760 bacterial genomes on very a low-memory computer. With the growing number of genomes, especially microbial genomes, we see SwiftOrtho to be a tool of choice for a fast and accurate ortholog classification, while requiring low computational resources, as are found in conventional desktop or laptop computers.

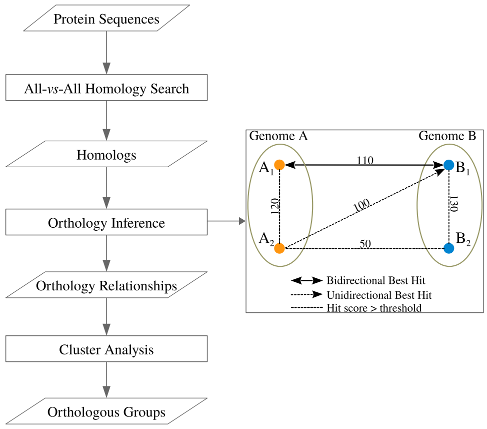

Figure 1. The flow cart of SwiftOrtho. SwiftOrtho is a graph-based method which consist of three major steps: **All-*vs*-All Homology Search**: A seed-and-extension method is used to perform homology search; **Orthology Inference:** Nodes are gene names, edges are similarity score of pairwise genes. 1. A_1-B_1 are putative orthologs identified by RBH. 2. A_1-A_2 and B_1-B_2 are putative in-paralogs as the bit scores of these pairs greater than A_1-B_1; 3. A_2-B_1 and A_2-B_2 are putative co-orthologs as these pairs are not orthologs but A_1-B_1 are orthologs and A_1-A_2, B_1-B_2 are in-paralogs; **Cluster Analysis:** Markov clustering or Affinity Propagation Algorithm is used to cluster orthology relationships.

Application of SwiftOrtho

Data Sets

We applied SwiftOrtho to three data sets to evaluate its predictive quality and performance:

i. The *Euk* set was used to evaluate the quality of predicted orthologous groups. This set contains 420,415 protein sequences from 12 eukaryotic species, including *Caenorhabditis elegans, Drosophila melanogaster, Ciona intestinalis, Danio rerio, Tetraodon nigroviridis, Gallus gallus, Monodelphis domestica, Mus musculus, Rattus norvegicus, Canis familiaris, Pan troglodytes* and *Homo sapiens*. The protein sequences for these genes were downloaded from EMBL v65 [34].
ii. The *QfO 2011* set was used to evaluate the quality of predicted orthology relationships. This set was the reference proteome dataset (2011) of The Quest for Orthologs[33], which contains 754,149 protein sequences of 66 species.
iii. The large *Bac* set was used to evaluate performance, including CPU time, real time and RAM usage. This set includes 5,950,817 protein sequences from 1,760 bacterial species. The protein sequences were downloaded from GenBank [35]. For a full list, see the additional file 1.

We also compared SwiftOrtho with several existing orthology analysis tools for predictive quality and performance. The methods compared were: OrthoMCL(v2.0), FastOrtho, OrthAgogue, and OrthoFinder.

Orthology Analysis Pipeline

The pipeline for all the tools follows the standard steps of graph-based orthology prediction, (1) all-*vs*-all homology search, (2) orthology inference, and (3) cluster analysis.

Homology Search
SwiftOrtho used its built-in module to perform all-*vs*-all homology search. For all the three sets, the E-value was set 10^{-5}. The amino acid alphabet was set to the regular 20 amino acids for the three sets. The spaced seed parameter was set to `1011111,11111` for the `Euk`, `11111111` for the *QfO 2011*, and `111111` for *Bac*.

OrthoMCL, FastOrtho, OrthAgogue, and OrthoFinder use BLASTP (v2.2.27+) [36] to perform all-*vs*-all homology search. The first three tools require the user to do this manually. To compare the methods, the -e (e-value), -v (number of database sequences to show one-line descriptions), and -b (number of database sequence to show alignments) parameters of BLASTP were set to 10^{-5}, 1,000,000, and, 1,000,000 for OrthoMCL, FastOrtho, and OrthAgogue. The OrthoFinder calls BLASTP, and the E-value of BLASTP have been set to 10^{-3}.

Orthology Inference
SwiftOrtho, OrthoMCL, FastOrtho, OrthAgogue, and OrthoFinder were applied to perform orthology inference on the homologs. The first four tools are able to identify (co-)orthologs and in-paralogs, and the coverage (fraction of aligned regions) was set to 50%, while other parameters were set to their default values, see Supplementary Materials for full details.

FastOrtho does not report (co-)orthologs and in-paralogs directly. However, the relevant information is stored in an intermediate file, from which we have extracted that information. Orthofinder does not report orthology relationships.

Cluster Analysis
All the tools in this study use MCL [21] for clustering. To control the granularity of the clustering, MCL performs an inflation operation set by the *-I* option [21, 37]. In this study, *-I* was set to 1.5. To take advantage of multiprocessor capabilities, we set the thread number of MCL to 12. SwiftOrtho has an alternative clustering algorithm APC, which we have also applied to *Euk* and*Bac*.

Evaluation of Prediction Quality

Evaluation of Predicted Orthologous Groups
The OrthoBench set was used to evaluate the quality of predicted orthologous groups in *Bac*. This set contains 70 manually curated orthologous groups of the 12 species from *Bac* and has been used as a high quality gold standard benchmark set for orthologous group prediction [13]. We used OrthoBench v2 (Supplementary Table S1). Each manually curated group of OrthoBench v2 set finds the best match in the predicted orthologous groups, where the best match means that the number of genes shared between manually curated and predicted orthologs is maximized, and the method to calculate precision and recall is shown in Supplementary Figure S1.

Evaluation of Predicted Orthology Relationships
The *Quest of Orthologs* web-based service (QfO) was employed to evaluate the quality of the orthology relationships predicted from the *QfO 2011* set[33]. The QfO service evaluates the predictive quality by performing four phylogeny-based tests of *Species Tree Discordance Benchmark*, *Generalized Species Tree Discordance Benchmark*, *Agreement with Reference Gene Phylogenies: SwissTree*, and *Agreement with Reference Gene Phylogenies: TreeFam-A*, and two function-based tests of *Gene Ontology conservation test* and *Enzyme Classification conservation test* [33].

We also applied two more orthology prediction tools, SonicParanoid[31] and InParanoid (v4.1)[5], on the *QfO 2011* set and used their results as control because InParanoid has best performance among the results from QfO service website and SonicParanoid is a fast implementation of InParanoid. The pairwise orthology relationships were extracted from the predicted orthologous groups of all the tools, including SonicParanoid and InParanoid, and then submitted to the QfO web-service for further evaluation.

Hardware

Unless specified otherwise, all tests were run on the Condo cluster of Iowa State University with Intel Xeon E5-2640 v3 at 2.60GHz, 128GB RAM, 28TB free disk. The Linux command `time -v` was used to track CPU and peak memory usage.

Findings

We compared the orthology analysis performance of SwiftOrtho, OrthoMCL, FastOrtho, OrthAgogue, and OrthFinder using *Euk*, *QfO 2011*, and *Bac*. The orthology analysis consists of homology search, orthology inference, and cluster analysis.

Orthology Analysis on *Euk*

The results of orthology analysis on *Euk* are summarized in Table 1, and are elaborated upon below.

		SwiftOrtho		OrthoMCL	FastOrtho	OrthAgogue	OrthoFinder
**Homology Search**	Method	SO built-in		BLASTP			
	Hits	162,695,330		947,203,546			654,792,861
	Uniq Hits	162,695,330		297,107,872			266,104,611
**Orthology Inference**	(Co-)orthologs	1,422,920		8,279,424	3,297,613	1,265,553	N/A
	In-paralogs	631,033		2,517,166	2,546,296	759,989	N/A
**Clustering**	Algorithm	MCL	APC	MCL			
	Orthologous Groups	44,551	38,748	36,901	40,943	51,297	19,904

Table 1. Comparative orthology analysis on the *Euk* set. N/A: not available, SO: SwiftOrtho, MCL: Markov Clustering, APC: Affinity Propagation Cluster.

Homology Search
The homology search results show that BLASTP detected the largest number of homologs, 947,203,546. SwiftOrtho found 57.5% of the homologs detected by BLASTP but was 38.7 times faster than BLASTP. SwiftOrtho used longer k-mers, which reduced both specific and non-specific seed extension. The longer k-mers cause seed-and-extension methods to ignore sequences with low similarity. According to the RBH rule, orthologs should have higher similarity than non-orthologs, so, the decrease in homolgs of SwiftOrtho does not significantly affect the next orthology inference.

We compared RBHs inferred from homologs detected by BLASTP and SwiftOrtho, and the numbers of RBHs for BLASTP and SwiftOrtho are 899,473 and 957,387, respectively. Identical RBHs are 767,884 (85.37% of BLASTP). These results show that although SwiftOrtho found fewer homologs than BLASTP, it does not significantly reduce the number of RBHs. The following results in Figure 3 also show that there is no significant difference between SwiftOrtho and BLASTP in predicting orthologous groups. Homology searches against a large number of protein sequences are a major bottleneck in bioinformatics pipelines. For that reason, many tools have been developed to speed up this process including, among others, BLAT, Usearch, LAST, DIAMOND, and Topaz [38, 39, 40, 41, 42]. All these tools use longer k-mers than BLASTP to speed up performance. We also compared SwiftOrtho with them in speed and sensitivity, (Supplementary Table S9). Because BLASTP is widely considered the gold standard for comparing protein sequences, we use its results as the benchmark to evaluate the sensitivity of other homology search tools. We found Usearch and LAST to be the fastest, however, they only found 0.88% and 2.97% hits of BLASTP, respectively. Topaz and BLAT used the most CPU time, but found only 33.48% and 28.34% of the BLASTP hits, respectively. SwiftOrtho and DIAMOND (more sensitive mode) have

the highest sensitivity and found 52.72% and 58.30% of the BLASTP hits in a moderate amount of time, respectively. These results show that SwiftOrtho delivers a good trade-off between speed and sensitivity.

Orthology Inference

OrthoMCL and FastOrtho found more orthology relationships than SwiftOrtho and OrthAgogue. This is because OrthoMCL and FastOrtho use the negative log ratio of the e-value as the edge-weighting metric. The BLASTP program rounds E-value $< 10^{-180}$ to 0. Consequently, for homolgs with an e-value $< 10^{-180}$, OrthoMCL and FastOrtho treat them as the RBHs, overestimating the number of orthologs. An example showing the OrthoMCL and FastOrtho overestimation can be found in Table S4.

Use of Computational Resources

OrthoMCL v2.0 used the most CPU time and real time because of the required I/O operations. The RAM usage of OrthoMCL was 3.45GB, while the generated intermediate file occupied >19 TB of disk space. OrthAgogue was the most efficient in real time, because of its ability to exploit a multi-core processor. However, the RAM usage of OrthAgogue was more than 100GB which exceeds that of common workstations and many servers. The orthology inference module of FastOrtho was the most memory-efficient among all the tools and was also fast. SwiftOrtho was the most CPU time efficient, although its real time was twice as that of OrthAgogue. Because the orthology inference module of SwiftOrtho was written in pure Python, we retested it by using the PyPy interpreter, an alternate implementation of Python [43]. When running with PyPy the real run time of SwiftOrtho was close to that of OrthAgogue (Table S5)

Cluster Analysis

OrthoFinder identified the smallest number of orthologous groups. Other tools identified many more orthologous groups than OrthoFinder, ranging from 36,901 to 51,297. The APC algorithm found fewer clusters than the MCL algorithm.

Evaluation of Predicted Orthologous Groups

The quality of predicted orthologous groups is shown in Figure 2. OrthoFinder has the best recall, while SwiftOrtho and OrthAgogue have top precision values but lower recall values than other tools. Since SwiftOrtho and OrthAgogue use a more stringent standard to perform orthology inference, this strategy often increases precision but decreases recall [11, 28, 29].

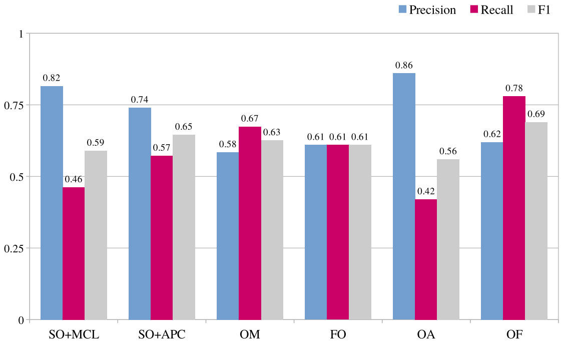

Figure 2. Evaluation of predicted orthologous groups. Evaluation of different tools on OrthoBench database. **SO+MCL**: SwiftOrtho with MCL; **SO+APC**: SwiftOrtho with Affinity Propagation Clustering; **OM**: OrthoMCL v2; **FO**: FastOrtho; **OA**: OrthAgogue; **OF**: OrthoFinder.

Because SwiftOrtho uses its built-in homology search module and its recall is lower than BLASTP's, it may reduce the recall of orthologous groups. To address this problem, we made two replacements. We replaced SwiftOrtho's homology module with BLASTP for SwiftOrtho and replaced BLASTP with SwiftOrtho's homology module for OrthoMCL, FastOrtho, OrthAgogue, and OrthoFinder. We then reran the orthology analysis on *Euk*. The results show that for most tools, replacing BLASTP with SwiftOrtho's built-in homology search module does not significantly reduce the recall (Figure 3). The difference in recall between using SwiftOrtho's homology search and using BLASTP is less than 4% except for OrthoMCL and FastOrtho. The recall for OrthoMCL and FastOrtho decreased by 8% and 7%, respectively. The most likely reason is that the E-value of SwiftOrtho's homology search module is more precise than that of BLASTP, which reduces the false RBHs as mentioned above. These results show that SwiftOrtho's homology search module is a reliable and fast alternative to BLASTP.

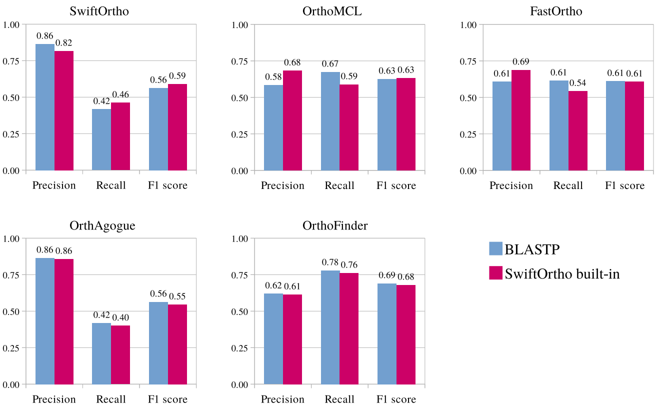

Figure 3. Comparing BLASTP and SwiftOrtho's homology search module on the quality of orthologous groups prediction. BLASTP and SwiftOrtho's search module perform an all-*vs*-all search on the *Euk* set, respectively. Then, all the orthology prediction tools were employed for orthology inference. Finally, the predicted orthology relationships were clustered into orthologous groups by MCL algorithm.

To test the differences exhibited by the clustering component of SwiftOrtho, we ran SwiftOrtho with MCL and APC on the same data. The results (Figure 4) show that performance of APC is close to that of MCL. APC improves the recall of most tools (Figure 4). These results show that APC has the similar performance as the MCL algorithm and is a reliable alternative to MCL.

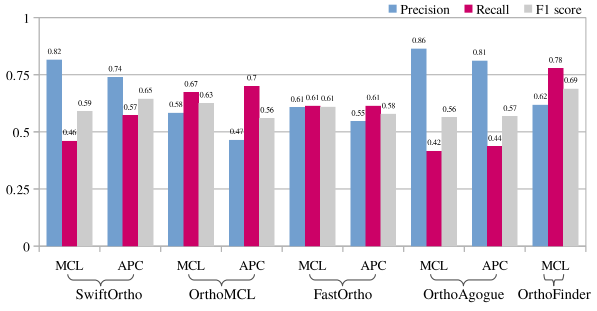

Figure 4. Markov Clustering versus Affinity Propagation Clustering. Both algorithms were applied to cluster the orthology relationships of the *Euk* set inferred by different orthology prediction tools, into orthologous groups. As OrthoFinder does not report orthology relationships, the Affinity Propagation can not be applied to its results. **MCL:** Markov Clustering algorithm; **APC:** Affinity Propagation Clustering.

Orthology Analysis on *QfO 2011*

The results of the orthology analysis on *QfO 2011* are shown in Table 2 and elaborated below.

		SwiftOrtho	OrthoMCL	FastOrtho	OrthAgogue	OrthoFinder
Homology Search	Method	SO built-in	BLASTP			
	Hits	183,883,417	642,372,369			935,579,809
	Uniq Hits	183,883,417	317,333,885			462,876,579
Orthology Inference	(Co-)orthologs	2,209,243	3,743,779	2,588,851	2,716,128	N/A
	In-paralogs	6,929,058	11,427,118	13,649,582	13,694,208	N/A
Clustering	Algorithm	MCL				
	Orthologous Groups	60,418	50,970	55,530	50,203	166,217

Table 2. Comparative orthology analysis on the Quest for Orthologs reference proteome 2011 dataset. SO: SwiftOrtho; MCL: Markov Clustering; APC: Affinity Propagation Cluster; N/A: not available.

Homology Search

SwiftOrtho found 183,883,417 unique hits while BLASTP found 462,876,579 unique hits. However, SwiftOrtho is about 163 times faster than BLASTP.

Orthology Inference

OrthoMCL found many more orthologs and co-orthologs than the other tools. SwiftOrtho found fewer in-paralogs than other available tools. The CPU time of SwiftOrtho is the least of all tools. When using the PyPy interpreter, the real time of SwiftOrtho is also close to that of the fastest one, OrthAgogue (Supplementary Table S6).

Cluster Analysis

Overall, the clustering numbers of SwiftOrtho, OrthoMCL, FastOrtho, and OrthAgogue are similar. However, the number of clusters found by OrthoFinder is three times that of other tools, and the next evaluation also shows that OrthoFinder performed poorly on *QfO 2011*.

Evaluation of Predicted Ortholog Relationships

The evaluation shows that the performance of SwiftOrtho is close to that of InParanoid (Figure 5). In some tests (Figure 5, D-E), SwiftOrtho outperformed InParanoid. SwiftOrtho had the best performance in the Generalized Species Tree Discordance Benchmark and Agreement with Reference Gene Phylogenies: TreeFam-A tests. In the Species Tree Discordance Benchmark, SwiftOrtho had the minimum Robinson-Foulds distance. In the Enzyme Classification (EC) conservation test, SwiftOrtho had the maximum Schlicker similarity. These two metrics reflect the accuracy of the algorithm, and the results show that SwiftOrtho has an overall higher accuracy than the other tools. At the same time, the recall of SwiftOrtho was lower in some of the QfO tests, the main reason is that SwiftOrtho uses an stringent metric system to identify orthology relationships.

Orthology Analysis On *Bac*

The results of orthology analysis on *Bac* are summarized in Table 3.

		SwiftOrtho		OrthoMCL	FastOrtho	OrthAgogue	OrthoFinder
Homology Search	Method	SO built-in					N/A
	Hits	8,478,732,753					N/A
	Uniq Hits	8,478,732,753					N/A
Orthology Inference	(Co-)orthologs	876,766,940		N/A	950,683,849	N/A	N/A
	In-paralogs	622,292		N/A	663,052	N/A	N/A
Clustering	Algorithm	MCL	APC	MCL			
	Orthologous Groups	240,162	167,355	N/A	242,816	N/A	N/A

Table 3. Comparative orthology analysis on the *Bac* set. SO: SwiftOrtho; MCL: Markov Clustering; APC: Affinity Propagation Cluster;
N/A: not available.

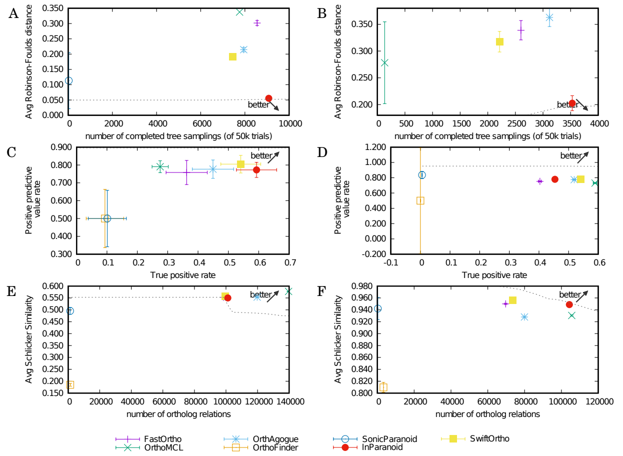

Figure 5. The Benchmarking in Quest for Orthologs. A: Species Tree Discordance Benchmark. InParanoid has minimum average Robinson-Foulds distance. SwiftOrtho's average RF distance is close to that of InParanoid. The prediction inferred by OrthoFinder is not available for this test; **B:** Generalized Species Tree Discordance Benchmark. InParanoid has minimum average Robinson-Foulds distance. The prediction inferred by OrthoFinder is not available for this test; **C:** Agreement with the Reference Gene Phylogenies of SwissTree. SwiftOrtho has the highest positive prediction value rate (recall). InParanoid has the highest true positive rate (precision); **D:** Agreement with Reference Gene Phylogenies of TreeFam-A. SonicParanoid has the highest positive prediction value rate (recall), however, its true positive rate (precision) is close to zero. SwiftOrtho has the second highest recall and precision; **E:** Gene Ontology conservation test. OrthoMCL has the highest average Schlicker similarity; **F:** Enzyme Classification conservation test. SwiftOrtho has the highest average Schlicker similarity. OrthoMCL detected the most orthology relationships and has the highest recall.

Homology Search

SwiftOrtho detected 8,966,131,536 homologs in the *Bac* set within 1,247 CPU hours.

Because it takes long time to perform all-*vs*-all BLASTP search on the full *Bac*, we randomly selected 1,000 protein sequences from *Bac* and used them to search against the full *Bac* set. It took BLASTP 5.1 CPU hours to find the homologs of these 1,000 protein sequences. We infer that the estimated CPU time of BLASTP on the full *Bac* set should be around 30,000 CPU hours. SwiftOrtho was almost 25 times faster than BLASTP on *Bac*.

Orthology Inference

SwiftOrtho, OrthoMCL, FastOrtho, and OrthAgogue were used to infer (co-)orthologs and in-paralogs from the homologs detected by the homology search module of SwiftOrtho in the *Bac* set. We did not test Orthofinder, because Orthofinder does not accept a single file of homologs as input. For the 1,760 proteomes in *Bac*, OrthoFinder needs to perform 3,097,600 pairwise species-by-species comparisons, which will generate the same number of files. Then, OrthoFinder performs the orthology inference on these 3,097,600 files. Even at one minute per file, it will take an estimated 6 CPU years to process all the files.

Due to memory limitations, only SwiftOrtho and FastOrtho finished the orthology inference on *Bac*. The results are shown in Table 3. The numbers of (co-)orthologs and in-paralogs inferred by SwiftOrtho and FastOrtho are similar. The number of common orthology relationships between SwiftOrtho and FastOrtho was 861,619,519 (98.2% of SwiftOrtho and 90.57% of FastOrtho). Compared with *Euk*, SwiftOrtho and FastOrtho have a similar predictive quality on *Bac*. There are three possible explainations for these results. The first is that *Euk* contains many protein isoforms which cause FastOrtho to overestimate the number of orthologs and in-paralogs. The second is that the gene duplication rate in Bacteria is lower than that in Eukaryotes [44, 45]. For *Bac*, each gene in one species has only small number of homologs in other species, which

makes FastOrtho unlikely to overestimate the number of RBHs. The third is that SwiftOrtho uses double-precision floating-point to store the E-value, which increases the precision of E-value from 10^{-180} to 10^{-308}. This improvement also reduces the possibility that FastOrtho may report false RBHs.

Computational resource use: Of the programs tested, only SwiftOrtho and FastOrtho finished the orthology inference step. FastOrtho and OrthAgogue did not finish the tests due to insufficient RAM; OrthoMCL aborted after running out of disk space, as it needed more than 18TB. The peak RAM usage of SwiftOrtho and FastOrtho were 90.6GB and 99.5GB, respectively. When we used the PyPy interpreter, the Peak RAM usage of SwiftOrtho was reduced to 72.1GB. FastOrtho was about 1.52 times faster than SwiftOrtho which ran the tests in the CPython interpreter. When using the PyPy interpreter, SwiftOrtho ran 1.58 times faster than FastOrtho. The memory usage and CPU time are shown in Table S7.

Cluster Analysis

The clustering numbers of SwiftOrtho and FastOrtho are similar. We compared the APC algorithm and the MCL algorithm, and APC found fewer clusters than MCL. The APC used much less memory and less CPU time than MCL. However, due to the lack of support for multi-threading and a large number of I/O operations, the real run time of APC is longer than that of MCL.

Tests on a Low-memory System

Because SwiftOrtho is designed to process large-scale data on low-memory computers, we used it to analyze *Bac* on a range of computers with different specifications.

The results show that the memory usage of SwiftOrtho is flexible and adapts to the size of the computer's memory. In the tests, SwiftOrtho finished an orthology analysis of *Bac* set on a computer with only 4GB RAM in a reasonable time (Table S8).

Comparison with other Orthology Analysis Pipelines

SonicParanoid, OMA, and ProteinOrth are also graph-based methods and have been optimized for large-scale data sets [31, 17, 32]. We compared SwiftOrtho with these tools in both speed and memory usage. The results are shown in Table S10. OMA appears to be the slowest as it it uses the Smith-Waterman algorithm to perform all-*vs*-all alignment. In our tests, OMA took 0.84 CPU hours to align two species (4,064 and 4,140 genes) of the *Bac* set. For the *Bac* set, OMA needs to perform 3,097,600 species-by-species alignments and the total time will be over two million CPU hours. SonicParanoid worked well on *Euk* and *QfO 2011* sets. Compared with SwiftOrtho, SonicParanoid ran faster and required less RAM on small data sets. However, it exited abnormally when applied to large *Bac* set. Proteinortho also worked well on the *Euk* and *QfO 2011* sets. When applied to the *Bac* set, Proteinortho needed to perform 1,547,920 species-by-species proteome alignments. It took Proteinortho 186.5 CPU hours, using DIAMOND, to complete 23,331 (1.5%) alignments; we therefore estimate that Proteinortho will take about 12,355 CPU hours to finish a full homology search. Since LAST is much faster than DIAMOND, we reran Proteinortho on the *Bac* set, using LAST for homology search. The CPU time for LAST on the *Bac* set was 2,368 hours. Although the previous results (Supplementary Table S9) show that LAST is about 20 times faster than SwiftOrtho, LAST required much more CPU time than SwiftOrtho in the all-*vs*-all homology search step. We think it is because the species-by-species alignment approach requires more than 1.5 million I/O operations, which significantly reduces the speed. The CPU utilization of orthology inference and clustering of Proteinortho was very low (less than 10%) when applied to the *Bac* set, which led to an exceptionally long real time run (more than 150 hours). The reason for this exceptionally long run time is because Proteinortho occupied about 85% of physical memory when applied to large-scale data, which resulted in frequent data exchange between RAM and swap space and greatly reduced the speed. In sum, these results show that SwiftOrtho is a top performer on large-scale data.

Discussion

We present SwiftOrtho, a new high performance graph based homology classification tool. Unlike most tools that only perform orthology inference, SwiftOrtho integrates all the modules necessary for a full orthology analysis, including homology search, orthology inference, and cluster analysis. SwiftOrtho is designed to analyze large-scale genomic data on a normal desktop computer in a reasonable time. In our tests, SwiftOrtho's homology search module was nearly 30 times faster than BLASTP. The orthology inference module of SwiftOrtho was nearly 500 times faster than OrthoMCL when applied to *Euk*. When applied to the large-scale dataset, *Bac*, SwiftOrtho was the only program that finished the orthology inference test on a workstation with 32GB RAM. The cluster module of SwiftOrtho using APC can handle data that are much larger than the available RAM. In our test, APC has comparable recall and accuracy, but requires considerably less memory than MCL. It should be noted that APC improved the F_1-measure score by increasing recall in most cases. With the help of these optimized modules, SwiftOrtho has successfully finished an orthology analysis of proteins from 1,760 bacterial genomes on a machine with only 4GB RAM, which makes SwiftOrho usable for large scale analyses for researchers who may not have access to expensive computational resources. SwiftOrtho is not only fast but also accurate, as shown in the results produced when running on orthobench and QfO[13, 33].

Potential implications

In summary, SwiftOrtho is a fast and accurate orthology prediction tool that can analyze a large number of sequences with minimal computational resource use. The installation and configuration of SwiftOrtho is simple and does not require the user to have any experience in database configuration. It is easy to use, as the only input required by SwiftOrtho is a FASTA format file of protein sequences with taxonomy information in the header line. SwiftOrtho can be integrated into various common pipelines where fast orthology classification is required such as pan-genome analysis, large-scale phylogenetic tree construction, and other multi-genome analyses. It is specifically suited for microbial community analyses, where large number of sequences and species are involved.

Methods

Algorithms

Here we outline the homology search, orthology inference, and clustering as implemented in SwiftOrtho.

Homology Search

SwiftOrtho employs a seed-and-extension algorithm to find homologous gene pairs [46, 47]. At the seed phase, SwiftOrtho finds candidate target sequences that share common k-mers with the query sequence. k-mer size is an important factor that affects search sensitivity and speed [38, 48]. SwiftOrtho therefore uses long (≥ 6) k-mers to accelerate search speed. At the same time, k-mer length is negatively correlated with sensitivity [38]. To compensate for the loss of sensitivity caused by increasing the k-mer size, SwiftOrtho uses two approaches: non-consecutive k-mers and reduced amino-acid alphabets. Non-consecutive k-mer seeds (known as spaced seeds), were introduced in PatternHunter [49, 19]. The main difference between consecutive seeds and spaced seeds is that the latter allow mismatches in alignment. For example, the spaced seed

101101 allows mismatches at positions 2 and 5. The total number of matched positions in a spaced seed is known as the weight, so the weight of this seed is 4. A consecutive seed can be considered as a special case of spaced seed in which its weight equal its length. Spaced seeds often provide a better sensitivity than consecutive seeds [50, 49]. Several tools such as PatternHunter, Usearch, LAST, and DIAMOND [49, 19, 39, 40, 41] have used spaced seed to increase sensitivity. PatternHunter and Usearch allow users to use custom spaced seed. The default spaced seed patterns of SwiftOrtho are 1110100010001011, 11010110111 –two spaced seeds with weight of 8– but the user may define their own spaced seeds. Seed patterns were optimized using SpEED [50] and manual inspection. The choice of the spaced seeds and default alphabet are elaborated upon in the Methods section and in the Supplementary Materials. At the extension phase, SwiftOrtho uses a variation of the Smith-Waterman algorithm [51], the k-banded Smith-Waterman or k-SWAT, which only allows for k gaps [52]. k-SWAT fills a band of cells along the main diagonal of the similarity score matrix (Figure 6B), and the complexity of k-swat is reduced to $O(k \cdot min(n, m))$, where k is the maximum allowed number of gaps.

Reduced alphabets are used to represent protein sequences using an alternative alphabet that combines several amino acids into a single representative letter, based on common physico-chemical traits [53, 54, 55]. Compared with the original alphabet of 20 amino acids, reduced alphabets usually improve sensitivity [56, 57]. At the same time, reduced alphabets also introduce less specific seeds than the original alphabet, reducing the search speed.

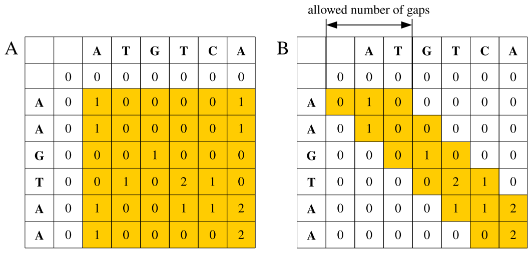

Figure 6. Comparing Standard Smith-Waterman with Banded Smith-Waterman. A. Similarity score matrix for Standard Smith-Waterman. Standard Smith-Waterman algorithm need to calculate all the entries. **B.** Similarity score matrix for Banded Smith-Waterman. Banded Smith-Waterman algorithm only need to calculate the entries on and near the diagonal.

Orthology Inference

The orthology inference step in Figure 1 shows the algorithm to infer orthologs and in-paralogs from homologs: gene A_1 in genome A and B_1 in genome B are considered to be orthologs according to the RBH rule. If the bit score between gene A_1 and A_2 in genome A is higher than that between A_1 and all its orthologs in other genomes, A_1 and A_2 are considered in-paralogs in genome A. If A_1 in genome A and B_1 in genome B are orthologs, in-paralogs of A_1 and B_1 are co-orthologs. Since orthology inference requires many queries it is better to store the data in a way that facilitates fast querying. First, SwiftOrtho sorts the data and stores it in the file system. Then, it uses binary search to query the sorted data, dramatically reducing memory usage when compared with a relational database management system or a hash table. With the help of this query system, SwiftOrtho can process data that are much larger than the computer memory.

The inferred relationships are treated as the edges of a graph. Each edge is assigned a weight for cluster analysis, where using appropriate edge-weighting metrics can improve the accuracy of cluster analysis. Gibbons [58], compared the performance of several BLAST-based edge-weighting metrics and found that the bit score has the best performance. Therefore, SwiftOrtho uses the normalized bit score as edge-weighting metric. The normalization step takes the same approach as OrthoMCL [20]. For orthologs or co-orthologs, the weight of (co-)ortholog (Figure 1) A_1 in genome A and B_1 in genome B is divided by the average edge-weight of all the (co-)orthologs between genome A and genome B. For in-paralogs, SwiftOrtho identifies a subset S of all in-paralogs in genome A, with each in-paralog A_x-A_y in subset S, A_x or A_y having at least one ortholog in another genome. The weight of each in-paralog in genome A is divided by the mean edge-weight of subset S in genome A [20].

Clustering Orthology Relationships into Orthologous Groups

SwiftOrtho provides two methods to cluster orthology relationships into orthologous groups. One is the Markov Cluster algorithm (MCL), an unsupervised clustering algorithm based on simulation of flow in graphs [21]. MCL is fast and robust on small networks and has been used by several graph-based tools [19, 59, 60, 61]. However, MCL may run out of memory when applied to a large-scale network. To reduce memory usage, we cluster each individual connected component instead of the whole network because there is no flow among components [21]. For large and dense networks a single connected component could still be too large to be loaded into memory.

For large networks, SwiftOrtho uses an Affinity Propagation Clustering algorithm (APC) [62]. The APC algorithm finds a set of centers in a network, where the centers are the actual data points and are called "exemplars". To find exemplars, APC needs to maintain two matrices: the responsibility matrix R, and the availability matrix A. The element $R_{i,k}$ in R reflects how well-suited node k is to serve as the exemplar for node i while the element $A_{i,k}$ in A reflects how appropriate node i to choose node k as its exemplar [62]. APC uses Equation 1 to update R, and Equation 2 to update A, where i, k, i', k' denote the node number, and $S_{i,k'}$ denotes the similarity between node i and node k'.

$$R_{i,k} = S_{i,k} - max_{k' \neq k}\{A_{i,k'} + S_{i,k'}\} \quad (1)$$

$$A_{i,k} = \begin{cases} min\{0, R_{k,k} + \sum_{i' \notin \{i,k\}} max\{0, R_{i',k}\}, & \text{if } i \neq k \\ \sum_{i' \neq k} max\{0, R_{i',k}\}, & \text{if } i = k \end{cases} \quad (2)$$

The node k that maximizes $A_{i,k} + R_{i,k}$ is the exemplar of node i, and each node i is assigned to its nearest exemplar. APC can update each element of matrix R and A one by one, so it is unnecessary to keep the whole matrix of R and A in memory. Generally, the time complexity of APC is $O(N^2 \cdot T)$ where N is number of nodes and T is number of iterations [62]. In this case, the time complexity is $O(E \cdot T)$, where E stands for edges which is number of orthology relationships and T is number of iterations. We implemented APC in Python, using Numba [63] to accelerate the numeric-intensive calculation parts.

Availability of source code and requirements (optional, if code is present)

The software and related information are listed below:

Project Name: SwiftOrtho

Project Home Page: https://github.com/Rinoahu/SwiftOrtho

Operating System(s): SwiftOrtho was tested on GNU/Linux distribution Ubuntu 16.04 64-bit, but we expect SwitOrtho to work on most *nix systems

Programming Language: Python

Other Requirements: Python 2.7, Python 3.7, PyPy2.7 v7.0 or higher

License: GPLv3

RRID: SCR_017122

Availability of supporting data and materials

The data sets supporting the results of this article are available in the GigaDB repository, http://dx.doi.org/10.5524/100633.

Declarations

List of abbreviations

APC: Affinity Propagation Clustering; **MCL:** Markov Clustering algorithm; **RBH:** Reciprocal Best Hit; **QFO:** Quest for Orthologs;

Ethical Approval

Not applicable

Consent for publication

Not Applicable

Competing Interests

The author(s) declare that they have no competing interests

Funding

This study has been funded, in part, by NSF award ABI 1458359. The funders had no role in the design of the study and collection, analysis, and interpretation of data and in writing the manuscript.

Author's Contributions

IF and XH conceived the study. XH wrote the software, performed the analysis. Both authors have written the manuscript.

Acknowledgements

The authors acknowledge fruitful discussions with all members of the Friedberg Lab.

Author information

IF is an Associate Professor at the Department of Veterinary Microbiology and Preventive Medicine at Iowa State University. He is also the chair of the Interdepartmental Bioinformatics and Computational Biology graduate program. XH currently is a postdoc at the Gianforte School of Computing, Montana State University.

References

1. Koonin EV. Orthologs, paralogs, and evolutionary genomics. Annu Rev Genet 2005;.
2. Fitch WM. Distinguishing Homologous from Analogous Proteins. Syst Zool 1970;19(2):99. https://academic.oup.com/sysbio/article-lookup/doi/10.2307/2412448.
3. Overbeek R, Fonstein M, D 'souza M, Pusch GD, Maltsev N. The use of gene clusters to infer functional coupling. Genetics 1999;96:2896–2901. http://www.pnas.org/content/96/6/2896.full.pdf.
4. Rivera MC, Jain R, Moore JE, Lake JA. Genomic evidence for two functionally distinct gene classes. Genetics 1998;95:6239–6244. https://www.ncbi.nlm.nih.gov/pmc/articles/PMC27643/pdf/pq006239.pdf.
5. Remm M, Storm CEVV, Sonnhammer ELLL. Automatic clustering of orthologs and in-paralogs from pairwise species comparisons. J Mol Biol 2001 dec;314(5):1041–1052. https://ac.els-cdn.com/S0022283600951970/1-s2.0-S0022283600951970-main.pdf?{_}tid=83365bc2-fd6e-11e7-a510-00000aab0f02{&}acdnat=1516403855{_}7eb11e20f0badcd297af80fe04d49166http://www.sciencedirect.com/science/article/pii/S0022283600951970?via{%}3Dihub.
6. O'Brien KP, Remm M, Sonnhammer ELL. Inparanoid: a comprehensive database of eukaryotic orthologs. Nucleic Acids Res 2005 jan;33(Database issue):D476–80. http://www.ncbi.nlm.nih.gov/pubmed/15608241http://www.pubmedcentral.nih.gov/articlerender.fcgi?artid=PMC540061.
7. Gabaldón T, Koonin EV. . Nature Reviews Genetics 2013 apr;14(5):360–366. http://www.nature.com/doifinder/10.1038/nrg3456.
8. Goodman M, Czelusniak J, Moore GW, Romero-Herrera AE, Matsuda G. Fitting the Gene Lineage into its Species Lineage, a Parsimony Strategy Illustrated by Cladograms Constructed from Globin Sequences. Syst Biol 1979;28(2):132–163. http://sysbio.oxfordjournals.org/content/28/2/132{%}5Cnhttp://sysbio.oxfordjournals.org/content/28/2/132.full.pdf{%}5Cnhttp://sysbio.oxfordjournals.org/content/28/2/132.short.
9. Kristensen DM, Wolf YI, Mushegian AR, Koonin EV. . Briefings in bioinformatics 2011 sep;12(5):379–91. http://www.ncbi.nlm.nih.gov/pubmed/21690100http://www.pubmedcentral.nih.gov/articlerender.fcgi?artid=PMC3178053.
10. Gabaldón T. Large-scale assignment of orthology: back to phylogenetics? Genome Biol 2008 oct;9(10):235. http://genomebiology.biomedcentral.com/articles/10.1186/gb-2008-9-10-235.
11. Hulsen T, Huynen MA, de Vlieg J, Groenen PMA. Benchmarking ortholog identification methods using functional genomics data. Genome Biol 2006;7(4):R31. http://www.ncbi.nlm.nih.gov/pubmed/16613613http://www.pubmedcentral.nih.gov/articlerender.fcgi?artid=PMC1557999.
12. Kuzniar A, van Ham RCHJ, Pongor S, Leunissen JAM, The quest for orthologs: finding the corresponding gene across genomes; 2008.
13. Trachana K, Larsson TA, Powell S, Chen WH, Doerks T, Muller J, et al. Orthology prediction methods: a quality assessment using curated protein families. Bioessays 2011 oct;33(10):769–80. https://www.ncbi.nlm.nih.gov/pmc/articles/PMC3193375/pdf/bies0033-0769.pdfhttp://www.ncbi.nlm.nih.gov/pubmed/21853451http://www.pubmedcentral.nih.gov/articlerender.fcgi?artid=PMC3193375.
14. Ward N, Moreno-Hagelsieb G. Quickly finding orthologs as reciprocal best hits with BLAT, LAST, and UBLAST: How much do we miss? PLoS One 2014;9(7).
15. Tatusov RL, Galperin MY, Natale DA, Koonin EV. The COG database: a tool for genome-scale analysis of protein functions and evolution. Nucleic Acids Res 2000 jan;28(1):33–36. http://www.ncbi.nlm.nih.gov/pubmed/10592175http://www.pubmedcentral.nih.gov/articlerender.fcgi?artid=PMC102395.
16. Roth ACJ, Gonnet GH, Dessimoz C. Algorithm of OMA for large-scale orthology inference. BMC Bioinformatics 2008 dec;9(1):518. http://bmcbioinformatics.biomedcentral.com/articles/10.1186/1471-2105-9-518.
17. Altenhoff AM, Glover NM, Train CM, Kaleb K, Warwick Vesztrocy A, Dylus D, et al. The OMA orthology database in 2018: Retrieving evolutionary relationships among all domains of life through richer web and programmatic interfaces. Nucleic

Acids Res 2018;.
18. Alexeyenko A, Tamas I, Liu G, Sonnhammer ELL. Automatic clustering of orthologs and inparalogs shared by multiple proteomes. In: Bioinformatics; 2006. .
19. Li M, Ma B, Kisman D, Tromp J. PatternHunter II: highly sensitive and fast homology search. Genome Inform 2003 jan;14(03):164–75. http://citeseerx.ist.psu.edu/viewdoc/download?doi=10.1.1.1.2393{&}rep=rep1{&}type=pdfpapers3://publication/uuid/B9084C91-D38A-4297-863C-B1C29E604D18{%}5Cnhttp://www.worldscientific.com/doi/abs/10.1142/S0219720004000661{%}5Cnhttp://www.ncbi.nlm.nih.gov/pubmed/157.
20. Fischer S, Brunk BP, Chen F, Gao X, Harb OS, Iodice JB, et al. Using OrthoMCL to assign proteins to OrthoMCL-DB groups or to cluster proteomes into new ortholog groups. Curr Protoc Bioinforma 2011;.
21. van Dongen S. Graph clustering by flow simulation. Graph Stimul by flow Clust 2000;PhD thesis:University of Utrecht.
22. Sonnhammer ELL, Koonin EV. Orthology, paralogy and proposed classification for paralog subtypes. Trends Genet 2002 dec;18(12):619–620. https://www.sciencedirect.com/science/article/pii/S0168952502027932?via{%}3Dihub.
23. Cannon SB, Young ND. OrthoParaMap: Distinguishing orthologs from paralogs by integrating comparative genome data and gene phylogenies. BMC Bioinformatics 2003;.
24. Cutts T, Down T, Dyer SC, Fitzgerald S, Fernandez-Banet J, Graf S, et al. Ensembl 2007. Nucl Acids Res 2007;.
25. Ruan J, Li H, Chen Z, Coghlan A, Coin LJM, Guo Y, et al. TreeFam: 2008 Update. Nucleic Acids Res 2008;.
26. Goodstadt L, Ponting CP. Phylogenetic reconstruction of orthology, paralogy, and conserved synteny for dog and human. PLoS Comput Biol 2006;.
27. Vilella AJ, Severin J, Ureta-Vidal A, Heng L, Durbin R, Birney E. EnsemblCompara GeneTrees: Complete, duplication-aware phylogenetic trees in vertebrates. Genome research 2009 feb;19(2):327–35. http://www.ncbi.nlm.nih.gov/pubmed/19029536http://www.pubmedcentral.nih.gov/articlerender.fcgi?artid=PMC2652215.
28. Chen F, Mackey AJ, Vermunt JK, Roos DS. Assessing performance of orthology detection strategies applied to eukaryotic genomes. PLoS One 2007 apr;2(4):e383. http://www.ncbi.nlm.nih.gov/pubmed/17440619http://www.pubmedcentral.nih.gov/articlerender.fcgi?artid=PMC1849888.
29. Altenhoff AM, Dessimoz C. Phylogenetic and functional assessment of orthologs inference projects and methods. PLoS Comput Biol 2009 jan;5(1):e1000262. http://dx.plos.org/10.1371/journal.pcbi.1000262.
30. Sonnhammer ELL, Östlund G. InParanoid 8: orthology analysis between 273 proteomes, mostly eukaryotic. Nucleic acids research 2015 jan;43(Database issue):D234–9. http://www.ncbi.nlm.nih.gov/pubmed/25429972http://www.pubmedcentral.nih.gov/articlerender.fcgi?artid=PMC4383983.
31. Cosentino S, Iwasaki W. SonicParanoid: Fast, accurate and easy orthology inference. Bioinformatics 2019;.
32. Lechner M, Findeiß S, Steiner L, Marz M, Stadler PF, Prohaska SJ. Proteinortho: Detection of (Co-)orthologs in large-scale analysis. BMC Bioinformatics 2011;.
33. Altenhoff AM, Boeckmann B, Capella-Gutierrez S, Dalquen DA, DeLuca T, Forslund K, et al. Standardized benchmarking in the quest for orthologs. Nat Methods 2016;.
34. Curwen V, Eyras E, Andrews TD, Clarke L, Mongin E, Searle SMJ, et al. The Ensembl automatic gene annotation system. Genome Res 2004;14(5):942–950.
35. Benson DA. GenBank. Nucleic Acids Res 2000;28(1):15–18. https://academic.oup.com/nar/article-lookup/doi/10.1093/nar/28.1.15.
36. Camacho C, Coulouris G, Avagyan V, Ma N, Papadopoulos J, Bealer K, et al. BLAST+: architecture and applications. BMC Bioinformatics 2009;.
37. Brohée S, van Helden J. Evaluation of clustering algorithms for protein-protein interaction networks. BMC Bioinformatics 2006;.
38. Kent WJ. BLAT — The BLAST -Like Alignment Tool. Genome Research 2002;12:656–664.
39. Edgar RC. Search and clustering orders of magnitude faster than BLAST. Bioinformatics 2010;26(19):2460–2461.
40. Kiełbasa SM, Wan R, Sato K, Horton P, Frith MC. Adaptive seeds tame genomic sequence comparison. Genome Res 2011;21(3):487–493.
41. Buchfink B, Xie C, Huson DH, Fast and sensitive protein alignment using DIAMOND; 2014.
42. Medlar A, Holm L. TOPAZ: Asymmetric suffix array neighbourhood search for massive protein databases. BMC Bioinformatics 2018;.
43. Rigo A, Pedroni S. PyPy ' s Approach to Virtual Machine Construction. Companion to 21st ACM SIGPLAN Symp 2006;p. 944–953.
44. Bratlie MS, Johansen J, Sherman BT, Huang DW, Lempicki RA, Drabløs F. Gene duplications in prokaryotes can be associated with environmental adaptation. BMC Genomics 2010;.
45. Katju V, Bergthorsson U, Copy-number changes in evolution: Rates, fitness effects and adaptive significance; 2013.
46. Pearson WR, Lipman DJ. Improved tools for biological sequence comparison. Proc Natl Acad Sci 1988;85(8):2444–2448. http://www.pnas.org/cgi/doi/10.1073/pnas.85.8.2444.
47. Altschul SF, Gish W, Miller W, Myers EW, Lipman DJ. Basic local alignment search tool. J Mol Biol 1990;215(3):403–410.
48. Shiryev SA, Papadopoulos JS, Schäffer AA, Agarwala R, Schaffer AA, Agarwala R. Improved BLAST searches using longer words for protein seeding. Bioinformatics 2007 nov;23(21):2949–2951. https://academic.oup.com/bioinformatics/article-lookup/doi/10.1093/bioinformatics/btm479.
49. Ma B, Tromp J, Li M. PatternHunter: faster and more sensitive homology search. Bioinformatics 2002;18(3):440–445. https://academic.oup.com/bioinformatics/article-lookup/doi/10.1093/bioinformatics/18.3.440.
50. Ilie L, Ilie S, Khoshraftar S, Bigvand AM. Seeds for effective oligonucleotide design. BMC Genomics 2011 jan;12(1):280. http://www.pubmedcentral.nih.gov/articlerender.fcgi?artid=3128067{&}tool=pmcentrez{&}rendertype=abstract.
51. Smith TF, Waterman MS. Identification of common molecular subsequences. J Mol Biol 1981 mar;147(1):195–197. http://www.ncbi.nlm.nih.gov/pubmed/7265238http://linkinghub.elsevier.com/retrieve/pii/0022283681900875.
52. Chao KM, Pearson WR, Miller W. Aligning two sequences within a specified diagonal band. Bioinformatics 1992 oct;8(5):481–487. http://www.ncbi.nlm.nih.gov/pubmed/1468007.
53. Landès C, Risler JL. Fast databank searching with a reduced amino-acid alphabet. Computer applications in the biosciences : CABIOS 1994 Jul;10(4):453–454. http://view.ncbi.nlm.nih.gov/pubmed/7804879.
54. Murphy LR, Wallqvist A, Levy RM. Simplified amino acid alphabets for protein fold recognition and implications for folding. Protein Eng Des Sel 2000 mar;13(3):149–152. https://academic.oup.com/peds/article-lookup/doi/10.1093/protein/13.3.149.
55. Peterson EL, Kondev J, Theriot JA, Phillips R. Reduced amino acid alphabets exhibit an improved sensitivity and selectivity in fold assignment. Bioinformatics (Oxford, England) 2009 Jun;25(11):1356–1362. http://dx.doi.org/10.1093/bioinformatics/btp164.
56. Edgar RC. Local homology recognition and distance

measures in linear time using compressed amino acid alphabets. Nucleic acids research 2004;32(1):380–5. http://www.ncbi.nlm.nih.gov/pubmed/14729922http://www.pubmedcentral.nih.gov/articlerender.fcgi?artid=PMC373290.

57. Ye Y, Choi JH, Tang H. RAPSearch: a fast protein similarity search tool for short reads. BMC Bioinformatics 2011 may;12(1):159. http://bmcbioinformatics.biomedcentral.com/articles/10.1186/1471-2105-12-159.
58. Gibbons TR, Mount SM, Cooper ED, Delwiche CF. Evaluation of BLAST-based edge-weighting metrics used for homology inference with the Markov Clustering algorithm. BMC Bioinformatics 2015;16(1). https://www.ncbi.nlm.nih.gov/pmc/articles/PMC4496851/pdf/12859{_}2015{_}Article{_}625.pdf.
59. Enright AJ, Van Dongen S, Ouzounis CA. An efficient algorithm for large-scale detection of protein families. Nucleic Acids Res 2002 apr;30(7):1575–1584. http://www.pubmedcentral.nih.gov/articlerender.fcgi?artid=101833{&}tool=pmcentrez{&}rendertype=abstract{%}5Cnciteulike-article-id:159967{%}5Cnhttp://dx.doi.org/10.1093/nar/30.7.1575.
60. Emms DM, Kelly S. OrthoFinder: solving fundamental biases in whole genome comparisons dramatically improves orthogroup inference accuracy. Genome Biology 2015 dec;16(1):157. http://genomebiology.com/2015/16/1/157.
61. Davis JJ, Gerdes S, Olsen GJ, Olson R, Pusch GD, Shukla M, et al. PATtyFams: Protein families for the microbial genomes in the PATRIC database. Front Microbiol 2016;7(FEB):118. http://www.ncbi.nlm.nih.gov/pubmed/26903996http://www.pubmedcentral.nih.gov/articlerender.fcgi?artid=PMC4744870.
62. Frey BJ, Dueck D. Clustering by passing messages between data points. Science 2007 feb;315(5814):972–6. http://www.ncbi.nlm.nih.gov/pubmed/17218491http://science.sciencemag.org/content/sci/315/5814/972.full.pdf.
63. Lam SK, Pitrou A, Seibert S. Numba: A LLVM-based python JIT compiler. Proc Second Work LLVM Compil Infrastruct HPC - LLVM '15 2015;p. 1–6.

Click here to access/download
Supplementary Material
Supplementary_Materials.pdf

Personal Cover

Click here to access/download;Personal Cover;SwiftOrtho Rebuttal 2.pdf

IOWA STATE UNIVERSITY
OF SCIENCE AND TECHNOLOGY

Hans Zauner, PhD
Editor, GigaScience
Oxford University Press

Iddo Friedberg, PhD
Associate Professor
College of Veterinary Medicine
Iowa State University
Ames, IA 50011
T: +1 515 294 5959
E: idoerg@iastate.edu

August 30, 2019

Dear Dr. Zauner,

Enclosed please find our revised manuscript. We are pleased that our manuscript has been accepted, and We would like to thank the reviewers and yourself for your time and effort. The comments were minor, and addressed mostly style and typographical errors.

We are happy to include the paper in the Technical Notes section. We have registered SwiftOrtho in SciCrunch.org, and added the Software Availability section, and the availability of supporting source code and requirements. We have already worked with your staff and uploaded the data to the GigaScience data repository, and referenced that in our manuscript.

Below are the reviewers' requests, and our detailed responses are in italics. We are looking forward to your feedback.

Sincerely,

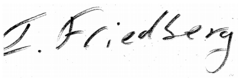

Iddo Friedberg

Reviewer 1

Overall, the comments have been addressed adequately.
I only have two remaining issues, which should be possible to address without further re-review:
- My comment on Figure 6D was apparently misunderstood. I was wondering why the y-axis needs to have a lower value below zero. Similarly, the point regarding OrthoFinder in Figure 6F indicated that visually the y-axis choice makes it look as if it were horrible, while in reality there is a ±10-20% difference.
- The documentation of the code in e.g. https://github.com/Rinoahu/SwiftOrtho/blob/master/bin/find_cluster.py could still be improved; several functions only have a one word explanation or use unclear abbreviations. Also, the widespread use of single-letter variable names make it very difficult to follow what is happening. I feel that one more round of revision would really help for future use and maintenance of the code, but I will leave this at the author's discretion.

We have now fixed the Figures to reflect the reviewer's requests. We have also redocumented the code, but have not made major changes to the variable names, etc. as that will require a new release of the software.

Reviewer 2

The authors addressed all major points raised in the first review. The inclusion of comparative benchmarks with other BLAST-like methods and orthology inference tools puts SwiftOrtho in a good perspective to other software, highlighting its advantages regarding low computational requirements to a so far unmet degree. An upgrade to Python 3 increases its durability in the near future.

Minor points:
- author contribution and acknowledgments are still missing
- line 40: As stated in the author's answer: "This approach is similar to InParanoid" The current phrasing in the manuscript implies that InParanoid is explicitly called by OrthoMCL which is not true.
- typos: line 127 (stores), line 414 (Proteinortho)

We have now fixed those errors. Thank you for pointing them out.

Reviewer 3

The authors have addressed my concerns, and appear to have covered those of the co-reviewers. The extra supplemental information on the tool comparisons is welcome. I only have minor grammatical changes to pass on:

Lines 46-47: "first perform all-vs-all sequence alignment, then constructs gene families by the sequence similarity or conserved gene neighborhood" : "first performs all-vs-all sequence alignment, then constructs gene families by sequence similarity or conserved gene neighborhood"
L67-68: "However, these tools require high performance computers to analyze large-scale data." : "However, these tools tend to require high performance computers with large memory available to analyze large-scale data"
L102-103: "Spaced seed" should be plural in both cases.
L127: "First, SwiftOrtho sorts the data and store it on hard drive." : "Firstly, SwiftOrtho sorts the data and stores it on the filesystem"
L419: "[of, For] Bac set" : "[of, For] the Bac set"
L421: "impractical to apply OMA to large-scale data set." : "impractical to apply OMA to a large-scale data set in most computational environments"
L424: "Bac set" : "the Bac set"
L430-431: "on Bac set" : "on the Bac set"
L433: "in all-vs-all homology search step" : "in the all-vs-all homology search step"
L436: "of orthology inference" : "of the orthology inference"
L437: "to Bac set" : "to the Bac set"
L440-441: "In sum, these results show that SwiftOrtho is a top performer on large-scale data." : "In summary, these results show that SwiftOrtho is the most computationally efficient on large-scale data."

Thank you for your diligence. We have now fixed these errors.
