# Supplementary material for: SwiftOrtho: A fast, memory-efficient, multiple genome orthology classifier
Source: Gigascience. 2019 Oct 24;8(10):giz118. doi: 10.1093/gigascience/giz118 (PMC6812468; doi:10.1093/gigascience/giz118)
Supplement: giz118_Supplemental_File [file giz118_supplemental_file.pdf]

Supplementary Materials to "SwiftOrtho: a Fast, Memory-Efficient, Multiple GenomeOrthology Classifier"

Methods

1 Data sets and online service used in benchmarking

Data or Service	URL
OrthoBench	http://eggnog.embl.de/orthobench2/orthobench2.all.data.tar.gz
QfO 2011	ftp://ftp.ebi.ac.uk/pub/databases/reference_proteomes/previous_releases/qfo_release-2011_04/2011_04_reference_proteomes.tar.gz
QfO service	http://orthology.benchmarkservice.org/cgi-bin/gateway.pl

Table S1: Data sets and online service used in benchmarking.

2 Sequence Alignment

2.1 Default reduced amino acid alphabet

Categories	Amino acid
1	AST
2	CFILMVY
3	DN
4	EQ
5	G
6	H
7	KR
8	P
9	W

Table S2: Default reduced amino acid table used by SwiftOrtho homology search module.

2.2 The statistics of pairwise sequence alignment

$$E = D \times M \times N \times 2^{-S'} \quad (1)$$

where D is the size of database, M and N are the length of query and reference sequences, S' is the bit score.

3 Optimization of Space Seed for SwiftOrtho

SpEED was used to find the optimized space seed and the command line is:

```
$ SpEED 8 2 .25 100
```

4 Orthology Analysis

4.1 Software

Name	Version	Source
BLASTP	2.2.27+	ftp://ftp.ncbi.nlm.nih.gov/blast/executables/rmblast/2.2.27/
OrthoMCL	2.0	https://github.com/stajichlab/OrthoMCL
FastOrtho	1.1.10	https://github.com/davidemms/OrthoFinder/releases
OrthAgogue		https://github.com/guyleonard/orthagogue
InParanoid	4.1	http://software.sbc.su.se/cgi-bin/request.cgi?project=inparanoid
SonicParanoid	1.0	http://iwasakilab.bs.s.u-tokyo.ac.jp/sonicparanoid/
MCL	v14-137	https://www.micans.org/mcl/index.html?sec_software

Table S3: Software used in orthology analysis

4.2 Parameters for running the software

The Orthology Analysis Pipeline for SwiftOrtho, OrthoMCL, FastOrtho, and OrthAgogue has three steps of homlogy search, orthology inference, and cluster analysis.

4.2.1 Homology Search

1. SwiftOrtho for *Euk*:

```
$python SwiftOrtho/bin/find_hit.py -p blastp -i input \
-d input -o output -r aa20 -e 1e-5 -s 1011111,111111 -v 1000000
/
```

2. SwiftOrtho for *QfO 2011*:

```
$python SwiftOrtho/bin/find_hit.py -p blastp -i input \
-d input -o output -r aa20 -e 1e-5 -s 11111111 -v 1000000
```

3. SwiftOrtho for *Bac*:

```
$python SwiftOrtho/bin/find_hit.py -p blastp -i input \
-d input -o output -r aa20 -e 1e-5 -s 111111 -v 1000000
```

4. BLASTP for *Euk* and *QfO 2011*:

```
$blastall -p blastp -i input -d input -o output -m 8 \
 -v 1000000 -b1000000 -e 1e-5
```

4.2.2 Orthology Inference

1. SwiftOrtho:

```
$python SwiftOrtho/bin/find_orth.py -i input \
 -c 0.5 -y 0 > output
```

2. OrthoMCL:

   We followed the instructions at the URL: `http://orthomcl.org/common/downloads/software/v2.0/UserGuide.txt`

3. FastOrtho:

```
$FastOrtho --option_file option_file.txt
```

4. OrthAgogue:

```
$orthAgogue -i input -e 5 -O output -b -c 8 \
 -dbs 1000000000 -u -A
```

4.2.3 Cluster Analysis

1. Affinity propagation algorithm:

```
$python SwiftOrtho/bin/find_clpy -i input -d 0.5 \
-a apc > output
```

2. Markov Cluster algorithm:

```
$mcl input --abc -I 1.5 -o output -te 12
```

4.3 Inparanoid, sonicparanoid, and OrthoFinder

4.3.1 OrthoFinder:

```
$mcl input --abc -I 1.5 -o output -te 12
```

4.3.2 OrthoFinder:

```
$orthofinder -f input_dir -a 12 -t 12
```

4.3.3 SonicParanoid:

```
$sonicparanoid.py -i input_dir -o output_dir -t 8 -m fast
```

4.3.4 InParanoid:

```
$inparanoid.pl fasta_file_A fasta_file_B > output
```

Results

Query(*Rattus norvegicus*)	Target(*Tetraodon nigroviridis*)	E-value	Bit score
**ENSRNOP00000063891**	**ENSTNIP00000002888**	0	764(763)
ENSRNOP00000063891	ENSTNIP00000013692	0	761(751)
ENSRNOP00000063891	ENSTNIP00000006654	0	735(736)
ENSRNOP00000063891	ENSTNIP00000009027	0	713(713)
ENSRNOP00000063891	ENSTNIP00000002673	0	700(691)
ENSRNOP00000063891	ENSTNIP00000009026	0	687(689)
ENSRNOP00000063891	ENSTNIP00000022868	0	673(701)
ENSRNOP00000063891	ENSTNIP00000001850	0	662(670)
ENSRNOP00000063891	ENSTNIP00000009028	0	658(686)
ENSRNOP00000063891	ENSTNIP00000013693	0	622(622)
ENSRNOP00000063891	ENSTNIP00000002030	0	611(625)
ENSRNOP00000063891	ENSTNIP00000009029	0	598(612)
ENSRNOP00000063891	ENSTNIP00000009813	0	585(585)
ENSRNOP00000063891	ENSTNIP00000015340	0	577(568)
ENSRNOP00000063891	ENSTNIP00000003103	0	569(569)
ENSRNOP00000063891	ENSTNIP00000005199	0	565(567)
ENSRNOP00000063891	ENSTNIP00000008595	0	560(560)

Table S4: An example of OrthoMCL and FastOrtho overestimating orthologs. The values in parentheses are the bit scores of reciprocal BLAST hits. The protein ENSRNOP00000063891 in *Rattus norvegicus* has 17 hits in *Tetraodon nigroviridis*, E-value of which are an e-value of zero. Among the 17 hits, ENSTNIP00000002888 has the highest bit score. According to the Reciprocal Best Hits (RBHs) rule, only ENSRNOP00000063891 and ENSTNIP00000002888 are true orthologs because of the highest bit score. However, OrthoMCL and FastOrtho treat all the 17 hits as RBHs because they have the same E-value of effective zero. SwiftOrtho and OrthAgogue can distinguish false RBHs because they use the bit score as edge-weighting metric. However, OrthoMCL and FastOrtho treat other hits as orthologs as they have the same zero E-value.

		**SwiftOrtho**	**OrthoMCL**	**FastOrtho**	**OrthAgogue**	**OrthoFinder**
**Homology Search**	Method	SO built-in	BLASTP			
	CPU Time(hr)	18.7	724			1,371
**Orthology Inference**	CPU Time(min)	23(10.7*)	5,175	27	44	807
	Real Time(min)	23.7(11.6*)	7,617	29	10	188
	Peak RAM(GB)	10.1(10.1*)	3.4	2.9	105.0	6.3

Table S5: Performance comparison of all orthology analysis tools on *Euk*. *:Time and RAM usage under PyPy interpreter; SO: SwiftOrtho; MCL: Markov Clustering; APC: Affinity Propagation Cluster.

		**SwiftOrtho**	**OrthoMCL**	**FastOrtho**	**OrthAgogue**	**OrthoFinder**
**Homology Search**	Method	SO built-in	BLASTP			
	CPU Time(hr)	45.88	2,995			7,500
**Orthology Inference**	CPU Time(hr)	.42(.24*)	3.08	.59	.50	13.55
	Real Time(hr)	.43(.31*)	6.8	1.78	.29	3.25
	Peak RAM(GB)	13.98(13.98*)	3.38	3.23	68.29	6.36

Table S6: Performance comparison of all orthology analysis tools on *QfO 2011*. SO: SwiftOrtho; MCL: Markov Clustering; APC: Affinity Propagation Cluster; *:Time and RAM usage under PyPy; N/A: not available.

		**SwiftOrtho**		**OrthoMCL**	**FastOrtho**	**OrthAgogue**	**OrthoFinder**
**Homology Search**	Method	SO built-in		BLASTP			N/A
	CPU Time(hr)	1,247		~30,000†			N/A
**Orthology Inference**	CPU Time(hr)	41.6(17.7*)		N/A	27.6	N/A	N/A
	Real Time(hr)	43.5(19.5*)		N/A	27.7	N/A	N/A
	Peak RAM(GB)	90.6(72.1*)		N/A	99.5	N/A	N/A
**Clustering**	Algorithm	MCL	APC	N/A	MCL	N/A	N/A
	CPU Time(hr)	41.4	12.7	N/A	57.7	N/A	N/A
	Real Time(hr)	6	26	N/A	7	N/A	N/A
	Peak RAM(GB)	81.3	39.5	N/A	67.8	N/A	N/A

Table S7: Performance comparison of all orthology analysis tools on *Bac*. SO: SwiftOrtho; MCL: Markov Clustering; APC: Affinity Propagation Cluster; *:Time and RAM usage under PyPy; †: estimated by a subset of protein sequences, N/A: not available.

Hardware				
**Platform**	**Virtual Machine**	**Desktop**	**Workstation**	**Server**
**CPU Model**	E3-1271 v3	E5-1620 v4	i7-6800K	E5-2640 v3
**CPU Frequency(GHz)**	3.6	3.5	3.4	2.6
**CPU Cores**	4	8	12	16
**RAM(GB)**	4	16	32	128
**File System**	Local Disk			Network File System
**Orthology Inference**				
**Input homologs**	8,478,732,753			
**(Co-)Orthologs**	876,766,940			
**In-Paralogs**	622,292			
**CPU Time (hr)**	37	37.6	36	41.6
**Real Time(hr)**	69	46.5	41.7	43.5
**Peak RAM (GB)**	2.58	11	19.3	90.6
**Clustering**				
**Algorithm**	Affinity Propagation			
**Orthologous Groups**	167,355			
**CPU Time (hr)**	7.3	10	12.1	12.7
**Real Time (hr)**	56.5	60.5	35.7	26
**Peak RAM (GB)**	3.6	14.8	25	39.5

Table S8: Orthology Analysis of SwiftOrtho on *Bac* running on systems with different specifications. SwiftOrtho can perform orthology inference and cluster analysis on a machine with only 4GB RAM. The peak memory usage of SwiftOrtho is scalable, which guarantees that it will run even on low-memory system.

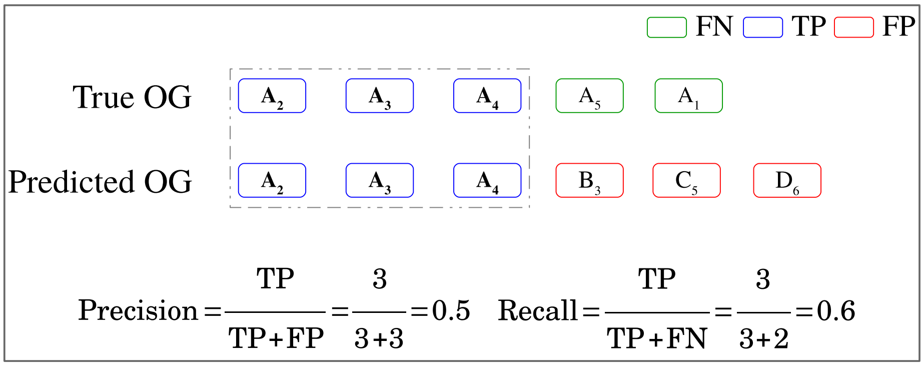

Figure S1: **Definition of Precision and Recall for Predicted Orthologous Groups. True OG:** Orthologous Groups from Orthobench dataset; **Predicted OG:** Predicted Orthologous Groups by SwiftOrtho. **TP:** True Positive, the identical genes between True OG and Predicted OG; **FN:** False Negative, genes that only exist in True OG; **FP:** False Positive, genes that only exist in Predicted OG.

Aligner	Command line	CPU Time(hr)	Uniq Hits	Shared Hits with BLASTP
**BLAST**	blastall -p blastp -i query.faa -d query.faa -o output -e 1e-5 -b 1000000 -v 1000000 -m 8 -a 16	724	297,107,872	100.00%
**SwiftOrtho**	python find_hit.py -p blastp -i query.faa -d query.faa -o output -a 16 -r aa20 -s 1011111,111111 -v 1000000	18.7	162,695,330	52.72%
**DIAMOND**	diamond blastp -d query.faa -f 6 -p 16 -q query.faa -k 100000 -e 1e-5 -o output –sensitive	5.75	72,129,761	24.02%
**DIAMOND (sensitive)**	diamond blastp -d query.faa -f 6 -p 16 -q query.faa -k 100000 -e 1e-5 -o output	14.85	133,811,102	43.82%
**DIAMOND (more-sensitive)**	diamond blastp -d query.faa -f 6 -p 16 -q query.faa -k 100000 -e 1e-5 -o output –more-sensitive	19.42	179,267,720	58.30%
**BLAT**	blat -prot query.faa query.faa -out=blast8 -stepSize=1 output	28.15	18,659,628	28.34%
**Topaz**	topaz search -f query.faa -p database -T 16 –blasttab -F -E 1e-5 -H 1000000	44.18	107,859,744	33.48%
**Usearch**	usearch -ublast query.faa -db database.udb -evalue 1e-5 -accel 0.5 -threads 16 -blast6out output.m8	0.328	2,720,464	0.88%
**LAST**	lastal -E1e-5 db query.faa -N 1000000 -f BlastTab -P 16	0.961	3,139,310	2.97%

Table S9: A comparison of SwiftOrtho with several homology protein search tools.

		SwiftOrtho	Sonicparanoid	OMA	Proteinortho6
**Command line**		See Supplementary Materials	$sonicparanoid -i input_dir -o output_dir -m fast -t 16	$oma # we use default parameters.drw	proteinortho6.pl s1.faa s2.faa s3.faa
Euk	**CPU Time (hr)**	19.08	14.94	N/A	12.6
	**Peak RAM (GB)**	10.1	2.88	N/A	7.2
QfO 2011	**CPU Time (hr)**	46.3	22.36	N/A	46.68
	**Peak RAM (GB)**	13.98	2.83	N/A	2.8
Bac	**CPU Time (hr)**	1301.3	N/A	N/A	2,528⋆
	**Peak RAM (GB)**	90.6	N/A	N/A	106
**Hardware**	E5-2640 v3 at 2.6 GHz, 128 GB RAM				

Table S10: Performance comparison of SwiftOrtho with other orthology prediction pipelines; **OMA**: Orthologous MAtrix standalone, OMA did not finish orthology analysis on any data sets in reasonable time; SonicParanoid aborted when applied to *Bac* set; ⋆**:** the total CPU time by using LAST in homology search step. **N/A:** Not Available.
