# Supplementary material for: SwiftOrtho: A fast, memory-efficient, multiple genome orthology classifier
Source: Gigascience. 2019 Oct 24;8(10):giz118. doi: 10.1093/gigascience/giz118 (PMC6812468; doi:10.1093/gigascience/giz118)
Supplement: giz118_Response_to_Reviewer_Comments_Original_Submission [file giz118_response_to_reviewer_comments_original_submission.pdf]

Author's Response To Reviewer Comments

Close

Dear Dr. Zauner,

Enclosed please find our revised manuscript. We would like to thank the reviewers and yourself for the large amount of time and effort that were spent reading and commenting on the manuscript. We are grateful for this effort, and we have addressed all comments in detail. We would like to stress that SwiftOrtho's strength lies both in its modular versatility, and in its low consumption of computational resources, making it especially suitable for low- and medium budget labs, but is easy and accurate enough to be used universally.

We have upgraded the software to Python 3, and rewrote the code to conform with PEP-8. We have also compared with all the other tools requested, and some others, including DIAMOND, Usearch, TOPAZ, LAST and BLAT.

We are happy to include the paper in the Technical Notes section. We have registered SwiftOrtho in SciCrunch.org, and added the Software Availability section, and the availability of supporting source code and requirements.

Attached are the reviewers' requests, and our detailed responses are in italics. We are looking forward to your feedback.

Sincerely,

Iddo Friedberg

Close
