# Supplementary material for: SwiftOrtho: A fast, memory-efficient, multiple genome orthology classifier
Source: Gigascience. 2019 Oct 24;8(10):giz118. doi: 10.1093/gigascience/giz118 (PMC6812468; doi:10.1093/gigascience/giz118)
Supplement: giz118_Response_to_Reviewer_Comments_Revision_2 [file giz118_response_to_reviewer_comments_revision_2.pdf]

Author's Response To Reviewer Comments

Close

Dear Dr. Zauner,

Enclosed please find our revised manuscript. We are pleased that our manuscript has been accepted, and We would like to thank the reviewers and yourself for your time and effort. The comments were minor, and addressed mostly style and typographical errors.

We are happy to include the paper in the Technical Notes section. We have registered SwiftOrtho in SciCrunch.org, and added the Software Availability section, and the availability of supporting source code and requirements. We have already worked with your staff and uploaded the data to the GigaScience data repository, and referenced that in our manuscript.

Below are the reviewers' requests, and our detailed responses are in italics. We are looking forward to your feedback.

Sincerely,

Iddo Friedberg

Reviewer 1

Overall, the comments have been addressed adequately.
I only have two remaining issues, which should be possible to address without further re-review:
- My comment on Figure 6D was apparently misunderstood. I was wondering why the y-axis needs to have a lower value below zero. Similarly, the point regarding OrthoFinder in Figure 6F indicated that visually the y-axis choice makes it look as if it were horrible, while in reality there is a ±10-20% difference.
- The documentation of the code in e.g. https://github.com/Rinoahu/SwiftOrtho/blob/master/bin/find_cluster.py could still be improved; several functions only have a one word explanation or use unclear abbreviations. Also, the widespread use of single-letter variable names make it very difficult to follow what is happening. I feel that one more round of revision would really help for future use and maintenance of the code, but I will leave this at the author's discretion.

We have now fixed the Figures to reflect the reviewer's requests. We have also redocumented the code, but have not made major changes to the variable names, etc. as that will require a new release of the software.

Reviewer 2

The authors addressed all major points raised in the first review. The inclusion of comparative benchmarks with other BLAST-like methods and orthology inference tools puts SwiftOrtho in a good perspective to other software, highlighting its advantages regarding low computational requirements to a so far unmet degree. An upgrade to Python 3 increases its durability in the near future.

Minor points:
- author contribution and acknowledgments are still missing
- line 40: As stated in the author's answer: "This approach is similar to InParanoid" The current phrasing in the manuscript implies that InParanoid is explicitly called

by OrthoMCL which is not true.
- typos: line 127 (stores), line 414 (Proteinortho)

We have now fixed those errors. Thank you for pointing them out.

Reviewer 3

The authors have addressed my concerns, and appear to have covered those of the co-reviewers. The extra supplemental information on the tool comparisons is welcome. I only have minor grammatical changes to pass on:

Lines 46-47: "first perform all-vs-all sequence alignment, then constructs gene families by the sequence similarity or conserved gene neighborhood" : "first performs all-vs-all sequence alignment, then constructs gene families by sequence similarity or conserved gene neighborhood"
L67-68: "However, these tools require high performance computers to analyze large-scale data." : "However, these tools tend to require high performance computers with large memory available to analyze large-scale data"
L102-103: "Spaced seed" should be plural in both cases.
L127: "First, SwiftOrtho sorts the data and store it on hard drive." : "Firstly, SwiftOrtho sorts the data and stores it on the filesystem"
L419: "[of, For] Bac set" : "[of, For] the Bac set"
L421: "impractical to apply OMA to large-scale data set." : "impractical to apply OMA to a large-scale data set in most computational environments"
L424: "Bac set" : "the Bac set"
L430-431: "on Bac set" : "on the Bac set"
L433: "in all-vs-all homology search step" : "in the all-vs-all homology search step"
L436: "of orthology inference" : "of the orthology inference"
L437: "to Bac set" : "to the Bac set"
L440-441: "In sum, these results show that SwiftOrtho is a top performer on large-scale data." : "In summary, these results show that SwiftOrtho is the most computationally efficient on large-scale data."

Thank you for your diligence. We have now fixed these errors.

Close
