# Supplementary material for: SwiftOrtho: A fast, memory-efficient, multiple genome orthology classifier
Source: Gigascience. 2019 Oct 24;8(10):giz118. doi: 10.1093/gigascience/giz118 (PMC6812468; doi:10.1093/gigascience/giz118)
Supplement: giz118_Reviewer_1_Report_Original_Submission — Marnix Medema -- 3/14/2019 Reviewed [file giz118_reviewer_1_report_original_submission.pdf]

Reviewer Report

Title: SwiftOrtho: a Fast, Memory-Efficient, Multiple Genome Orthology Classifier

Version: Original Submission **Date:** 3/14/2019

Reviewer name: Marnix Medema

Reviewer Comments to Author:

The paper of Hu and Friedberg presents an interesting new method for large-scale identification of orthologous groups without the need for large-scale compute servers. The use of spaced seeds and reduced amino alphabets are innovative and interesting, and the method has a high precision across benchmarks, compared to its competitors. While the method is interesting and has added value, I think there are a number of ways in which the paper and the tool could be improved. I have ordered my recommendations in a few different categories.

Scientific:

*   The key point of the paper seems to be that SwiftOrtho requires less memory and CPU compared to other tools, yet there is no figure or table that compares these characteristics across tools for datasets of different sizes. I would strongly recommend adding this to actually show the difference with each of the other tools.
*   Line 227: SwiftOrtho is compared to BlastP, but not to other fast equivalents such as Diamond or Usearch. I think this comparison should be added, to show the added value of the method compared to the current state of the art.

General writing:

*   The introduction does not read fluently. It is a bit 'staccato', without clear connections and transitions between the parts. I think this can use some editing to more clearly describe the field, its relevance (which is only explained in vague terms at the moment), the state of the art, the challenge and the contribution by the authors.
*   It is surprising that the methodology for orthology classification (which is what makes it fast and memore-efficient) is not mentioned in the abstract.
*   Line 14: Inparanoid is mentioned without introducing it.
*   The figures could use a make-over, e.g. by removing horizontal/vertical skewing, changing fonts and applying a more pleasing color scheme.
*   It would be very helpful to have a flowchart-like figure that outlines the different steps taken by the SwiftOrtho algorithm.
*   Some of the figures (e.g., Fig 3a) are unnecessary, as they explain things that can safely be assumed to be textbook knowledge.
*   The use of horizontal scales in Figure 6 is puzzling, e.g. the -0.200 lower end for Fig. 6D. Also, this makes OrthoFinder looks unrealistically bad in panel F.

Grammar/spelling:

A thorough round of copy-editing would be highly recommended, as there are several spelling/grammar/style issues throughout. Some examples are:

*   Line 25: limitation -> limiting factor
*   Line 51: data set -> data sets (also in line 46)
*   Line 401: the sentence is broken.
*   Figure legend of Figure 5: OrthFinder -> OrthoFinder, 'can not apply' -> 'cannot be applied'

Code/software:
*   In my honest opinion, the code needs some serious refactoring; it has many global variables (code outside functions/classes), has several commented out sections, largely lacks proper documentation (with docstrings etc.; see https://www.python.org/dev/peps/pep-0008/) and has many functions that are far too long (and need to be broken up into smaller ones). Additionally, implementing tests would ensure that the code functions as intended.
*   The readme should detail which versions are needed of the packages that are required.
*   SwiftOrtho is written and distributed in Python 2.7, which is going to be retired very soon (see https://pythonclock.org/). Updating to Python3 is highly recommended for durability of the software. This is not difficult to do.

Level of Interest

Please indicate how interesting you found the manuscript: Choose an item.

Quality of Written English

Please indicate the quality of language in the manuscript: Choose an item.

Declaration of Competing Interests

Please complete a declaration of competing interests, considering the following questions:

- Have you in the past five years received reimbursements, fees, funding, or salary from an organisation that may in any way gain or lose financially from the publication of this manuscript, either now or in the future?
- Do you hold any stocks or shares in an organisation that may in any way gain or lose financially from the publication of this manuscript, either now or in the future?
- Do you hold or are you currently applying for any patents relating to the content of the manuscript?
- Have you received reimbursements, fees, funding, or salary from an organization that holds or has applied for patents relating to the content of the manuscript?
- Do you have any other financial competing interests?
- Do you have any non-financial competing interests in relation to this paper?

If you can answer no to all of the above, write 'I declare that I have no competing interests' below. If your reply is yes to any, please give details below.

I declare that I have no competing interests.

I agree to the open peer review policy of the journal. I understand that my name will be included on my report to the authors and, if the manuscript is accepted for publication, my named report including any attachments I upload will be posted on the website along with the authors' responses. I agree for my report to be made available under an Open Access Creative Commons CC-BY license (http://creativecommons.org/licenses/by/4.0/). I understand that any comments which I do not wish to be included in my named report can be included as confidential comments to the editors, which will not be published.

Choose an item.

To further support our reviewers, we have joined with Publons, where you can gain additional credit to further highlight your hard work (see: https://publons.com/journal/530/gigascience). On publication of this paper, your review will be automatically added to Publons, you can then choose whether or not to claim your Publons credit. I understand this statement.

Yes Choose an item.
