# Supplementary material for: SwiftOrtho: A fast, memory-efficient, multiple genome orthology classifier
Source: Gigascience. 2019 Oct 24;8(10):giz118. doi: 10.1093/gigascience/giz118 (PMC6812468; doi:10.1093/gigascience/giz118)
Supplement: giz118_Reviewer_2_Report_Original_Submission — Marcus Lechner, Ph.D. -- 3/22/2019 Reviewed [file giz118_reviewer_2_report_original_submission.pdf]

Reviewer Report

Title: SwiftOrtho: a Fast, Memory-Efficient, Multiple Genome Orthology Classifier

Version: Original Submission **Date:** 3/22/2019

Reviewer name: Marcus Lechner, Ph.D.

Reviewer Comments to Author:

Hu and Friedberg present an orthology prediction tool named SwiftOrtho. It is geared towards low memory and CPU usage in order to enable large-scale analyses including orthology inference and clustering, which is a desirable goal in the field. The tool is written in Python and accessible on GitHub where it is also well documented. Overall, two new concepts are introduced to orthology prediction. First, an alternative algorithm for homology search which replaces the classical BLAST algorithm. Second, the application of the "Affinity Propagation Clustering algorithm" (APC) which replaces the well established but rather memory-intense MCL algorithm.
While the authors exemplify good prediction and recall characteristics compared to some other approaches, the principal argument of SwiftOrtho beeing a faster and more memory efficient tool than currently available is not convincingly demonstrated. There are already published orthology tools geared in the same direction. The few numbers presented on runtime and memory usage do not sound like a major improvement from my experience. Comparative benchmarks are missing in this respect. Similarly, the presentation of the current state of the field appears rather limited and the choice of alternative tools or example data sets is outdated. Overall, the provided data did not convince me.
Major issues:
1. Runtime and memory usage should be benchmarked not only versus OrthoMCL but also against more recent tools that cover homology search and clustering. I namely suggest SonicParanoid (cited but not benchmarked), Proteinortho6, and the standalone tool provided for OMA. Ideally, the two steps (homology search and clustering) could be evaluated separately.
2. BLAST is the golden standard in the field regarding sensitivity and specificity but not with respect to runtime or memory requirements. While I like the major concept, there are plenty of options besides the presented "built-in module" for homology search. It needs to be put into perspective. Ward, et al. (cited) for instance suggests BLAT, LAST, and UBLAST. Other promising BLAST-drop-ins are e.g. topaz and diamond. Besides, a loss of 24% of all RBHs when using the built-in BLAST alternative (line 238) is way more than what I would have expected from most of these other alternatives.
3. Please use more recent tools and data sets. For instance, BLASTP v2.2.26 was published in 2012, which does not represent the current level of optimizations. The current version is 2.8.1 (2018). OrthoMCL is available at version 5 since 2011 (version 2 was used). Similarly, the QfO 2011 data set is deprecated since 2017.
4. For orthology inference, the data is stored somewhere in a sorted manner such that one "can process data that are much larger than the computer". Where is it stored? Temporary files on the hard drive? This is mentioned for the MCL clustering. How does your APC-implementation behave in this respect?
5. The concepts of genomes, DNA sequences and amino acid sequences of proteins need to be sorted

throughout the manuscript including the title. E.g. You did not analyze 1760 bacterial genomes but proteomes. Figure 2 shows nucleic acid alignments while the discussed context is about amino acids.
6. The concepts of (co-)orthology and (in-)paralogy should be explained in some more detail. E.g. the likelihood to maintain the same biological function is much higher for orthologs than for paralogs. The term "in-paralog" should be explained with its first use and also put in context to out-paralogs.
7. I did not quite get the spaced seed concept. Why would the user need/want to define at which position of a k-mer mismatches are allowed? This needs to be elaborated in more detail. I also find the respective binary vector given as parameter (-s) somewhat strange for a tool that is otherwise designed with easy use in mind (which I really appreciate). How much does it actually effect the results?
8. How is the e-value calculated for the "built-in module" (homology search)?
9. When comparing different algorithms, be fair and make sure to use the same or equivalent parameters (as far as this is possible). The OrthoFinder e-value threshold was 1e-3 while the other tools were used with 1e-5.
10. One of the two major selling points is the APC algorithm. It seems, however, not to be set as the default algorithm. The user can choose between this an MCL. Manuscript and tool description could use some information on this topic (when to use what). Is there any parameter for APC similar to the inflation parameter for MCL? Until multi-threading is implemented for APC, it looks more like a low-memory backup while it appears to be a fully fletched clustering alternative. What about simply running the clustering of several connected groups in parallel? In times with plenty of CPU-cores, it is the overall runtime that counts rather the bare CPU time.
11. How is the RBH implemented in SwiftOrtho? Usually, not only the single best hit is used but also very close ones. This might be an alternative reason for the lower number of orthology relationships (line 243).
12. The underlying data sets, as well as the results from which precision and recall scores were calculated, should be made available online. It is not trivial to reproduce e.g. the initial RBH graph from 1760 species which is necessary to evaluate the clustering algorithm. How was the data chosen?
13. I could hardly follow the APC formulas. Important details are missing, e.g. that matrices are initialized with 0. What is the difference between a well-suited node and an appropriate node (lines below line 131)? A calculation example would be useful. In general, a reference or proof is required for the algorithm and the assumptions made (e.g. line 132). APC requires less memory, what are other advantage and disadvantage in general?
Minor issues:
- Why is there a need to perform large-scale orthology predictions with only 4 GB of memory? Even consumer PCs have at least 8 GB installed.
- Line 37: If I'm not mistaken, OrthoMCL does not use the Inparanoid algorithm even though its implementation behaves similarly when used with only two genomes.
- Line 345: It might be advisable to remove different protein isoforms from the data set beforehand. Usually, these are indicated. If in doubt, only the longest variant can be used.
- As the BLAST+ implementation is used, its paper should be cited as well: Camacho C., Coulouris G., Avagyan V., Ma N., Papadopoulos J., Bealer K., & Madden T.L. (2008) "BLAST+: architecture and applications." BMC Bioinformatics 10:421.
Notes:

- Users typically download a single fasta file for each species of interest from databases. For use with SwiftOrtho they are required to merge them to a single file and adding a taxon code. While this might be trivial for experienced users, it is an unnecessary obstacle for less versed users. Moreover, this strategy of an all-vs-all BLAST increases memory requirements over a species-by-species implementation. The many output files (discussed in the manuscript) could be merged.
- line 33: "and and"
- line 102: ". if"
- line 401: sentence was not finished
- citation 11 contains an unnecessary long link
- author contribution and acknowledgments are missing

Level of Interest

Please indicate how interesting you found the manuscript: Choose an item.

Quality of Written English

Please indicate the quality of language in the manuscript: Choose an item.

Declaration of Competing Interests

Please complete a declaration of competing interests, considering the following questions:

- Have you in the past five years received reimbursements, fees, funding, or salary from an organisation that may in any way gain or lose financially from the publication of this manuscript, either now or in the future?
- Do you hold any stocks or shares in an organisation that may in any way gain or lose financially from the publication of this manuscript, either now or in the future?
- Do you hold or are you currently applying for any patents relating to the content of the manuscript?
- Have you received reimbursements, fees, funding, or salary from an organization that holds or has applied for patents relating to the content of the manuscript?
- Do you have any other financial competing interests?
- Do you have any non-financial competing interests in relation to this paper?

If you can answer no to all of the above, write 'I declare that I have no competing interests' below. If your reply is yes to any, please give details below.

I declare that I have no competing interests.

I agree to the open peer review policy of the journal. I understand that my name will be included on my report to the authors and, if the manuscript is accepted for publication, my named report including any

attachments I upload will be posted on the website along with the authors' responses. I agree for my report to be made available under an Open Access Creative Commons CC-BY license (http://creativecommons.org/licenses/by/4.0/). I understand that any comments which I do not wish to be included in my named report can be included as confidential comments to the editors, which will not be published.

Choose an item.

To further support our reviewers, we have joined with Publons, where you can gain additional credit to further highlight your hard work (see: https://publons.com/journal/530/gigascience). On publication of this paper, your review will be automatically added to Publons, you can then choose whether or not to claim your Publons credit. I understand this statement.

Yes Choose an item.
