# Supplementary material for: SwiftOrtho: A fast, memory-efficient, multiple genome orthology classifier
Source: Gigascience. 2019 Oct 24;8(10):giz118. doi: 10.1093/gigascience/giz118 (PMC6812468; doi:10.1093/gigascience/giz118)
Supplement: giz118_Reviewer_3_Report_Original_Submission — Robert Davey -- 3/29/2019 Reviewed [file giz118_reviewer_3_report_original_submission.pdf]

Reviewer Report

Title: SwiftOrtho: a Fast, Memory-Efficient, Multiple Genome Orthology Classifier

Version: Original Submission **Date:** 3/29/2019

Reviewer name: Robert Davey

Reviewer Comments to Author:

The authors present SwiftOrtho, a lightweight and modular homology detection and classification tool. They correctly state that the current tools available for homology searching often involve all-against-all pairwise comparisons, meaning the compute and RAM requirements can be large, and these analyses can take hours or days depending on the number of species and genes. The authors carry out a number of comparative analyses to demonstrate the efficacy of SwiftOrtho. They present evidence that their tool is comparable to others in terms of accuracy via various metrics, but accuracy is sacrificed to achieve very low RAM use to allow the tool to be run on low power computers. This is the key novelty of this application - being able to access these kinds of tools on a broader range of compute options is useful. The modularity of SwiftOrtho is beneficial to allow various homology analyses to be used in place of SwiftOrtho's homology module, which can help with longevity of the tool.

For the most part, the paper is well-written, has few grammatical and typography errors, and is structured so that the authors' processes are easily followed. The code and data used in the manuscript are freely available.

Specific comments:

The tool is written in Python 2.7. This version is due to be unsupported in January 2020. The tool should be updated within the next year to support Python 3.

Whilst there are two main methods of ortholog identification (tree and graph), these are not mutually exclusive and some approaches use both (Ensembl Compara / GeneSeqToFamily for example). This should be stated in the manuscript.

The authors do not present the rationale for using SonicParanoid and InParanoid only on the QfO 2011 dataset rather than all datasets, apart from using it as a control. This should be stated.

In the abstract, the authors use the word "costly" to describe computational clusters capable of carrying out these types of analysis. Whilst bigger HPC/HTC clusters do indeed make the job quicker, these do not necessarily have to be costly. Indeed, the cost is never explored in the paper. I suggest removing the subjectivity by omitting "costly" in the abstract.

The authors also state that upwards of 100GB RAM for OrthoAgogue to run "exceeds most workstations and servers". This isn't true for the current (March 2019) situation. Building a server 128GB RAM is fairly trivial these days, and can easily cost less than a 2019 MacBook. Similarly, renting a memory-optimised ~128GB server (e.g. r4.4xlarge VM instance type) from Amazon Web Services is easy. This sentence should be reworded to reflect that whilst laptops and desktops do not have this RAM availability, it is certainly not uncommon in "most servers".

General grammatical fixes(line numbers shown):

30: "and and"
31: "or COG" -> "(COG)"
46: "on large dataset" -> "on large datasets"
51: "large data set" -> "large datasets"
71: When describing the two sensitivity compensation mechanisms (line 71) it is inferred that the authors will describe both. Consider removing the "Another method" sentence, and merging it with the next, e.g. "Reduced amino acid alphabets are also used to mitigate the loss of sensitivity be representing...." etc.
114: "as edge-weighting" -> "as the edge-weighting"
115: "step take" -> "step takes"
115: remove colon and replace with full stop
126, 129: Both lines start with "However". Consider removing the repetition to make the comparison clearer (probably the second "however" isn't needed)
After 131: the paragraph "For the large networks" contains "node i" and "node k" that aren't italicised.
401: Incomplete sentence: "be used to ..."
Code repository and tests:

The software installs and runs as intended.
The author should update the readme file with the following corrections:
- Example commands are showing missing input file (we used ref.fsa from the example)
- Example command for find_hit.py is missing the "-p" parameter
- Installation instructions are missing "pip install cffi" command in readme file

The author should add available options for any of the command line parameters (i.e. -p in find_hit.py) in the help message when running the tool.
Most of the times SwiftOrtho tool is unable to recognise paralogs when orthologs are detected. SwiftOrtho only recognises in-paralogs if the bit score between two paralogs is higher than orthologs. This might be covered by the use of the term "in-paralogs", but it's not explicitly clear from the manuscript. This should be addressed.
We were not able to run SwiftOrtho successfully when supplying data for the gene BRAT1 from (chimp, human, pig, dog, and rat) containing within-species paralogs from chimp. In this case, find_orth.py script fails with the following error:

```
Traceback (most recent call last):
 File "../SwiftOrtho/bin/find_orth.py", line 551, in
   st, ed, pairs = binary_search(Sqco, [qip, sip], lambda x: x.split('\t', 3)[:2])
 File "../SwiftOrtho/bin/find_orth.py", line 403, in binary_search
   left = m - 1
UnboundLocalError: local variable 'm' referenced before assignment
```

We are happy to raise an issue in Github for this issue if required.

Level of Interest

Please indicate how interesting you found the manuscript: Choose an item.

Quality of Written English

Please indicate the quality of language in the manuscript: Choose an item.

Declaration of Competing Interests

Please complete a declaration of competing interests, considering the following questions:

- Have you in the past five years received reimbursements, fees, funding, or salary from an organisation that may in any way gain or lose financially from the publication of this manuscript, either now or in the future?
- Do you hold any stocks or shares in an organisation that may in any way gain or lose financially from the publication of this manuscript, either now or in the future?
- Do you hold or are you currently applying for any patents relating to the content of the manuscript?
- Have you received reimbursements, fees, funding, or salary from an organization that holds or has applied for patents relating to the content of the manuscript?
- Do you have any other financial competing interests?
- Do you have any non-financial competing interests in relation to this paper?

If you can answer no to all of the above, write 'I declare that I have no competing interests' below. If your reply is yes to any, please give details below.

I declare that I have no competing interests

I agree to the open peer review policy of the journal. I understand that my name will be included on my report to the authors and, if the manuscript is accepted for publication, my named report including any attachments I upload will be posted on the website along with the authors' responses. I agree for my report to be made available under an Open Access Creative Commons CC-BY license (http://creativecommons.org/licenses/by/4.0/). I understand that any comments which I do not wish to be included in my named report can be included as confidential comments to the editors, which will not be published.

Choose an item.

To further support our reviewers, we have joined with Publons, where you can gain additional credit to further highlight your hard work (see: https://publons.com/journal/530/gigascience). On publication of this paper, your review will be automatically added to Publons, you can then choose whether or not to claim your Publons credit. I understand this statement.

Yes Choose an item.
