# Supplementary material for: SwiftOrtho: A fast, memory-efficient, multiple genome orthology classifier
Source: Gigascience. 2019 Oct 24;8(10):giz118. doi: 10.1093/gigascience/giz118 (PMC6812468; doi:10.1093/gigascience/giz118)
Supplement: giz118_Reviewer_3_Report_Revision_1 — Robert Davey -- 7/16/2019 Reviewed [file giz118_reviewer_3_report_revision_1.pdf]

Reviewer Report

Title: SwiftOrtho: a Fast, Memory-Efficient, Multiple Genome Orthology Classifier

Version: Revision 1 **Date:** 7/16/2019

Reviewer name: Robert Davey

Reviewer Comments to Author:

The authors have addressed my concerns, and appear to have covered those of the co-reviewers. The extra supplemental information on the tool comparisons is welcome. I only have minor grammatical changes to pass on:
Lines 46-47: "first perform all-vs-all sequence alignment, then constructs gene families by the sequence similarity or conserved gene neighborhood" : "first performs all-vs-all sequence alignment, then constructs gene families by sequence similarity or conserved gene neighborhood"
L67-68: "However, these tools require high performance computers to analyze large-scale data." : "However, these tools tend to require high performance computers with large memory available to analyze large-scale data"
L102-103: "Spaced seed" should be plural in both cases.
L127: "First, SwiftOrtho sorts the data and store it on hard drive." : "Firstly, SwiftOrtho sorts the data and stores it on the filesystem"
L419: "[of, For] Bac set" : "[of, For] the Bac set"
L421: "impractical to apply OMA to large-scale data set." : "impractical to apply OMA to a large-scale data set in most computational environments"
L424: "Bac set" : "the Bac set"
L430-431: "on Bac set" : "on the Bac set"
L433: "in all-vs-all homology search step" : "in the all-vs-all homology search step"
L436: "of orthology inference" : "of the orthology inference"
L437: "to Bac set" : "to the Bac set"
L440-441: "In sum, these results show that SwiftOrtho is a top performer on large-scale data." : "In summary, these results show that SwiftOrtho is the most computationally efficient on large-scale data."

Level of Interest

Please indicate how interesting you found the manuscript: Choose an item.

Quality of Written English

Please indicate the quality of language in the manuscript: Choose an item.

Declaration of Competing Interests

Please complete a declaration of competing interests, considering the following questions:

- Have you in the past five years received reimbursements, fees, funding, or salary from an organisation that may in any way gain or lose financially from the publication of this manuscript, either now or in the future?
- Do you hold any stocks or shares in an organisation that may in any way gain or lose financially from the publication of this manuscript, either now or in the future?
- Do you hold or are you currently applying for any patents relating to the content of the manuscript?
- Have you received reimbursements, fees, funding, or salary from an organization that holds or has applied for patents relating to the content of the manuscript?
- Do you have any other financial competing interests?
- Do you have any non-financial competing interests in relation to this paper?

If you can answer no to all of the above, write 'I declare that I have no competing interests' below. If your reply is yes to any, please give details below.

I declare that I have no competing interests.

I agree to the open peer review policy of the journal. I understand that my name will be included on my report to the authors and, if the manuscript is accepted for publication, my named report including any attachments I upload will be posted on the website along with the authors' responses. I agree for my report to be made available under an Open Access Creative Commons CC-BY license (http://creativecommons.org/licenses/by/4.0/). I understand that any comments which I do not wish to be included in my named report can be included as confidential comments to the editors, which will not be published.

Choose an item.

To further support our reviewers, we have joined with Publons, where you can gain additional credit to further highlight your hard work (see: https://publons.com/journal/530/gigascience). On publication of this paper, your review will be automatically added to Publons, you can then choose whether or not to claim your Publons credit. I understand this statement.

Yes Choose an item.
